# A Digital Inclusion Intervention to Improve Access to a Digital Health Intervention Among Digitally Excluded Adults: Mixed Methods Pilot Randomized Controlled Trial

**DOI:** 10.2196/91438

**Published:** 2026-04-16

**Authors:** Christy Walklin, Juliet Briggs, Siobhan Freeman, Emmanuel Mangahis, Camila Dias, Sunil Bhandari, Kate Bramham, James O Burton, Jackie Campbell, Philip A Kalra, Jamie Macdonald, Maarten W Taal, David C Wheeler, Sharlene A Greenwood, Hannah M L Young

**Affiliations:** 1 Renal Therapies Department, King's College Hospital, Denmark Hill, London, SE5 9RS, England, United Kingdom, +44 020 8194 7470; 2 Department of Nephrology, Hull University Teaching Hospitals NHS Trust, Hull, England, United Kingdom; 3 Department of Cardiovascular Sciences, University of Leicester, Leicester, England, United Kingdom; 4 Faculty of Health, Sport and Behavioural Sciences, University of Northampton, Northampton, England, United Kingdom; 5 Department of Nephrology, Salford Royal Hospital, Salford, England, United Kingdom; 6 Institute for Applied Human Physiology, Bangor University, Bangor, Wales, United Kingdom; 7 School of Medicine, Centre for Kidney Research and Innovation, University of Nottingham, Nottingham, England, United Kingdom; 8 Centre for Kidney and Bladder Health, University College London, London, England, United Kingdom; 9 Renal Sciences, Faculty of Life Sciences and Medicine, King's College London, London, England, United Kingdom; 10 Diabetes Research Centre, College of Life Sciences, University of Leicester, Leicester, England, United Kingdom; 11 Therapy Department, University Hospitals of Leicester NHS Trust, Leicester, England, United Kingdom

**Keywords:** renal rehabilitation, digital inclusion, digital health intervention, Kidney Beam, exercise

## Abstract

**Background:**

The National Health Service 10-year health plan emphasizes an increasing shift toward digital health care delivery. However, there is limited research on how best to support, engage, and include individuals who are digitally excluded. As health care services become more digitally driven, evidence-based interventions are needed to address digital exclusion and ensure equitable access to care, particularly for people living with long-term conditions.

**Objective:**

This study aimed to evaluate the feasibility and acceptability of providing digital literacy training alongside a digital health intervention (DHI; Ex-Tab intervention), compared with providing a DHI alone. Kidney Beam, a DHI designed to promote physical activity and improve quality of life in people with chronic kidney disease (CKD), was used as an exemplar DHI.

**Methods:**

This mixed methods, single-site pilot randomized controlled trial recruited 40 adults with CKD who were digitally excluded. Digital exclusion was defined as lacking access to a Wi-Fi–enabled digital device or having a Digital Health Care Literacy Scale (DHLS) score of <7 (range 0‐21). Participants were randomized 1:1 to receive either the Kidney Beam Ex-Tab intervention or Kidney Beam alone (control). The intervention group received a Wi-Fi–enabled iPad on loan with Kidney Beam preinstalled, digital literacy training, and ongoing support to access the 12-week Kidney Beam program (twice weekly live exercise and education sessions). The control group received sign-up instructions for Kidney Beam only. Feasibility outcomes were assessed against a priori progression criteria and included screening, recruitment, retention, adherence, safety, and acceptability. Secondary outcomes included the Kidney Disease Quality of Life Questionnaire, Chalder Fatigue Questionnaire, and Patient Health Questionnaire-4. Outcomes were measured at baseline and 12 weeks. Acceptability and user experience were explored through semistructured interviews with participants from both groups at 12 weeks (n=25).

**Results:**

Between September 2023 and September 2024, a total of 169 individuals were screened and 40 were enrolled (median age 66.5 years; 20 male individuals; median DHLS score: 4). Twenty-one participants were randomized to the Kidney Beam Ex-Tab group and 19 to the Kidney Beam alone group. Of the 40 participants, 35 (88%) completed the 12-week follow-up (intervention: n=18; control: n=17). All prespecified feasibility criteria for recruitment, retention, adherence, and safety were met. Qualitative findings indicated that the tablet loan and digital literacy training were acceptable and highly valued, enhancing confidence, motivation, and DHI engagement. Providing loaned devices was particularly important for overcoming access barriers, especially for participants unable to afford their own device.

**Conclusions:**

Providing Wi-Fi–enabled devices and digital literacy training alongside a DHI was feasible and acceptable for people with lower digital literacy levels. The findings support progression to a future definitive multicenter trial or implementation study and offer transferable insights for the design of digital inclusion strategies for other long-term health conditions.

## Introduction

Digital health technology is the field of knowledge and practice associated with the development and use of digital technologies to improve health [1] and includes the utilization of smartphone apps, wearable devices, and platforms that provide remote health care [2]. A growing body of evidence indicates that digital health technologies provide significant benefits, including enhanced convenience, patient empowerment, improved self-management of chronic conditions, and notable environmental and economic efficiencies [3]. Furthermore, the UK Government’s 10-year health plan highlights the benefits of harnessing the digital revolution to create the world’s most digitally accessible health system [4], with the success of this vision relying on a shift toward digital delivery. Studies evaluating the use of digital health interventions (DHIs) have demonstrated feasibility, acceptability, clinical effectiveness, and cost-effectiveness across a wide range of long-term conditions, including type 2 diabetes [5], hypertension [6], chronic respiratory conditions [7], chronic kidney disease (CKD) [8], and cancer [9].

Kidney Beam [10] delivers online physical activity and self-management support and was co-designed with people living with CKD. A multicenter randomized controlled trial (RCT) showed that Kidney Beam is a clinically effective and cost-effective solution to improve health-related quality of life [8,11], confirming that DHIs can offer a scalable solution to deliver health care across people living with chronic conditions and can make interventions available for more people at a lower cost [12].

Despite the growing number of benefits, there are known barriers to accessing DHIs. These include a lack of physical resources; a lack of multilingual applications; low health and digital literacy; and socioeconomic limitations, including low household income, unstable employment, and housing insecurity [13]. If these limitations are not adequately addressed, they risk perpetuating the digital divide and further exacerbating existing health inequalities. The National Health Service (NHS) England has identified an urgent need to develop digitally enabled care pathways to increase inclusion [14]. This includes addressing the barriers to opportunity, access, knowledge, and skills for using technology [15]. The current evidence base is significantly underdeveloped in this area [16], and existing research has been primarily driven by the needs of health care providers, rather than the perspectives and priorities of underserved and digitally excluded service users.

Current evidence from the medical literature indicates that interventions to address digital health exclusion in health care are most effective when they are multifactorial and tailored to the needs of disadvantaged groups [17]. Interventions that combine efforts at the interpersonal, organizational, and policy levels, such as programs linking health care providers with other organizations and peer or family support, improve digital health literacy [18]. There is currently very limited RCT evidence specifically investigating interventions to improve digital inclusion among populations who are digitally excluded in health care. The existing body of literature is largely composed of scoping reviews, qualitative studies, and mixed-methods research, and notably, digital health literacy is absent from the development of all studies [19]. While these studies emphasize the importance of tailored, multiactor interventions, they provide limited evidence from robust RCTs targeting digitally excluded groups.

To address potential implementation barriers, this study aimed to evaluate the feasibility and acceptability of providing an iPad, digital literacy training, and support alongside a DHI (Ex-Tab intervention), compared with providing a DHI alone. The study used Kidney Beam as an exemplar DHI.

## Methods

### Ethical Considerations

The substudy was approved by the Bromley NHS Research Ethics Committee (reference number: 21/LO/0243) and Health Research Authority, and was preregistered on ClinicalTrials.gov (NCT04872933). Study information and consent materials were provided in accessible, nondigital formats, and researchers ensured that adequate time and opportunity were given to ask questions before consent was obtained. Participation was voluntary, and nonparticipation did not affect access to clinical care. Written informed consent was obtained from all participants before their involvement in the study (Multimedia Appendix 1). All collected data were deidentified to protect participant privacy and confidentiality. No compensation was provided to the participants.

### Study Design, Setting, and Participants

A feasibility RCT was conducted at a single site as a substudy of the Kidney Beam trial. The study protocol is presented in Multimedia Appendix 2. Adults aged ≥18 years with established CKD were recruited from King’s College Hospital in the United Kingdom, which serves a diverse urban population in South London. The inclusion and exclusion criteria for this study differed from those of the main study. The study included individuals lacking access to a Wi-Fi–enabled digital device or scoring <7 on the Vanderbilt University Medical Center Digital Health Care Literacy Scale (DHLS) [20,21]. The DHLS score ranges from 0 to 21, with higher scores indicating greater digital health literacy. In this study, a score of <7 was used to indicate reduced digital health literacy. Participants from the main study were not included in this substudy. The complete inclusion and exclusion criteria are provided in Table S1 in Multimedia Appendix 3. This manuscript is informed by relevant CONSORT (Consolidated Standards of Reporting Trials) extensions (Checklist 1) [22-24], the TiDieR (Template for Intervention Description and Replication) for intervention description [25], and the COREQ (Consolidated Criteria for Reporting Qualitative Research) for qualitative research (Checklist 2) [26].

### Recruitment

Potential participants were screened by the clinical team, and recent clinical records were reviewed to confirm eligibility at the time of enrollment. Suitable adults were approached in person during routine clinic visits, at outpatient physiotherapy appointments, or via telephone, by trained research staff. All participants provided written informed consent, which was obtained by the lead researcher (CW). Participants attended in-person assessments at baseline and 12 weeks at an NHS Health Centre. Some participants chose to complete their 12-week assessment via telephone. Outcome measures were completed online using SurveyMonkey, and support was offered by the researcher. Demographic and clinical characteristics were gathered from patient records (see Table S2 in Multimedia Appendix 3).

### Randomization

Participants were randomly assigned 1:1 to the Kidney Beam Ex-Tab group or the Kidney Beam alone group. Randomization was performed using a web-based system (Sealed Envelope [27]). Owing to the nature of the intervention, it was not possible to blind either the health care professionals delivering the intervention or the participants to group allocation.

### Kidney Beam Alone (Nonintervention) Group

Participants who were allocated to the Kidney Beam alone group were signed up on the Kidney Beam website and given a user guide on how to access the relevant exercise classes (Multimedia Appendix 4). They were instructed to exercise twice per week for 12 weeks.

### Kidney Beam Ex-Tab (Intervention) Group

The Kidney Beam Ex-Tab intervention was guided by patient and public involvement (PPI). Participants allocated to the Kidney Beam Ex-Tab group were provided with an iPad for 12 weeks, which had Kidney Beam preloaded. Moreover, they were provided with a Kidney Beam account and trained on using the iPad (Multimedia Appendix 5) and navigating the Kidney Beam website. This approach enabled a comparison of Kidney Beam usage between the 2 study groups.

The teaching session was delivered in person individually at an NHS Health Center by the research physiotherapist (CW) leading the study. It involved a demonstration, joint use of the iPad with feedback from the researcher, and autonomous participant use of the iPad. The teaching session was no longer than 60 minutes and was completed immediately after randomization at the baseline research visit. The teaching could not be individualized, and the instructional process remained consistent throughout the study period. Adherence to the intervention was monitored by recording the number of exercise classes completed, although fidelity of delivery was not formally assessed.

Participants were able to contact the research team and ask for assistance if they had any difficulties using the iPad from home, and in-person support was offered on request. Participants were instructed to use the iPad to exercise on the Kidney Beam website twice per week for 12 weeks. The iPads were configured with restricted access using device management settings, allowing participants to access only the Kidney Beam website via the preinstalled browser. No additional apps or websites were available. Participants were informed of this prior to providing consent. At the 12-week assessment, the iPad was returned to the research team. Features of the study intervention are presented in Textbox 1.

Textbox 1. **Kidney Beam Ex-Tab intervention summary.**

The intervention involves the following steps:

1. Account creation for the Kidney Beam (digital health intervention) website
2. Demonstration of how to use the basic functions of an iPad (turning on and off, and how to swipe, click, and charge the device)
3. Demonstration of how to navigate the Kidney Beam website to access relevant exercises (eg, seated or standing) and educational content
4. Participant trialing on how to use the iPad and navigate the Kidney Beam website with verbal support from the researcher
5. Independent iPad practice and use of the Kidney Beam website, without any verbal support from the researcher
6. Loaning of the device for 12 weeks to access Kidney Beam only (once the participant is fully independent, is satisfied with iPad use, and knows how to navigate Kidney Beam; Multimedia Appendix 6)

### Sample Size

Formal power calculations were not completed as recommended by the CONSORT guidelines for feasibility studies [22]. The target sample was 40 participants to allow assessment of recruitment, acceptability, and procedural issues, rather than statistical outcomes.

### Primary Outcomes for the Pilot Study

Primary feasibility outcomes included screening, recruitment, retention, randomization, outcome acceptability, intervention acceptability, and safety. Adverse events were monitored throughout the study and assessed by the research team for potential relatedness to the intervention or study procedures. For key feasibility outcomes, a priori criteria (Table 1) were used to establish progression to a definitive trial [28]. For each criterion, “stop” (indicating there were fundamental challenges that may impede a definitive trial) and “go” (indicating there were no challenges) thresholds were established. Results falling between these thresholds (“amber”) indicated that adaptations may render the definitive trial viable [28]. For secondary outcomes included within the pilot, refer to Table S3 in Multimedia Appendix 3.

**Table 1.** Progression criteria.

Outcome and status		Threshold
Recruitment (number of people recruited per month)		
Stop		<2 people recruited per month
Amber		2‐3 people recruited per month
Go		>3 people recruited per month
Intervention acceptability		
Stop		Engagement with less than 6 sessions
Amber		Engagement with 6‐11 sessions
Go		Engagement with at least 12 sessions
Outcome acceptability		
Stop		Less than 60% completion rate
Amber		61%‐79% completion rate
Go		At least 80% completion rate
Loss to follow-up		
Stop		More than 50% dropout rate during the pilot phase
Amber		21%‐49% dropout rate
Go		Less than 20% dropout rate

### Interviews

One-to-one, semistructured interviews were conducted with participants from the Kidney Beam Ex-Tab and Kidney Beam alone groups to explore their experiences of taking part in the trial and the experiences of the Kidney Beam Ex-Tab intervention. A purposive sample of participants from both groups was invited to complete individual semistructured interviews, either in person or via telephone. Interview participants were purposively sampled for maximum variation to ensure diversity across the sample. The sampling framework included ethnicity, age, sex, stage of CKD, history of renal replacement therapy (eg, dialysis), and intervention adherence level.

Topic guides (Table S5 in Multimedia Appendix 3) were developed in advance, and the PPI group reviewed and provided feedback prior to the first interview. The topic guides were broadly comparable for both groups, although questions relating to the intervention were excluded for the Kidney Beam alone group.

Consistent with the information power model [29], the sample size was not predetermined. Rather than aiming for theoretical saturation, which is associated with grounded theory, this study was guided by a framework analysis approach. We therefore sought to recruit a purposive sample of knowledgeable participants across both treatment groups, capturing a range of CKD stages and experiences of renal replacement therapy. Recruitment proceeded iteratively alongside analysis and ceased when the research team judged that the dataset provided sufficient information to address the study aim. This judgment was based on the focused research question, the specificity and relevance of the sample, and the depth and quality of the interview data. In later interviews, no substantially new insights were identified, and instead, these interviews elaborated and refined concepts already developed from earlier transcripts and did not add data that extended the existing analytical framework. Accordingly, data collection was concluded when additional interviews were assessed as unlikely to contribute meaningfully to the development of the analysis.

Interviews were conducted by experienced qualitative researchers who are renal physiotherapists working with people living with CKD (CW [male] and JB [female]). Participants were informed of the roles of the research team, with the lead researcher (CW) being responsible for assessments, randomization, and teaching, and the other researcher (JB) leading the Kidney Beam exercise classes. The researchers had no prior relationship with the participants before recruitment. All qualitative researchers kept a diary to enhance reflexivity and rigor [30]. Multiple researchers (CW, HMLY, and SF) coded the transcripts and ensured that the analysis and interpretation remained grounded in the data. Interviews were conducted as part of the 12-week reassessment either face-to-face or via telephone and were audio recorded.

The lead researcher (CW) held multiple roles in the study, being involved in randomization, delivering the teaching intervention, and conducting some of the qualitative interviews. This relationship may have influenced participants’ responses, for example, through social desirability or a perceived power dynamic. To mitigate this, participants were reminded that interviews were confidential, their responses would not affect their course participation or assessment, and honest feedback (both positive and negative) was valued. Interview questions were semistructured and neutrally phrased to minimize leading responses. The research team maintained reflexive diaries throughout the study to reflect on potential researcher influence during data collection and analysis, and interpretations were discussed among the wider research team to support reflexive awareness and analytical rigor.

### Data Analysis

Quantitative analysis was conducted by an independent statistician (JC) using SPSS (Version 29; IBM Corp). Descriptive statistics were used to estimate feasibility outcomes. Continuous variables that were not normally distributed are presented as median (IQR). Normally distributed continuous variables are presented as mean (SD). Categorical variables are presented as n (%). Secondary outcomes were considered exploratory; therefore, no *P* values are reported.

Qualitative interviews were independently transcribed verbatim. Data were managed using NVivo v15 (Lumivero) and Excel software (Microsoft Corp). Qualitative analysis was undertaken according to the framework approach [31] informed by the digital inclusion framework [32]. The framework identifies 5 domains where action is needed for digital inclusion: access to devices and data, accessibility and ease of use of technology, skills and capability, beliefs and trust, and leadership and partnerships. Transcripts were reviewed and coded line by line. Initial codes were discussed and refined, culminating in an analytical framework that was then systematically applied to the remaining transcripts. A matrix, which summarized the data from each participant by theme, was created.

## Results

### Feasibility Outcomes

#### Eligibility and Recruitment

**Figure 1.**
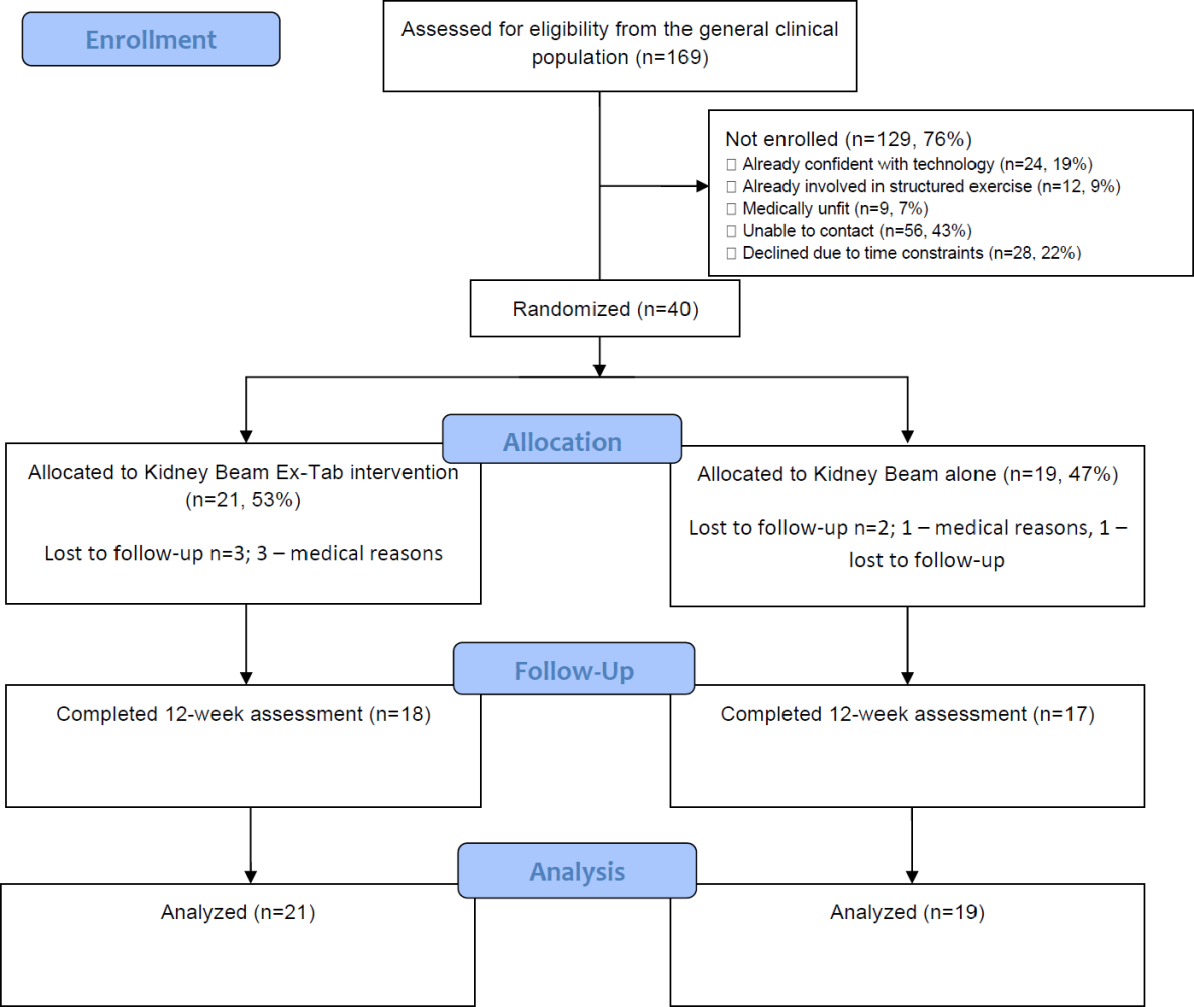
CONSORT (Consolidated Standards of Reporting Trials) flow diagram.

Screening and recruitment occurred from September 2023 to September 2024, with data collection completed by November 2024. Of the 169 adults screened, 129 were not enrolled. Figure 1 outlines the progression of participants through the pilot study.

**Figure 2.**
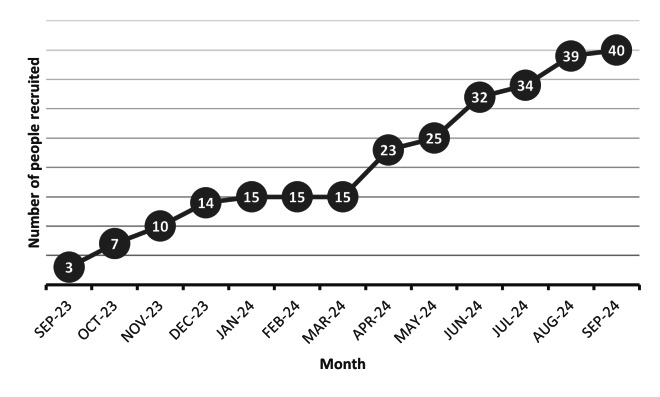
Actual rate of recruitment during the pilot trial (cumulative monthly recruitment).

Cumulative monthly recruitment rates are shown in Figure 2. The single site recruited a mean of 3 participants per month. Of those who consented, 40 progressed to randomization. Of the 40 participants randomized, 21 were allocated to the Kidney Beam Ex-Tab group and 19 were allocated to the Kidney Beam alone group. Recruitment stopped once the target of 40 participants was achieved.

#### Participant Characteristics

Overall, there were 20 (50%) male and 20 (50%) female participants, and the majority of participants were Black adults (19/40, 48%). At baseline, the Kidney Beam Ex-Tab group had older participants and more female participants compared with the Kidney Beam alone group. Other factors were well matched between the groups (Table S2 in Multimedia Appendix 3). Digital health literacy scores were similar in both groups.

#### Retention

Of the 40 participants, 5 (13%) were lost to follow-up, including 3 (8%) from the Kidney Beam Ex-Tab group and 2 (5%) from the Kidney Beam alone group. The reasons for withdrawal included becoming medically unfit (n=4) and being lost to follow-up (n=1).

#### Exercise Adherence

**Figure 3.**
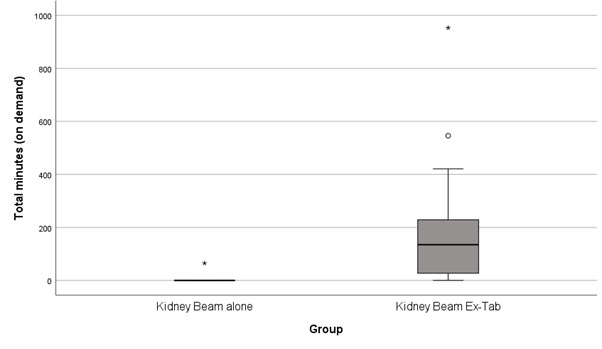
Time spent (minutes) on completing classes on Kidney Beam in the Kidney Beam alone and Kidney Beam Ex-Tab groups. The asterisk denotes an 'extreme outlier' in a box and whisker plot. Extreme outliers: Values that are more than 3.0 x IQR below Q1 or above Q3 are represented by asterisks.

Participants in the Kidney Beam Ex-Tab group completed more exercise sessions and spent more time on the platform compared with the Kidney Beam alone group (Table 2). In the Kidney Beam Ex-Tab group, participants performed a median of 9 (IQR 2‐18) exercise sessions, with 12 participants performing at least two sessions (2‐43 completed classes). In the Kidney Beam alone group, only 2 participants completed any classes (one participant completed 2 classes, and the other completed 1 class), and the remaining participants did not complete any classes. The time spent on exercise classes in the 2 groups is presented in Figure 3.

**Table 2.** Exercise adherence levels by the number of exercise sessions in the Kidney Beam Ex-Tab and Kidney Beam alone groups.

Variable	Kidney Beam Ex-Tab group (n=21), n (%)	Kidney Beam alone group (n=19), n (%)
Engagement with less than 6 sessions	9 (43)	19 (100)
Engagement with 6‐11 sessions	4 (19)	0 (0)
Engagement with at least 12 sessions	8 (38)	0 (0)

#### Outcome Acceptability

Overall, 88%‐100% of outcome data were completed at baseline, and 80%‐85% were completed at 12 weeks (Table 3). A total of 5 participants completed the assessment by telephone rather than attending in-person evaluations (2 in the Kidney Beam Ex-Tab group and 3 in the Kidney Beam alone group).

**Table 3.** Secondary outcome measure completion rates at baseline and 12 weeks.

Measure	Completed at baseline, n (%)	Completed at 12 weeks, n (%)
Sit-to-stand test (60 s)	40 (100)	32 (80)
PHQ-4^a^	38 (95)	34 (85)
Chandler Fatigue Scale	35 (88)	34 (85)
KDQoL-SF1.3^b^	39 (98)	34 (85)

a PHQ-4: Patient Health Questionnaire-4.

b KDQoL-SF1.3: Kidney Disease Quality of Life-Short Form version 1.3.

#### Safety

Three serious adverse events occurred during the study period, including 1 cardiac arrest in the Kidney Beam alone group and 2 deaths in the Kidney Beam Ex-Tab group (1 following emergency surgery and 1 of unknown cause). The 2 participants in the Kidney Beam Ex-Tab group had not undertaken any Kidney Beam exercise classes. Based on the clinical information available to the research team, none of these events were considered to be related to participation in the study or the intervention.

### Secondary Quantitative Outcome Measures

Secondary quantitative outcome data are summarized in Table 4.

**Table 4.** Secondary outcome measure results.

Measure	Kidney Beam Ex-Tab group			Kidney Beam alone group		
	Baseline	12 weeks	Difference	Baseline	12 weeks	Difference
Kidney Beam exercise time (min), median (range)	—^a^	135.0 (13.5‐244.5)	—	—	0 (0-0)	—
KDQoL-SF1.3^b^ MCS^c^, mean (SD)	49.20 (11.9)	51.25 (8.03)	+2.05	50.46 (11.24)	50.51 (12.38)	+0.05
KDQoL-SF1.3 PCS^d^, mean (SD)	32.43 (10.07)	37.99 (11.49)	+5.56	37.28 (10.75)	33.18 (11.93)	−4.10
Sit-to-stand test (60 s) repetitions, mean (SD)	13.67 (5.38)	16.87 (5.38)	+3.20	17.53 (7.78)	13.06 (11.38)	−4.47
PHQ-4^e^ score, median (range)	3.00 (0.00‐7.00)	0.00 (0.00‐3.00)	−3.00	3.00 (0.00‐6.00)	1.00 (0.00‐7.00)	−2.00
Chandler Fatigue Scale score, median (range)	14.00 (11.75‐15.25)	11.00 (11.00‐20.50)	−3.00	13.00 (12.00‐20.00)	15.00 (12.00‐22.00)	+2.00

a Not applicable.

b KDQoL-SF1.3: Kidney Disease Quality of Life-Short Form version 1.3.

c MCS: mental component summary.

d PCS: physical component summary.

e PHQ-4: Patient Health Questionnaire-4.

### Qualitative Findings

#### Overview

From both groups, 27 participants were approached, and of these, 25 agreed to complete qualitative interviews. Two declined owing to not wanting to have their voice recorded. Baseline characteristics of the 25 participants are summarized in Table 5. Interviews were conducted between March 19, 2024, and November 27, 2024. Participants were interviewed either by telephone at their homes or in person at an NHS medical center, without the presence of nonparticipants.

**Table 5.** Demographic and clinical characteristics of the qualitative participants.

Variable		Value (N=25)
Age (years), median (range)		67.92 (59-74)
Sex, n (%)		
Female		11 (44)
Male		14 (56)
Ethnicity, n (%)		
White		8 (32)
Black		14 (56)
Asian		3 (12)
Treatment group, n (%)		
Kidney Beam Ex-Tab (intervention)		13 (52)
Kidney Beam alone (control)		12 (48)
Treatment modality, n (%)		
Non–dialysis-dependent kidney disease		9 (36)
Kidney transplant recipient		4 (16)
Dialysis therapy		12 (48)
Exercise sessions completed, n (%)		
Less than 6 sessions		15 (60)
6‐11 sessions		3 (12)
At least 12 sessions		7 (28)
Length of interviews (min), median (range)		23 (11-43)

The results are outlined in 5 themes based on the NHS digital inclusion framework: access to devices and data, accessibility and ease of use of technology, skills and capability, beliefs and trust, and leadership and partnerships.

#### Access to Devices and Data

##### Digital Inequities

Participants described limited digital literacy, low motivation to engage with technology, and barriers to device maintenance and connectivity. Participants encountered difficulties with basic mobile phone functions like answering the phone and sending SMS text messages. Limited understanding of key digital concepts, such as Wi-Fi and mobile data, further compounded these challenges. Although some participants had access to Wi-Fi at home, many lacked the knowledge to effectively use it or connect their devices. Minimal use of digital communication beyond essential functions was common, restricting patients’ interactions with digital health platforms, and some participants showed little interest in enhancing their digital skills, which constrained their use of health-related technologies. Participants also faced issues with internet connectivity, and unreliable signals and buffering frequently disrupted their online activities, causing frustration.

*I don’t know which button to push to answer the damn thing… I don’t know how to answer the phone.*

[Male, 75 years old, kidney transplant recipient, White British]

*When the internet keeps buffering and things like that. I think it’s more losing the signal and you get frustrated because you’re in the middle of doing something.*

[Female, 68 years old, kidney transplant recipient, White British]

##### Financial Strain

Financial strain was a significant barrier to accessing and maintaining digital devices essential for health engagement. Affordability influenced the initial acquisition of technology and decisions around repair and replacement. Other financial priorities often took precedence over purchasing or repairing a digital device, while reliance on others or government benefits limited the ability to invest in better devices. Frequent technology issues and costly or failed repairs added to the challenge, sometimes requiring new purchases. Limited knowledge of maintenance worsened the problem, creating major barriers to using digital devices effectively. Community support and donations were seen as potential solutions to ease these financial burdens.

*To buy it, you’ve got to know what you want to use it for. At the moment, that’s one of the reasons. The other… is finance. At the moment I’m going through decorating my place. And also I’ve got other things to deal with, like I’ve got to sort a headstone for my mother. And they’re taking priority in that sense.*

[Male, non–dialysis-dependent kidney disease, 67 years old, Black Caribbean]

##### Role of Social Support

Many participants emphasized the importance of social support and cohabitation in facilitating their access to and use of digital technologies. Those living with family or partners often relied on others’ devices.

*I think I don’t really need it because when she [participants daughter] comes, I always use her [participants daughters] phone.*

[Male, dialysis-dependent kidney disease, 60 years old, Black African]

*I haven’t really got much help from anybody and I live on my own… Because I’m in one of those situations where I’m on my own. Everything I have to do on my own.*

[Male, dialysis-dependent kidney disease, 73 years old, White British]

*That kind of put me back a bit when I haven't got no-one in the house… so that means I don’t learn as fast.*

[Female, dialysis-dependent kidney disease, 74 years old, Black Caribbean]

### Access to Devices and Data (Intervention Acceptability)

Participants in the Kidney Beam Ex-Tab group generally regarded the loaning of tablets and associated training as a valuable and acceptable aspect of the intervention, which facilitated engagement and motivation in completing exercises. The loaning of devices was widely welcomed as a means to overcome the challenges of having access to a device, especially for those unable to afford their own device.

Other positives from having access to an iPad included participants valuing the opportunity to develop digital skills and increase their confidence and willingness to use digital technology following the completion of the study. Many participants frequently expressed concerns about responsibility for the loaned devices. Some participants feared potential damage or loss and worried about the financial repercussions of this, and thus, they adopted protective behaviors to avoid damage. Overall, participants recognized that borrowing devices demanded responsibility but considered this an acceptable tradeoff for increased access to digital devices like iPads.

*Not everybody can afford to buy one… And it gets you active again. Helps you get active again to get on that program.*

[Female, kidney transplant recipient, 68 years old, White British]

*It’s a good idea to those that need it and does not have it and would like it and that would help them, of course it’s a good idea… Because it will help them isn't it, whatever the need is, because sometimes you have a need and you haven't got the equipment, whatever, for that need, but when you have the equipment it will help.*

[Female, dialysis-dependent kidney disease, 74 years old, Black Caribbean]

*It’s OK to borrow things on the NHS but I’d be terrified of losing things! Or breaking things!… Nobody likes to break something that’s not theirs and one would feel a bit guilty! Because everything costs money… So yes, I think it’s a good idea to be that people are able to reach out and get things, it’s just for me when it breaks I don’t like it!*

[Female, non–dialysis-dependent kidney disease, 69 years old, Black Caribbean]

### Accessibility and Ease of Use of Technology

Participants struggled to navigate the iPad, the internet, and Kidney Beam due to low digital confidence and unfamiliarity. They found it difficult to move between pages, locate exercises, and manage passwords. Using Kidney Beam on mobile devices was especially challenging because of unclear website navigation, small screens, and limited awareness of options like casting to a television screen. Password management added further barriers, with participants frequently forgetting details and having concerns about storing the details securely. These issues often led to inconsistent use and reliance on family support.

*Sometimes I get into it and then something will happen and it sort of disappears and I don’t know how to get back to it and it’s a lot of fiddling and faddling and I'm calling my son saying ‘help me get back on this’, or whatever…*

[Male, dialysis-dependent kidney disease, 59 years old, Black Caribbean]

*The negative is the screen is so small – I know it’s a big iPad, but then you’re trying to exercise. You’re like not in front of it as such.*

[Female, dialysis-dependent kidney disease, 58 years old, Black Caribbean]

*Everybody seems to want passwords and codes and all the rest of it. The more I get, the more confused I get and I don’t necessarily remember them all.*

[Male, dialysis-dependent kidney disease, 77 years old, White British]

Participants also reported a range of physical challenges that impeded their ability to use digital devices. Visual impairments, including severe sight loss and deteriorating eyesight, made it difficult for some to see and interact with screens effectively. In addition to vision problems, physical health conditions, such as arthritis and back pain, affected participants’ ability to handle devices. Pain, stiffness, and limited dexterity in the hands often resulted in difficulties tapping screens accurately or sustaining interaction with the technology. These physical limitations not only reduced usability but also contributed to feelings of frustration and decreased motivation to engage.

*I didn’t really use technology at all, because I’m registered severely sight-impaired. So I can’t see screens. My phone, I ask it to get me a telephone number and I ask it to read me a text message. But other than that, I wish I could see it.*

[Female, non–dialysis-dependent kidney disease, 78 years old, White British]

*But at the moment, honestly, to tell you the truth, I don’t feel good at all. My health, my arthritis is affecting me.*

[Female, non–dialysis-dependent kidney disease, 80 years old, Asian]

### Skills and Capability

Many participants described having limited or basic digital skills and abilities. Several participants reported struggling with fundamental digital functions, such as typing, or using software and mobile devices. A minority of participants reported moderate or advanced digital skills, often linked to previous work experience; however, this did not always translate into regular or confident use.

*I don’t know much about the clouds and all that… nobody has taught me about it.*

[Female, dialysis-dependent kidney disease, 57 years old, Black African]

*I can search for some things but… I’m not always confident of when you get into these sites.*

[Male, dialysis-dependent kidney disease, 59 years old, Black Caribbean]

*Well I’m reasonably confident with the things I know how to do. But anything more complicated I struggle with.*

[Male, dialysis-dependent kidney disease, 77 years old, White British]

Many participants reported difficulties with memory, attention, and cognitive processing. These challenges were described as both persistent and frustrating and were linked to aging, neurological conditions, or general cognitive decline.

*After the stroke, I tell you, everything left my head. Everything is like… a blank space.*

[Male, dialysis-dependent kidney disease, 55 years old, Black African]

*I suppose actually remembering the different steps to take to get the computer working…*

[Male, kidney transplant recipient, 72 years old, Asian]

*It can come now today in my head and tomorrow I can forget it.*

[Male, dialysis-dependent kidney disease, 57 years old, Asian]

### Skills and Capability (Intervention Acceptability)

Participants emphasized the importance of guided support. Instructions and practice were included in the intervention, and participants were able to develop the skills and confidence to use the iPad effectively. Several participants highlighted the need for clear, hands-on teaching or being shown step-by-step instructions. Learning to use the iPad by repetition was helpful. Participants found the initial teaching as part of this study helpful, noting that it boosted their confidence. Many participants described improvements and the practical benefits of using the iPad over time.

*You explained it properly to me, you know, I still didn’t quite get it straightaway but eventually I got it as spoke to each other then I got used to it, you explained it properly to me and showed me how to use it, so I was fine with that.. you explained it properly to me, I didn’t have no problem with understanding you or you telling me what to do.*

[Female, non–dialysis-dependent kidney disease, 65 years old, Black Caribbean]

*My confidence is more, should I say, I have more confidence than before…Because you know when you just start something, you say oh I don’t think I can do this for, what, for three months and something, yeah, I've got confident now, yeah.*

[Female, dialysis-dependent kidney disease, 64 years old, Black African]

### Beliefs and Trust

Participants described ambivalence toward technology, which was strongly influenced by generational factors and shaped by individual beliefs and trust concerns. Many older adults expressed resistance to adopting digital technologies, describing themselves as “not of the computer age” (male, hemodialysis, 77 years old, White British) or being “a bit of a dinosaur” (male, posttransplant, 75 years old, White British), highlighting a generational divide in familiarity and comfort with technology. The perception that technology is “too much” (female, stage 2, 69 years old, Black Caribbean) or “too complicated” (male, hemodialysis, 77 years old, White British) led some participants to disengage entirely from digital services. This resistance was frequently attributed to perceived complexity and lack of personal relevance, with participants emphasizing a preference for traditional, interpersonal modes of communication and transactions over digital interfaces.

*I’m just not really of the computer age… it’s just not something I grew up with.*

[Male, dialysis-dependent kidney disease, 77 years old, White British]

*It’s not personal, you know? I like talking to someone face-to-face, not just buttons and screens.*

[Male, dialysis-dependent kidney disease, 59 years old, Black Caribbean]

There was widespread apprehension regarding security and privacy, with participants voicing fears of fraud, scams, and hacking. Several participants had been targeted by scammers, which significantly undermined their trust in digital platforms.

*I'm afraid I press the button and give away the little money that I've got or something! Or let somebody else in my account or whatever. I'm just afraid of it.*

[Female, non–dialysis-dependent kidney disease, 69 years old, Black Caribbean]

*I’m very, very aware of scammers and that. So I don’t have a mainline telephone any more. I used to get odd calls on that. But I’m very, very aware of what scammers do. So maybe that’s an advantage in a way that I don’t use it, because I can’t be scammed.*

[Female, kidney transplant recipient, 57 years old, Black African]

Despite concerns, some participants acknowledged the inevitability of adapting to technology, often framed as a reluctant acceptance driven by practical necessity rather than enthusiasm. Some participants demonstrated cautious trust, stating that they would use digital health apps “if they trusted it” (male, stage 3a, 67 years old, Black Caribbean).

*I mean you’ve got digital banking coming on now right, you know? They move away all the banks, so they turn round telling you “Oh, do it online.” So you slowly move towards that direction… If I didn’t trust it, I wouldn’t use it, you know. I think you’ve just got to be, keep an open mind about it. Stuff that is too dodgy your bank will stop it, or if it does go through, you can get back on it.*

[Male, non–dialysis-dependent kidney disease, 67 years old, Black Caribbean]

*If I didn’t trust it, I wouldn’t use it, you know. I think you’ve just got to be, keep an open mind about it [about using Kidney Beam in the future]*

[Male, dialysis-dependent kidney disease, 59 years old, Black Caribbean]

### Beliefs and Trust (Intervention Acceptability)

Participants continued to have mixed beliefs about technology and digital interventions even after completing the study, which were influenced by personal confidence, perceived usefulness, and trust in their ability to engage. Some participants recognized the growing relevance of digital tools in business and other areas and would consider using them in the future. Participants appeared to hold the view that borrowing resources from the NHS was permissible, given its status as a trusted and authoritative organization. Many participants who used Kidney Beam would continue to use it if support remained and it was convenient. Overall, attitudes reflect a balance of hesitation and openness, with familiarity, necessity, and perceived benefit playing key roles in shaping future iPad and Kidney Beam engagement.

*The way it’s going, it’s the way things are going to be going anyway. So it’s already going to that way. But for me personally, yeah, I mean it’s going to impact on everybody, but again, I don’t see me delving into the heights of technology to be honest.*

[Male, dialysis-dependent kidney disease, 59 years old, Black Caribbean]

*I don’t think I have the mind to use the, to follow the technology that’s coming into the system. With my set of mind and my mind, and my level of education I’ve had and gone through, and I’m really, really scared to use these new, these technologies, these computers, these laptops.*

[Male, non–dialysis-dependent kidney disease, 83 years old, Black African]

*If, like I say, if it could go on my little tablet and I was shown how to do, like I was shown how to do that one, then I could most probably carry on doing it. [talking about continuing with Kidney Beam in the future]*

[Male, kidney transplant recipient, 75 years old, White British]

### Leadership and Partnerships

Participants emphasized the need for stronger leadership and clearer support structures with digital technologies. Many participants expressed a desire for more frequent check-ins, reminders, and personalized guidance to stay engaged and overcome challenges. Where formal support was lacking, some turned to family members or peers, but this was not an option for everyone. Participants also highlighted the value of one-to-one interactions and suggested that accessible digital devices or remote support could improve autonomy and confidence. Participants suggested strengthening partnerships with in-center dialysis units and libraries to offer support and leadership with engagement for digital technologies.

*Well, my daughter and that sort of thing that can help me you know. But she’s not here all the time. That’s the problem… They are too busy in their own things. So you know nowadays children don’t have time for you… That’s what I feel. I don’t bother them. I can’t rely on them.*

[Female, non–dialysis-dependent kidney disease, 80 years old, Asian]

*Well probably, just probably more contact, right? More reminders or one to one ongoing talk.*

[Male, non–dialysis-dependent kidney disease, 67 years old, Black Caribbean]

*In sense of people come to dialysis centres, communicate with them, talk to them, educate them about this and what we are trying to do, this is how to achieve the aim [getting more people exercising online].*

[Female, dialysis-dependent kidney disease, 57 years old, Black African]

### Leadership and Partnerships (Intervention Acceptability)

Participants highlighted the importance of the intervention package (encouragement, support, and access) used in this study in shaping its acceptability and potential effectiveness. Many participants suggested that other digitally excluded people would also benefit from this intervention and that it should be supported by leaders and organizations to achieve wider reach. Several individuals stressed the value of flexibility and autonomy enabled by borrowing an iPad and having access to Kidney Beam, reinforcing the need for supportive systems that allow users to engage on their own terms. Participants also acknowledged the role of guidance and reminders from the research team, suggesting that effective leadership in the form of clear instructions or check-ins can enhance engagement. Some participants advocated for peer encouragement and shared responsibility. Overall, these reflections underline the importance of partnerships among patients, health providers, and digital health leaders, where encouragement, accessible resources, and sustained support foster engagement.

*It could help a lot of people in lots of different ways…well it helped me I think… but I think on the whole it is a good idea.*

[Male, kidney transplant recipient, 75 years old, White British]

*I got a lot of encouragement from the young guy and especially that young lady. She was marvellous.. I’m never going to be the ‘Green Goddess’ or nothing like that but yeah, it suited me, because I was able to do it in my own time. But you still need somebody there at least once in the week to encourage you. And then you think about that, and that gives you an incentive to do it yourself. So you’re not reliant on that person all the time, but it gives you an incentive to carry on doing it.*

[Female, non–dialysis-dependent kidney disease, 78 years old, White British]

*Well I would say to other patients go for it. It’s worthwhile, yeah…I feel fitter. Happier. Able to do more… Well when I get my new computer I’ll do it on my computer or I might even invest in getting myself an iPad.*

[Female, kidney transplant recipient, 68 years old, White British]

### Mixed Methods Synthesis

A convergent mixed methods analysis was used, in which quantitative and qualitative data were collected and analyzed separately but concurrently before being integrated within a joint display (Table 6). This joint display brings together the quantitative feasibility outcomes and qualitative interview findings to identify adaptations for a future definitive trial, highlighting how qualitative insights help explain the quantitative engagement patterns and inform refinements to the intervention and study design.

**Table 6.** Joint display of quantitative and qualitative findings with integrated interpretation and implications for a future definitive trial.

Feasibility domain	Progression criteria	Quantitative findings	Qualitative findings	Mixed methods interpretation	Adaptations required for a definitive trial
Recruitment	Amber: 2‐3 people recruited per month	An average of 3 participants consented per month	Not explored in qualitative interviews	Recruitment targets were met but were modest, suggesting that recruitment was feasible but may have been limited by the single-site design, rather than participant acceptability of the intervention.	Quantitative: Open recruitment to other sites in the United Kingdom.
Intervention acceptability	Amber: Engagement with 6‐11 sessions	Mean of 11 exercise classes completed	Participants reported increased access to Kidney Beam, development of new digital skills, and improved confidence using digital technology. The one-to-one teaching and support were perceived as helpful and acceptable.	Qualitative findings help explain the relatively high engagement observed in the quantitative data. Participants indicated that provision of a device, training, and support enabled them to use the platform confidently, suggesting that these elements facilitated sustained engagement with the intervention.	Quantitative: Monitor session completion and engagement to optimize intervention intensity. A digital inclusion exercise program should be created; Qualitative: Reduce password and login burden, create individual shortcuts on the iPad, provide remote support, and refine the teaching process to improve usability.
Outcome acceptability (for all outcome measures)	Go: At least 80% outcome completion rate	80%‐85% outcome completion rate	Not explored in qualitative interviews	High completion rates suggest that the outcome measures were acceptable and feasible to administer in this population.	Quantitative: Validate outcome measures for assessing digital literacy and for use at baseline and 12-week assessments.
Loss to follow-up	Go: Less than 20% dropout rate	12.5% dropout rate	Not explored in qualitative interviews	The low dropout rate indicates good participant retention and overall acceptability of the study procedures.	Quantitative: Maintain follow-up procedures; Qualitative: Clear identification of liability and responsibility for the iPad.
Study design	—^a^	—	—	Considerations arising from feasibility findings suggest that a more robust design may be required to strengthen internal validity in a future trial.	Consider an attention-matched control or stepped-wedge design to improve internal validity.
Fidelity of intervention delivery	—	—	—	Ensuring consistent delivery of the intervention will be important in a larger trial.	Include fidelity monitoring using checklists or session recordings to ensure the intervention is delivered as intended.

a Not applicable.

## Discussion

### Principal Findings

This mixed methods pilot study evaluated the feasibility, acceptability, and design of a future RCT for a novel digital inclusion intervention designed for individuals who are digitally excluded from DHIs. The study achieved its predefined progression criteria for outcome acceptability and loss to follow-up, and adverse events were unrelated to the intervention.

There is a limited body of published literature examining interventions specifically designed to address digital exclusion within health care settings. Consequently, limited prior evidence regarding expected recruitment rates for digital inclusion studies further complicated planning, as this remains an emerging area of research [33]. The recruitment target of 40 participants was achieved over 12 months, with an average of 3 participants consenting each month. Although recruitment was slower than initially anticipated, this aligns with well-documented challenges in engaging people living with CKD in research [34], including limited understanding of research participation, competing health and personal priorities, and lower literacy levels, particularly within dialysis populations [35,36]. Additionally, more people than expected did not meet the inclusion criteria, with screened participants being of higher digital literacy levels and/or having access to Wi-Fi–enabled technology.

In the main Kidney Beam trial [11], 19 participants (1.7%) assessed for eligibility reported having no access to a suitable device or Wi-Fi connection. This figure is likely underestimated, as digital access was self-reported by participants and is more difficult to verify than medical exclusion criteria, which can be confirmed through clinical records. Furthermore, neither the main study nor previous studies routinely screened for digital literacy; therefore, the digital screening process implemented in this pilot was newly developed.

Baseline demographic characteristics were broadly comparable between groups in this study. However, there were some clinical differences, with a greater proportion of non–dialysis-dependent participants in the Kidney Beam alone group and more kidney transplant recipients in the Kidney Beam Ex-Tab group. In a small sample, baseline imbalances may occur by chance but could influence engagement or retention, as age, sex, and modality can affect health behaviors and participation in digital or exercise interventions. As a pilot study, these differences should be interpreted cautiously and addressed in future larger trials.

Outcome data completion rates were high at baseline (88%‐100%) and at 12 weeks (80%‐85%). The Kidney Beam pilot study [37] by comparison had 90%‐100% data completeness at baseline and 62%‐83% at 12 weeks, where assessments were delivered virtually. The high retention rate (88%) could be attributable to face-to-face study visits and the requirement to return loaned iPads in person, which helped maintain contact and engagement. The Kidney Beam pilot study [37] by comparison reported a retention rate of 83.4% and subsequently progressed to a successful, fully powered RCT.

No safety events or deaths were related to study participation, and all iPads were returned undamaged, suggesting that the device loan system was manageable and acceptable to participants and families. Implementation required the provision of iPad devices and mobile data connectivity. Ten iPads were purchased at £525 (US $696) each (£5250 [US $6964] total), and SIM cards were provided at £5 (US $6.6) per month. Twenty-one participants used the devices during the study. When distributed across participants, the estimated hardware cost was approximately £250 (US $331) per participant, with an additional £5 (US $6.6) per participant per month for data. Future costs may be reduced through device reuse, participant-owned devices, or institutional Wi-Fi access.

The lower time spent on the platform by intervention participants in this pilot study compared with the main study [11] may reflect differences in both program allocation and participant characteristics. In the main study [11], participants were signposted to a specific exercise program, whereas in this pilot study, some participants completed seated-only exercise sessions due to reduced mobility and balance. These sessions are shorter in duration, which likely contributed to the lower overall engagement time observed. Additionally, participants in the pilot study were older than those in the main trial (mean age 66.5 vs 53.8 years) [38], and the sample included a substantially higher proportion of participants from Black ethnic backgrounds (47.5% vs 11.5%). Comorbid conditions were also more prevalent among participants from the pilot study than among participants from the main study (diabetes: 60.0% vs 22.4%; hypertension: 92.5% vs 69.1%) [11]. The lower cumulative engagement observed in this study may still represent an appropriate intervention “dose” for individuals with greater functional limitations, particularly where shorter and more accessible sessions support participation that might otherwise not occur. These differences suggest that a digital inclusion exercise program tailored to the needs of this population group may be required, as their needs appear to differ from those of participants in the main study [11,38].

Further work is needed to increase time spent on the platform to optimize potential health benefits, as demonstrated in the main study [11]. The benefits of exercise can still be achieved through sporadic, accumulated activity [39]; therefore, this older, more comorbid population may benefit from interventions lasting longer than 12 weeks to increase overall time spent exercising. Greater collaboration with communities is also required to better understand and address participant preferences and needs across organizations, thereby improving practice and supporting wider implementation across services [15,40]. The difference in the time spent on the platform between the main study [11] and this study may be explained by variations in program allocation. Participants in the main study [11] were signposted to a specific exercise program, whereas some participants in this pilot study completed seated-only exercise programs due to reduced mobility and balance. These seated sessions were shorter in duration than the exercise program used in the main study [11], which likely contributed to the lower total engagement time observed. The qualitative findings identified consistent barriers, including password and login burden, low digital confidence, physical and memory limitations, and fear of damaging the device. These issues indicate that the intervention requires further refinement to simplify access and improve support before a larger trial.

The teaching component of the intervention was highly valued. Participants emphasized the importance of step-by-step instructions, opportunities for repeated practice, and ongoing support. The intervention appeared to enhance participants’ confidence in navigating digital devices, including basic functions such as logging in, scrolling, and online navigation. Digital literacy was assessed only at baseline as part of the inclusion criteria; however, future studies should consider measuring changes in digital literacy over time to quantitatively capture improvements resulting from the intervention.

This study contributes to the growing literature suggesting that addressing digital health exclusion requires multifactorial and tailored approaches. Consistent with previous studies highlighting the importance of interventions spanning interpersonal and organizational levels [17,18], the provision of devices alongside digital literacy training and ongoing support was found to be feasible and acceptable among participants. The existing evidence base for digital inclusion interventions in health care has been limited by a lack of RCTs and a predominance of qualitative and mixed methods research [19]. While the study was not designed to assess effectiveness, it addresses an important gap by demonstrating the feasibility of embedding digital access and literacy support within a DHI.

This digital inclusion intervention was overall deemed acceptable from the qualitative findings, as access to an iPad and tailored digital training were key enablers of engagement. Participants described the intervention as both a practical solution to digital exclusion and a gateway to developing new digital skills. However, some participants expressed anxiety regarding the responsibility for loaned devices and the potential for device damage. This is an important lesson for future digital health inclusion studies, including those involving other long-term health conditions. Future trials should therefore include clear communication protocols and reassurances about device liability to reduce participant concern.

### Limitations

This study was conducted at a single site in London, United Kingdom, where the study sample included a relatively high proportion of Black participants (48%), which differs from national CKD demographics in the United Kingdom. As a result, the findings may not be fully generalizable to other regions of the United Kingdom with different demographic profiles. Digital exclusion may present differently in rural or less ethnically diverse settings, where barriers may relate more to infrastructure limitations, internet connectivity, or geographic isolation, rather than language, socioeconomic inequalities, or varying levels of digital literacy. Future studies should therefore consider multisite recruitment across diverse geographic and demographic contexts to enhance generalizability.

Qualitative interviews focused on exploring barriers to digital health engagement and the perceived impact of the intervention. Broader study procedures, such as recruitment, data collection, and randomization processes, were not explored qualitatively, and feedback on the study procedures would have helped inform future studies.

Participants in the Kidney Beam alone group were not offered the opportunity to borrow an iPad with Kidney Beam access. The control condition reflected current usual care, where individuals without access to a suitable device or the necessary digital skills may be unable to engage with DHIs. Future definitive trials may benefit from alternative comparator designs, such as attention-matched controls or stepped-wedge designs, to provide a more balanced comparison.

The fidelity of delivering the teaching component was not formally evaluated in this study. As these onboarding sessions were conducted over a 12-month recruitment period, some variation in delivery may have occurred. Future definitive trials should incorporate fidelity monitoring strategies, such as structured training protocols, checklists, and session recording, to ensure standardized delivery and to support scalability across multiple instructors and sites.

Digital literacy was assessed at baseline using the DHLS to screen for participants with lower levels of digital literacy (score <7). As this pilot study primarily aimed to examine recruitment, acceptability, and engagement with the intervention, follow-up assessment of digital literacy was not included. However, it is recognized that changes in digital literacy represent an important outcome for digital inclusion interventions. Future definitive trials should therefore incorporate follow-up DHLS assessments to evaluate changes in digital literacy over time.

This study delivered a complex intervention that included providing a loaned device, preloaded access, setup support, training, and ongoing assistance. Because these elements were delivered together, it is not possible to determine the relative contribution of each component to the observed effects. Qualitative interviews provided insights into potential mechanisms underlying the observed changes; however, future studies could examine the individual contribution of these components to better understand how the intervention influences engagement.

### Conclusion

The findings of this pilot study support progression to a definitive trial to robustly assess the intervention’s effectiveness in addressing digital exclusion. The intervention was considered to be acceptable, although qualitative feedback identified areas for refinement, particularly in improving usability and providing clearer guidance around responsibility for the loaned iPads. Future research should focus on refining and personalizing the intervention to better meet individual needs, as the causes of digital exclusion are diverse. Moreover, this approach holds promise for adaptation and evaluation in other long-term conditions, offering a potential pathway to reduce digital exclusion and improve equitable access to digital health resources.

## Supplementary material

10.2196/91438 Multimedia Appendix 1 Informed consent form.

10.2196/91438 Multimedia Appendix 2 Study protocol.

10.2196/91438 Multimedia Appendix 3 Data to support the study.

10.2196/91438 Multimedia Appendix 4 Kidney Beam website screenshots.

10.2196/91438 Multimedia Appendix 5 iPad user guide.

10.2196/91438 Multimedia Appendix 6 iPad loan agreement document.

10.2196/91438 Checklist 1 CONSORT checklist.

10.2196/91438 Checklist 2 COREQ checklist.

## Abbreviations

CKD: chronic kidney disease
CONSORT: Consolidated Standards of Reporting Trials
COREQ: Consolidated Criteria for Reporting Qualitative Research
DHI: digital health intervention
DHLS: Digital Health Care Literacy Scale
NHS: National Health Service
PPI: patient and public involvement
RCT: randomized controlled trial
TiDieR: Template for Intervention Description and Replication
