# Supplementary material for: A Digital Inclusion Intervention to Improve Access to a Digital Health Intervention Among Digitally Excluded Adults: Mixed Methods Pilot Randomized Controlled Trial
Source: JMIR Form Res. 2026 Apr 16;10:e91438. doi: 10.2196/91438 (PMC13085982; doi:10.2196/91438)
Supplement: Multimedia Appendix 1 [file formative-v10-e91438-s001.docx]

Patient Informed Consent Form

The Kidney Beam Trial – The Ex-Tab Sub-study

**A study to evaluate the acceptability of providing digital support to improve engagement with an education and exercise online physical and emotional wellbeing resource for the improvement of health-related quality of life in people with chronic kidney disease. The Ex-Tab Sub-study**

**IRAS Number: IRAS 291403**

**Chief Investigator: Dr Sharlene Greenwood**

**Patient** **Name: KCH Hosp No:**

| **1.** | I confirm that I have read the information sheet dated 07.12.2022 (version 1.0) for the above study. I have had the opportunity to consider the information, ask questions and have had these answered satisfactorily. | **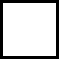** |
| --- | --- | --- |
| **2.** | I understand that my participation is voluntary and that I am free to withdraw at any time without giving any reason, without my medical care or legal rights being affected. | **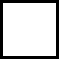** |
| **3.** | I understand that the patient interviews will be recorded and that these are optional. I may, or may not, be invited to take part in these. | **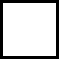** |
| **4.** | I understand that relevant sections of my medical notes, contact details and data collected during the study, will be shared with individuals from the central research site, which is also the sponsor of the trial (King’s College Hospital NHS Trust). This will also include responsible persons authorized by the sponsor, from regulatory authorities or from an NHS Trust, where it is relevant to my taking part in this research. I give permission for these individuals to have access to my records. | **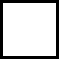** |
| **5.** | I consent to the information collected about me, including audio recordings (where participants have been invited to interview), to be used to support other research in the future, and that this may be shared anonymously with other researchers. | **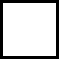** |
| **6.** | I agree to my General Practitioner being informed of my participation in the study. | **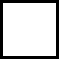** |
| **7.** | I agree to be contacted about ethically approved future research. | **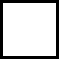** |
| **8.** | I wish to receive the final report at the end of the study. | **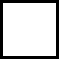** |
| **9.** | I understand that if I receive an iPAD device it is for a loan period of **16 weeks** and will be returned to Kings College Hospital NHS Trust on an agreed date. | **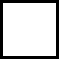** |
| **10.** | I agree not to enter any personal or sensitive data (including financial information) into the iPad device, for my own safety and security. If I have any concerns or questions about this I will contact the Renal Research Team. | **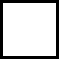** |
| **11.** | I understand that the iPAD device provided for the Ex-Tab Sub- study  will be remotely managed by the Renal Research Team at King’s College Hospital for the duration of the study and that the team will be able to track the location of the device on a map and view information about its use, in order to help participants, and assist in the event of loss or theft of the device. | **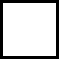** |
| **12.** | I agree to take part in the Kidney Beam Trial – The Ex-Tab Sub study | **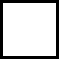** |

Name of Participant……………………………….Signature……………………………………Date………………..

Name of Person

taking consent……………………………………..Signature…………………………………….Date……………….

When completed: 1 for participant; 1 for researcher site file; 1 (original) to be kept in medical notes and scanned onto the computer
