# Supplementary material for: A Digital Inclusion Intervention to Improve Access to a Digital Health Intervention Among Digitally Excluded Adults: Mixed Methods Pilot Randomized Controlled Trial
Source: JMIR Form Res. 2026 Apr 16;10:e91438. doi: 10.2196/91438 (PMC13085982; doi:10.2196/91438)
Supplement: Multimedia Appendix 2 [file formative-v10-e91438-s002.docx]

# PROTOCOL FULL TITLE: Multicentre prospective single-blind wait-list randomised controlled trial, with nested pilot, of the clinical value and economic evaluation of an online physical and emotional wellbeing resource for the improvement of health-related quality of life in people with chronic kidney disease: The Kidney Beam Trial

| **Protocol Short Title/Acronym:** The Kidney Beam Trial |
| --- |

### Version and date of protocol Version 9.0 12.01.2023

### ClinicalTrials.gov no. NCT04872933

### IRAS number 291403

### REC no. 21/LO/0243

### Sponsor:

| King’s College Hospital NHS Foundation Trust  Contact: Rahman Ahmed  Address: King’s College Hospital, 161 Denmark Hill, London, SE5 8EF  Telephone: 02032996625    Email: rahman.ahmed1@nhs.net  **Funder:**  Kidney Research UK  Address: Nene Hall, Petersborough Business Park, Lynch Wood, Peterborough, PE2 6FZ  Telephone: 0300 303 1100 |
| --- |
| **Chief Investigator**  Dr Sharlene Greenwood  Renal Unit, King’s College Hospital, London, SE5 9RS  Telephone: 0203 299 6233  Email: sharlene.greenwood@nhs.net |

**Name and address of Co-Investigators**

Jamie Macdonald

School of Sport, Health and Exercise Sciences, Bangor University. George Building, Bangor, Gwynedd, LL57 2PZ

Telephone: 01248 383272

Email: j.h.macdonald@bangor.ac.uk

Ms Juliet Mayes

Renal Unit

King’s College Hospital

London, SE5 9RS

Telephone: 0203 299 6233

Email: Juliet.mayes@nhs.net

Dr Hannah Young

Leicester Kidney Lifestyle Team

Academic Unit

Leicester General Hospital

Leicester

Telephone: 0116 2584346

Email: Hannah.young@uhl-tr.nhs.uk

Dr Zoe Saynor

School of Sport, Health and Exercise Science

University of Portsmouth

Honorary Research Portsmouth Hospitals University NHS Trust

Telephone: 02392 843080

Email: [zoe.saynor@port.ac.uk](mailto:zoe.saynor@port.ac.uk)

Dr Robert Lewis

Portsmouth Hospitals University NHS Trust

Wessex Kidney Centre,

G Level, Queen Alexandra Hospital

Portsmouth PO6 3LY

Email: robert.lewis@porthosp.nhs.uk

Dr Andrew Nixon

Lancashire Teaching Hospitals NHS Foundation Trust

Royal Preston Hospital, Sharoe Green Lane, Fulwood , Preston, Lancashire, PR2 9HT Telephone: 01772 716565

Email: andrew.nixon3@nhs.net

Professor Nicolette Bishop

School of Sport, Exercise and Health Sciences,

Loughborough University, Loughborough, LE11 3TU

Telephone: 01509 226385

Email: N.C.Bishop@lboro.ac.uk

Dr Ellen Castle

Renal Unit

King’s College Hospital

London, SE5 9RS

Telephone: 0203 299 6233

Email: ellen.castle@nhs.net

Dr Kate Bramham

Renal Unit

King’s College Hospital

London, SE5 9RS

Telephone: 0203 299 6233

Email: kate.bramham@nhs.net

Dr Thomas Wilkinson

Leicester Kidney Lifestyle Team

Department of Health Sciences

University of Leicester

Leicester

LE17RH

Telephone: 0116 2584346

Email: tjw26@le.ac.uk

Ms Roseanne Billany

Department of Cardiovascular Sciences

University of Leicester

Telephone: 0116 2584042

Email: r.billany@leicester.ac.uk

Dr Matthew Graham-Brown

Department of Cardiovascular Sciences

University of Leicester

Telephone: 0116 2584042

Email: mgb23@le.ac.uk

Dr Joseph Chilcot

King’s College London, SE1 9RT

Telephone: 02071887188

Email: joseph.chilcot@kcl.ac.uk

Dr Helen Noble

School of Nursing and Midwifery,

Queen’s University Belfast

Telephone: 0044 7793494348

Email: [helen.noble@qub.ac.uk](mailto:helen.noble@qub.ac.uk)

Dr Alexander Hamilton

Royal Devon and Exeter NHS Foundation Trust

Royal Devon and Exeter Hospital, Barrack Road , Exeter, Devon, EX2 5DW Telephone: 01392 411611 Email: [alexander.hamilton@bristol.ac.uk](mailto:alexander.hamilton@bristol.ac.uk)

**Statistician**

Professor Jackie Campbell

University of Northampton

Northampton NN1 5PH

Telephone: 01604 892010

Email: [jackie.campbell@northampton.ac.uk](mailto:jackie.campbell@northampton.ac.uk)

**Health Economist**

Professor Nicola Cooper

University of Leicester

Email: njc21@leicester.ac.uk

**Patient partners**

Ms Madeleine Warren

Ms Sarah GreenProtocol Synopsis

| Title of clinical trial |  | Multicentre prospective single-blind wait-list randomised controlled trial of the clinical value and cost-effectiveness of an online physical and emotional wellbeing resource for the improvement of health-related quality of life in people with chronic kidney disease: The Kidney Beam Trial |
| --- | --- | --- |
| Protocol Short Title/Acronym |  | **The Kidney Beam Trial** |
| Trial Phase if not mentioned in title |  | Phase IV |
| Sponsor name |  | King’s College Hospital NHS Trust |
| Chief Investigators |  | Dr Sharlene Greenwood |
| ClinincalTrials.gov number |  | NCT04872933 |
| REC number |  | 21/LO/0243 |
| Medical condition or disease under investigation |  | Chronic Kidney Disease (CKD) |
| Purpose of clinical trial |  | This is a multi-centre single-blind waitlist randomised controlled trial (RCT) that will examine the clinical value and cost-effectiveness of an online physical and emotional wellbeing resource for the improvement of health-related quality of life in people with CKD. |
| Trial objectives |  | 1. To assess the clinical value and cost-effectiveness of an online physical and emotional wellbeing resource for the improvement of health-related quality of life in people with CKD. 2. To assess healthcare utilisation and associated cost of an online physical and emotional wellbeing resource for the improvement of health-related quality of life in people with CKD. 3. To assess mental health, physical function, physical activity, patient activation, fatigue, and clinical measures. 4. To explore the acceptability of the trial procedures and the BEAM platform (short and longer-term). |
| Trial Design |  | An investigator-led multicentre waitlist RCT of participants with CKD |
| Primary outcome measure |  | Mental Composite Score (MCS) of KDQOL-36 quality of life questionnaire at 12 weeks. |
| Main secondary outcome measures |  | - *EQ5D-5L questionnaire* - *Patient Activation Measure (PAM-13)* - *The Patient-Health Questionnaire-4* - *Chalder Fatigue Questionnaire -- physical and mental fatigue* - *Work and Social Adjustment Scale (WSAS)* - *Functional capacity (sit-to-stand 60 to assess lower limb function)* - *Global Physical Activity Questionnaire (GPAQ)* - *Quality of life (KDQOL-36 physical composite score, energy/fatigue, burden of kidney disease, role physical, physical functioning, mental health, bodily pain, role emotional, social functioning, general health)* - *Patient engagement with the digital platform* - *Qualitative exploration of acceptability and participant experience* - *Healthcare utilisation* - *Adverse events* |
| Sample Size |  | 304 participants |
| Summary of eligibility criteria |  | Patients with established CKD  Aged 18 years+  Patients who are naïve to Kidney Beam intervention and who have not participated in a structured exercise programme in the prior 3 months |
| Intervention |  | Kidney Beam digital health intervention platform – physical activity and emotional wellbeing via live and on demand video content |
| Active comparator product(s) |  | Waitlist control group |
| Maximum duration of treatment of a  subject |  | 3-month intervention, 6-month follow-up (9 months in total) |
| Project costs |  | £159,872 |

### Glossary of Terms

| **AE** | Adverse event |
| --- | --- |
| **AT** | Aerobic training |
| **CKD** | Chronic Kidney Disease |
| **CTO** | Clinical Trials Office |
| **CRP** | C-Reactive Protein |
| **eGFR** | Estimated glomerular filtration rate |
| **GCP** | Good Clinical Practice |
| **Hb** | Haemoglobin |
| **ICF** | Informed consent form |
| **ICH** | International Conference on Harmonisation |
| **HRA** | Health Regulatory Agency |
| **QoL** | Quality of life |
| **RCT** | Randomised Controlled Trial |
| **REC** | Research Ethics Committee |
| **RT** | Resistance training |
| **SAE** | Serious adverse event |
| **STS60** | Sit-to-stand 60 |
| **UK** | United Kingdom |

### CONTENTS

1. Protocol Synopsis…………………………………………………………………… ….4

2. Glossary of Terms ……………………………………………………………… ….....6

3. Background & Rationale 7

4. Trial Objectives and Design 9

4.1. Trial Objectives 9

4.1.1 Primary Outcome Measure…………………………………………………….…….....10

4.1.2 Secondary Outcome measure …………………………….………………….…… .... 10

4.2 Trial Design 10

4.3 Trial Flowchart 13

4.4 Study Procedures table………………………………………………………….………..14

**5 Blinding and Unblinding …………………………………………………………………..15**

6 Selection and Withdrawal of Subjects …………..…..17

6.1 Inclusion Criteria 15

6.2 Exclusion Criteria 16

6.3 Selection of Participants 16

6.4 Randomisation Procedure 16

6.5 Withdrawal of Subjects 17

7 Study Procedures 17

7.1 Informed Consent 17

7.2 Screening 18

7.3 Baseline / Randomisation Visit…………………………………………..……………… 18

7.5 Exercise Training………………………………………………………………………… .20

7.6 Week 12 Visit and 6-month visit…………………………………………………………………………………………….…21

7.7 Qualitative Exploration of Patient Experience…………………………………………..21

7.8 Qualitative Sub study – Digital Inclusion ………………………………………………..23

7.9 Expected Duration of Trial………………………………………………………………...23

8 Laboratory Tests 24

9 Assessment of Safety 24

9.1 Specification, Timing and Recording of Safety Parameters. 24

9.2 Procedures for Recording and Reporting Adverse Events 24

10 Statistics 24

10.1 Sample Size 24

10.2 Randomisation 24

10.3 Analysis 24

11. Trial Steering Committee 25

12. Data Monitoring Committee 25

13 Ethics & Regulatory Approvals 25

14 Data Management 26

**15 Database…………………………………………………………………………………….26**

16 Insurance & Indemnity 26

17 Financial Aspects 26

**18 Publication Policy ……………………………………………………….…………..……26**

19 Project Costs……………………………………………………………………………….28

20 Signatures …....29

**21 References …………………………………………………………………………………30**

### 3. Background & Rationale

Physical inactivity and poor mental health are very real concerns for people living with kidney disease, and they report multiple symptoms that impact upon the physical component of health-related quality of life (HRQoL) (1). A decrease in the physical component of HRQoL is independently associated with mortality and morbidity (2,3). In people living with end stage kidney disease (ESKD), systematic reviews indicate that a range of exercise training interventions improve physical function and alleviate disability symptoms (4-18). The physical component of HRQoL can be targeted with interventions to enhance physical activity, however people living with kidney disease are still not routinely offered specialist physical activity or mental health support in the NHS. Fatigue is a complex array of symptoms described as ‘extreme and persistent tiredness, weakness or exhaustion-mental, physical, or both’ (19-21). Fatigue is present across the full spectrum of chronic kidney disease (CKD), affecting patients not yet requiring renal support. It is a common complaint in ESKD, affecting from 42 to 89 percent of patients, and persists in those who subsequently received a renal transplant (21,22). In ESKD, fatigue has huge repercussions on HRQoL, further impairing patients’ daily functioning, motivation, social engagement (4,23-26), and contributing to poorer sleep quality and increased bodily pain (23,24,27). There is also evidence to suggest that fatigue may contribute directly to clinical outcomes, increasing the risk of cardiac events and mortality (25). A UK-wide MDT survey revealed significant barriers to the provision of exercise counselling including; a lack of available exercise professionals and facilities and difficulty with the wide geographical reach of the CKD patient population who attend tertiary care clinics, as well as a lack of funding (29). A recently published joint statement from The Centre for Mental Health and Kidney Research UK highlighted the very real need for improved access to services that promote physical and mental wellbeing for people living with kidney disease (30). This has only become more urgent as a result of the Coronavirus pandemic, which has further reduced the physical activity levels of those living with CKD and undoubtedly negatively influenced the quality of life of people living with CKD, many of whom have been required to shield during this time.

Kidney Beam [https://beamfeelgood.com/home](about:blank) is a new wellbeing digital health intervention platform that was developed, and launched, to help people with kidney disease manage their physical and mental health through the Coronavirus 2019 (COVID-19) pandemic, resulting lockdown and beyond. Early in the COVID-19 pandemic, people with CKD were identified as being at an increased risk of more severe infection from this virus (31) and a mortality rate of 15.2% mortality rate, much greater than the general population was reported in the UK (32). Consequently, these individuals were advised to take extra precautions to minimise their risk of exposure (31) which, in the UK, resulted in re-classification of these individuals as ‘extremely clinically vulnerable’ to COVID-19 on Thursday 23^rd^ April 2020 and shielding at home. Kidney Beam was developed to offer people to help kidney patients manage their physical and mental health through the Coronavirus pandemic lockdown and beyond. Kidney Beam offers people living with kidney disease a way to improve their physical activity and boost their mental health through live and on demand movement classes and expert educational videos, while remaining in their own home. The free online service was a collaboration between King's College Hospital, Kidney Research UK and Beam, a digital health intervention platform supporting people with health conditions to stay physically active. Kidney Beam is led by specialist kidney professionals, including renal physiotherapists and renal counsellors, from a number of different NHS Trusts and backgrounds, as well as people living with kidney disease. The resource is aimed at anyone over 18 with any ability, any kidney condition, and at any stage of kidney disease. The 6-month pilot will end on the 30^th^ November 2020. The team are seeking funding to extend the free access to the resource for a further 12-months while the team evaluate the clinical value and cost effectiveness of the digital health intervention platform. The Kidney Beam project has been highlighted as a key project that will be supported by the Renal Transformation Plan, having already been discussed and reviewed by the Renal Clinical Reference Group (CRG) and NHS England for potential commissioning following the 12-month study.

Our patient advisory group, who have been integral to shaping the Kidney Beam digital health intervention platform, strongly recommended that any resource include a variety of exercise classes for patients to do at home, and emotional support from healthcare professionals. Kidney Beam has already attracted in excess of 700 sign-ups from across the UK and has been well-received by people living with kidney disease, and the renal healthcare professionals alike. As an online intervention, Kidney Beam, has the potential to overcome the well documented geographical access barriers to specialist physical activity and emotional support services for patients with CKD (33,34) and represents an efficient use of limited resources in a financially unstable NHS. Indeed, the NHS 5-year forward view and the Carter report highlight the need to look at digital solutions as a way to promote efficiency in the NHS (35,36).

### 4. Trial Objectives and Design

### 4.1. Trial Objectives

To conduct a multi-centre waitlist RCT that will examine the clinical value and cost-effectiveness of an online physical and emotional wellbeing resource for the improvement of health-related quality of life in people with CKD. The study will assess the clinical value of the Kidney Beam digital health intervention platform, as assessed by the Mental Component Score (MCS) of the KDQOL-36 questionnaire, in people living with CKD. The study will also examine whether the Kidney Beam digital health intervention platform is cost effective, and can improve mental and physical health, physical function, fatigue, and patient activation.

**PRIMARY OBJECTIVE**

1. To assess the clinical value of the Kidney Beam digital health intervention platform, as assessed by the MCS of the KDQOL-36 HRQoL questionnaire.

**SECONDARY OBJECTIVES**

1. To assess cost effectiveness, mental and physical health, physical function, quality-of-life, patient activation, and fatigue in people living with CKD.
2. To explore the perceptions of people living with CKD in relation to Kidney BEAM, and their experience of using the platform

Specifically, we propose to examine whether a physical and emotional wellbeing package – Kidney Beam - can improve the mental composite score of the KDQOL-36 QoL questionnaire.

The results of this trial will provide data to support the clinical commissioning of the Kidney Beam digital health intervention platform.

**4.1.1 Primary outcome measure**

Mental HRQoL as measured by MCS of the KDQOL-36 at 12 weeks.

**4.1.2 Main secondary outcome measures**

- *Patient activation measure (PAM-13)*
- *Chalder Fatigue Questionnaire -- physical and mental fatigue*
- *Patient Health Questionnaire-4 (PHQ-4)*
- *Functional capacity (sit-to-stand 60 to assess lower limb function)*
- *Quality of life (KDQOL-36 physical component score (PCS), energy/fatigue, burden of kidney disease, role physical, physical functioning, mental health, bodily pain, role emotional, social functioning, general health)*
- *Global Physical Activity Questionnaire (GPAQ)*
- *Clinical Frailty Score*
- *Work and Social Adjustment Scale*
- *Qualitative exploration of intervention and trial acceptability and participant experience*
- *Adverse events*
- *Kidney Beam platform metrics (physical activity minutes, engagement)*

**4.2 Trial Design**

An investigator-led multicentre single-blind waitlist randomised controlled trial (with nested pilot study) of participants with established chronic kidney disease (CKD).

**Nested pilot study**

The study will adopt a nested pilot design to preliminarily assess trial and intervention feasibility. To assess this, a range of *a priori* progression criteria based on a traffic light system will be utilised to determine if the study should be halted (red), changes to the design or intervention considered (amber) or is considered feasible and progress to the full trial (green). These progression criteria will be co-developed with a panel of experts and stakeholders including patients, healthcare professionals and researchers.

**Feasibility outcomes**

At 12 weeks, outcomes pertaining to the feasibility of the intervention and trial will be assessed following each phase of the trial. This will be for the overall trial sample at 12 weeks, and for the first 3 external sites that are opened in the study.

o Recruitment rate

The feasibility of recruitment and interest of patients is an essential component of whether the full trial is feasible. The number of eligible patients and number of consented will be recorded. Monthly recruitment rate and the time taken to recruit 25%, 50%, 75%, and 100% patients will be recorded.

o Acceptability of randomisation and assessment procedure

Acceptability of randomisation and procedures, and by measuring loss to follow-up and by exploring patient’ views about their participation in the research

o Adherence to intervention

Adherence will be assessed by the completion of the exercise sessions. We will also assess patterns and frequency of programme usage.

o Attrition rate

The number of drop-outs (attrition rate) in the study will be recorded.

o Missing data

Quantity of missing data (e.g., questionnaire completion rate, outcome measures not completed)

o Trial and intervention acceptability

This will be attained through qualitative interviews in a sub-set of patients.

**Progression criteria**

The decision regarding trial feasibility will be made using a composite assessment of both quantitative and qualitative data based on a traffic light system (‘green’ (go), ‘amber’ (amend), and ‘red’ (stop) using pre-defined criteria (37,38).

The main criteria contributing to this system are:

o recruitment rate (patients consented/approached)

o adherence to intervention

o acceptability of randomisation and assessment procedures

o attrition rate

o missing data

o The opening of secondary research sites to commence the trial

Relative 'green’, ‘amber’, and ‘stop’ criteria will be formed prior to the intervention through our group’s previous experience, discussion with collaborators and patient advisory members, and other literature (39). Analysis against the stop-go criteria will be undertaken at the end of the pilot phase (40).

Assessments will be performed at baseline, and after 12 weeks, and will compare the Kidney Beam digital health intervention platform with a waitlist control group.

**BEAM trial progression criteria at 12 weeks, for internal feasibility study**

**Eligibility**

No set progression criteria, but each centre will be required to report the numbers in each population of interest (pre-dialysis, PD,HD, transplant) who have been screened, and the proportion recruited to ensure that a representative population is being targeted.

**Recruitment:**

On the basis of 8 centres, aiming to recruit n=304 within 9 months.

Go:

- 15 participants recruited within 12 weeks per centre.

Amber

- Less than 15 recruited per centre, but other centres are able to make up the shortfall

Stop

- 3 centres are unable to open to recruitment

**Acceptability of randomisation:**

The characteristics (and the proportion by group) of participants withdrawing post randomisation will be captured to determine the acceptability of the waitlist design.

**Intervention acceptability:**

The proportion of participants attending live or on-demand classes via the Kidney Beam platform at 12 weeks will be reviewed.

Go:

- Engagement with at least one BEAM class per week for 70% participants

Amber:

- Engagement with at least one BEAM class per week for 60% participants

Stop:

- Engagement with at least one BEAM class per week in<50% participants

**Outcome acceptability:**

For the primary outcome (KDQoL):

Go:

- At least 80% completion rate

Amber:

- At least 70% completion rate

Stop:

- Less than 60% completion rate

**Retention**

Initial dropout rate will be considered as not all participants will have completed the programme by the end of the 12-week pilot phase

Go:

- Less than 20% drop out rate

Amber:

- Less than 40% drop out rate

Stop:

- More than 50% drop out rate

### 4.3 Trial Flowchart

**INCLUSION CRITERIA**

1. Patients with established CKD
2. Men and women aged 18yrs+
3. Access to a wifi-enabled device
4. Able to understand written English language
5. Written or virtual informed consent

**SELECTION OF PARTICIPANTS**

CKD patients

Age 18yrs+

- **4.4 Study procedures (all remote): Screening to 12 weeks**

**EXCLUSION CRITERIA**

1. Weight < 50kg
2. Active infection
3. Participation in structured exercise programme or Kidney Beam resource in prior 3 months
4. Uncontrolled arrhythmias
5. Unstable angina or heart attack within the last 3 months
6. Persistent uncontrolled hypertension (systolic blood pressure >180 mm Hg or diastolic blood pressure >110 mm Hg)
7. Recent (within the last 3 months) stroke or transient ischaemic attack
8. Receiving palliative care for advanced terminal cancer
9. Patients with peripheral vascular or musculoskeletal disease, who the investigator deems unable to carry out a physical activity intervention.
10. Any other health condition considered by the local Principal Investigator in which physical activity will be contraindicated.
11. Insufficient understanding of the trial

**RANDOMISATION/BASELINE Assessment Visit**

Not for

COVID

Sub-study

Post COVID functional assessment tool

NO

YES

History of COVID

**12 WEEK Assessment Visit**

**12 weeks:**

**Kidney Beam intervention**

**Non-waitlist**

Not for

COVID

Sub-study

Post COVID functional assessment tool

NO

YES

**12 weeks Kidney Beam intervention**

**12 WEEK Assessment Visit**

**12 weeks:**

**Normal routine care**

History of COVID

**Waitlist**

**SCREENING**

Inclusion and Exclusion criteria

**Patient Information Sheet** **/ Consent Form**

Patient Information Sheet given to potential patients.

Consent form signed.

| PROCEDURE | | Screen Visit | End of 12 week pilot phase | Baseline/Randomisation Visit | Week 12 Visit | 6-month follow-up |
| --- | --- | --- | --- | --- | --- | --- |
| Patient information and informed consent | | X |  |  |  |  |
| Inclusion / Exclusion criteria | | X |  |  |  |  |
| Demographic data and medical history | | X |  |  |  |  |
| Height, weight, BMI, Clinical Frailty Scale | |  |  | X | X |  |
| **Vital signs (Blood pressure & heart rate) | |  |  | X | X |  |
| **Full blood count | |  |  | X | X |  |
| **Creatinine, CRP, eGFR, Hb, IL-6, Albumin from routine bloods | |  |  | X | X |  |
| Fatigue Severity Score (Chalder Fatigue questionnaire) | |  |  | X | X |  |
| Functional impairment (WSAS Questionnaire) | |  |  | X | X |  |
| Quality of life (KDQOL-36 and EQ5D-5L) | |  |  | X | X | X |
| Functional capacity (STS60) |  | |  | X | X |  |
| Global Physical Activity Questionnaire (GPAQ), and Physical Health Questionnaire (PHQ9) and additional questions on meeting current physical guidelines. |  | |  | X | X |  |
| Patient Activation Measure |  | |  | X | X |  |
| Qualitative Interview |  | | X |  | X |  |
| Adverse Events |  | |  | X | X |  |
| Kidney Beam platform metrics (PA mins and engagement) |  | |  |  | X | X |
| Healthcare utilisation questionnaire |  | |  | X | X | X |
| ***Post-COVID functional assessment tool questionnaire |  | |  | X | X | X |
| ****Digital health literacy screening tool | X | |  |  |  |  |
| ^*****^Hand grip Strength |  | |  | X | X |  |

** This procedure will only be a retrospective review of most recent available routine blood tests results and vital sign measurements. Results with date will be recorded by the study team. No new blood test will be collected from patient.

*** Only patients who had history of COVID at time of consent will be assessed for Post-COVID functional assessment tool questionnaire at Baseline.

****Only a sub set of patients from Kings College Hospital.

^*****^Only in a subset of patients at Newcastle NHS Trust

**COVID-19 Sub-study**

The outcomes for patients with advanced CKD, patients with kidney transplants and patients on dialysis are amongst the worst of all patient groups (41). Not only are mortality rates significantly higher than in the general population, but patients with CKD are more likely to develop the most severe complications of COVID and are more susceptible to physical deconditioning from acute illness (42). The long-term effects of COVID-19 on physical activity, cardio-respiratory fitness and sarcopenia are also not known for patients with CKD. Additionally, acute kidney injury complicates up to 25% of patients admitted to intensive care units with COVID (43). It is anticipated that a proportion of patients that survive will have varying degrees of CKD and programs of post-COVID rehabilitation do not exist for either patients previously known to have CKD or patients recovering from the effects of COVID-induced AKI.

The Kidney Beam trial will contain an exploratory sub-study for any patients with CKD who had COVID-19 or who have been left with CKD following AKI-related to COVID-19 infection. At the baseline study (below) all patients will be asked about whether they have had a positive test for COVID-19. For those who had a positive test, an assessment of the severity of their COVID will be made in the following way:

- COVID positive, managed at home not requiring hospital admission
- COVID positive, required hospital admission but no oxygen therapy
- COVID positive, required hospital admission and oxygen therapy
- COVID positive, required hospital admission and non-invasive ventilation
- COVID positive, required hospital admission and admission to intensive care

Additional details of the admission, including length of stay and time since discharge will be recorded. Subjects will continue in the study in the same way but will complete the post-COVID functional status tool questionnaire at baseline and follow-up in addition to other outcome measures (44).

This exploratory sub-study will be a low-cost, high-yield way of gathering data to inform the design of more bespoke studies and interventions to meet the needs of post-COVID CKD patients and those recovering from the effects of post-COVID AKI.

**Polycystic Kidney Disease Sub-study**

Recommendations by ‘Kidney Disease: Improving Global Outcomes’ (KDIGO) for individuals affected by and at-risk of PKD include a healthy lifestyle and diet, maintenance of optimal weight, regular exercise, and avoidance of smoking. Nevertheless, despite these recommendations, little is known about the effectiveness of PKD-specific lifestyle modifications in improving quality of life, physical function and patient activation. The Kidney Beam platform, which is now widely available for people living with kidney disease in the UK, is an ideal place to create a bespoke education and exercise training module to support people living with PKD to engage with physical activity.

The Kidney Beam Trial will contain an exploratory sub-study for 12 patients with PKD. Those patients will be directed to complete a PKD-specific education module prior to starting the exercise training as per protocol. Participants will complete baseline and 12-week assessments as per protocol, and will be invited to feedback on the acceptability of the module via semi-structured interviews.

**Haemodialysis Sub-study**

A sub-study will be conducted to explore the impact of using kidney beam within an in-centre haemodialysis patient population. This sub-group of patients have a high physical health burden and this will help development of the platform to incorporate the needs of different users, with multi-morbidity and varied health needs who may normally find it more difficult to undertake physical exercise.

Evidence in intradialytic exercise programs is difficult to draw robust conclusions from as studies conducted in this area have yielded mixed results historically. An easily accessible exercise platform with the option for patients to utilise this whilst receiving their dialysis treatment could be beneficial.

A small group of haemodialysis patients (60) will be enrolled to this sub-study based on the inclusion and exclusion criteria listed in Section 4.3 with the additional inclusion criteria of:

- Receiving haemodialysis in-centre
- No instability on dialysis in the preceding 4 weeks prior to baseline visit
  - i.e. no episodes of intradialytic hypotension, early cessation of sessions for medical reasons or chest pain precipitated by dialysis

In line with the aforementioned goals of the Kidney Beam study, the following (taken from Section 7.3) will be measured pre and post 12 week kidney beam program with a view to ascertaining the benefit this population group receives:

- Physical functional parameters
  - Sit-to-Stand 60
  - Hand grip strength (in anticipation that some haemodialysis patients will have lower limb amputation or have lower limb disability)
- Quality of life measure (EQ5D-5L)

The patients will be separated equally in to three separate arms to evaluate the relevant benefits of supportive interventions in conjunction with kidney beam:

**Arm 1:** Access to Kidney Beam program

**Arm 2:** Access to Kidney Beam program plus equipment available for use on dialysis (exercise equipment plus IT equipment for access to kidney beam) – this will tie in with the other sub-study outlined in Section 7.8.

**Arm 3**: Access to kidney beam, available equipment on dialysis, regular visits from on-site staff – a mixture of both medical and physiotherapy staff – to provide practical tips on use of the platform, encouragement and motivation to continue with the program and supportive exercise options from the physiotherapy team.

This is with a view to building the knowledge base around exercise in dialysis patients to ascertain the level of intervention required to see beneficial changes.

Patient information sheets with be provided and informed consent will be taken face-to-face. Baseline data will be collected at initial visit as per methods in Section 7.3 but only measuring a selected number of parameters, as outlined above and then again at the 12 week visit as per Section 7.5, again measuring those select data parameters.

**Ex-Tab Sub-study**

The recruitment process for the Kidney Beam Trial excluded numerous participants due to not having Wi-Fi access, a suitable device to use and reports of participants not feeling confident to access an exercise platform online. This showed a significant number people living with CKD missing out on the opportunity to participate in the study. There is currently limited digital inclusion research of people living with CKD.

The Kidney Beam Trial will contain an exploratory sub-study of 40 participants from Kings College Hospital who do not have access to a digital device or who are not confident using the technology available to them. The participants will be randomised to receive the Kidney Beam platform intervention as per protocol or the exercise tablet intervention.

Participants will complete baseline and 12 week assessments as per protocol with the only difference being that a screening tool will be included to assess digital health literacy prior to inclusion and consent. All assessments for the participants in the exercise tablet intervention group will receive a face to face assessment, provision of a technology tablet, Wi-Fi access if needed, and training on how to use the device. The exercise tablet intervention group will be loaned a technology tablet for 12 (+- 2 weeks) so that they can access the Kidney Beam platform and engage in live or on demand classes.

Inclusion criteria:

Patients with established CKD

Aged 18 years+

Patients who are naïve to Kidney Beam intervention and who have not participated in a structured exercise programme in the prior 3 months

Patients that do not have an electronic device.

Patients that have self-reported low confidence using electronic devices.

Patients that do not have Wi-Fi access or data.

Exclusion criteria as per main study protocol.

### 5. Blinding

**5.1 Blinding**

Single-blind trial design whereby the Research Assistant who will conduct the outcome measures at baseline and 12 weeks will not be aware of the participant allocation.

### 6. Selection and Withdrawal of Subjects

### 6.1 Inclusion Criteria

- Individuals with established CKD
- Adults aged 18yrs+
- Access to a Wi-Fi-enabled device
- Able to understand written English language
- Written or virtual informed consent

### 6.2 Exclusion Criteria

- Weight < 50kg
- Self-reported participation in a structured exercise programme or kidney beam digital health intervention platform within previous 3 months
- Active infection
- Uncontrolled arrhythmias
- Unstable angina or heart attack within the 3 months
- Persistent uncontrolled hypertension (systolic blood pressure >180 mm Hg or diastolic blood pressure >110 mm Hg)
- Recent (within the last 3 months) stroke or transient ischaemic attack
- Receiving palliative care for advanced terminal cancer
- Patients with peripheral vascular or musculoskeletal disease, who the investigator deems unable to carry out a physical activity intervention.
- Any other health condition considered by the local Principal Investigator in which exercise therapy will be contraindicated.
- Insufficient understanding of the trial

**6.3 Selection of Participants**

Three hundred and four patients aged 18 years or over with established CKD will be enrolled. Each patient must meet all of the inclusion criteria, and none of the exclusion criteria, at entry to the trial. Patients who meet the entry criteria may be recruited by the investigator or any member of the local trial team who has delegated responsibility for trial recruitment.

The trial will be eligible for Clinical Research Network adoption. Replicating the study set-up and recruitment methodology employed in the Surveying People Experiencing young Adult Kidney failure (45), following Health Research Authority approval the trial will aim to run in all UK kidney units, allowing equitable access to the trial for all people with kidney disease in the UK. Local research nurses will identify and recruit patients and will be able to act as site Principal Investigators. Upon recruitment, the central trial research assistant

based at Kings College Hospital, will perform baseline assessments. Potential trial participants will be identified when presenting for their routine hospital clinic visits. Additionally, the local trial team may write to the patient, or phone them, to inform them of the study.

### 6.4 Randomisation Procedure

Randomisation (1:1) will be done on a secure web-based service. After passing the screening visit and when all eligibility criteria have been confirmed and informed consent has been received, the participants can be randomised into the study. Participants will be randomised using an approach based on randomly varying block sizes.

Randomisation will be done only by an unblinded member of staff who has been delegated this responsibility by the principal investigator as evidenced by documentation in the delegation log. The web-based database will allocate the participant a unique trial identification number and their identification details will be entered onto the trial identification log, which is kept in the Investigator Site File.

### 6.5 Withdrawal of Subjects

## Participants have the right to withdraw from the study at any time for any reason. The investigator also has the right to withdraw patients from the study treatment in the event of intercurrent illness, AEs, SAE’s, protocol violations, administrative reasons or other reasons. It is understood by all concerned that an excessive rate of withdrawals can render the study uninterpretable; therefore, unnecessary withdrawal of patients should be avoided. Withdrawn patients will not be replaced.

##

Withdrawal from study

## Should a patient decide to withdraw from the study, all efforts will be made to report the reason for withdrawal in as much detail as possible. Patients will continue to attend assessment visits and be followed up for adverse events.

Consent withdrawal

Participants have the right to withdraw their consent from further participation in the study at any time. Should a patient decide to withdraw consent, all efforts will be made to report the reason for withdrawal in as much detail as possible. After consent withdrawal, no further study data should be recorded or entered onto the database, although data queries arising from data recorded prior to the withdrawal of consent may be answered. The follow-up of participants who formally withdraw their consent will be censored at the date of withdrawal of consent.

### 7. Study Procedures

**7.1 Informed Consent**

Members of the local study team will screen for potential eligible study participants using the inclusion/exclusion criteria. The Principal Investigator leading the study team will confirm patient’s eligibility. Referrals from clinicians or clinical care team will also be screened for eligibility. After confirming potential eligibility, patients will be approached by an appropriately trained member of the study team to ascertain interest in entering the study. This individual will give a comprehensive verbal explanation of the trial (explaining both the intervention and the waitlist nature of the study and highlighting any possible benefits or risks relating to participation). Potential participants will be given adequate time throughout the discussion to ask any questions, and these will be suitably addressed. They will also be given a written information sheet about the trial and be given sufficient time (minimum 24 hours) to read and consider the study information prior to deciding whether to take part. If the participant is willing to take part, then they will be asked to sign the consent form. Remote recruitment and consent via telephone or video-conference will be utilised for those participants who are unable to attend a hospital visit.

No clinical trial procedures will be conducted prior to taking consent.

**7.2 Screening**

All data, as detailed below and in the Trial Flowchart, must be collected and all required procedures performed, to assess the subject eligibility for the study.

- Demographic assessments and baseline characteristics
  - Routine clinical information will be extracted from the medical notes and patient records including but not limited to: age, gender, ethnicity, cause of kidney disease, transplant type (if applicable), time since transplant (if applicable), dialysis-duration (if applicable), comorbidities, blood/urine results, medications, smoking habits, diabetes status, CV events and risk factors
- CRP, Creatinine, eGFR

A physical examination is not required in this study.

Patients who fail initial screening may be re-screened with there being no limit to the number of times a patient can be re-screened. Patients must agree to be rescreened and the discussion should be documented in the patient’s notes. Patients must be re-consented if the re-screening visit is more than 3 months from the initial patient consent.

Patients who pass a screening visit may be randomised to the study. Patients who fail a screening visit(s) may not be randomised to the study.

**7.3 Baseline / Randomisation Visit(s)**

The baseline/randomisation visit must occur no more than 4 weeks after the patient has successfully passed screening. The assessments will be conducted virtually by a research assistant.

The data as detailed below and in the study procedures table (Section 4.4) must be collected and all required procedures performed during the baseline/randomisation visit.

- Concomitant medications
- Vital signs (Blood pressure & heart rate) (Retrospective review of most recent reported measurements)
- Height, weight, BMI
- Patient Activation Measure (PAM-13)
- Clinical Frailty Scale
- Physical Health Questionnaire-4 (PHQ-4)
- Fatigue severity score (Chalder Fatigue Questionnaire)
- Work and social adjustment scale (WSAS Questionnaire)
- Functional capacity (sit-to-stand 60 to assess lower limb function)
- Global Physical Activity Questionnaire (GPAQ)
- Are you meeting current physical activity guidelines?
- Quality of life (KDQOL-36 and EQ5D-5L)
- Routine blood tests – Creatinine, Haemoglobin, CRP, IL-6, Albumin

**7.3.1 Treatment Procedures**

Participants will be randomised to receive either the Kidney Beam digital health intervention platform or will be randomised to waitlist for 12 weeks. At the end of the 12 weeks, all participants will be re-assessed before the waitlist participants are then offered the intervention. Participants in the non-waitlist group will be followed up after 6 months.

**7.3.2 Fatigue**

The Chalder Fatigue Questionnaire is an 11-item questionnaire measuring the severity of physical and mental fatigue on two separate subscales (47). Seven items represent physical fatigue (items 1-7) and 4 represent mental fatigue (items 8-11). Each item is scored; better than usual (0), no worse than usual (1), worse than usual (2) and much worse than usual (3). The ratings of items are added together to calculate the total score (range = 0-33) where high scores represent greater levels of fatigue. This questionnaire has previously been used as a measure of fatigue in HD patients as well as other long-term medical conditions. Internal reliability of the total fatigue score is high among renal patients (>.90).

**7.3.3 *Functional impairment and physical activity***

The Work and Social Adjustment Scale (WSAS) is a valid and reliable self-report scale of functional impairment attributable to an identified problem (in this case fatigue) (48,49). The scale consists of five items that correspond to impairment in work, home management, social activities, private leisure activities and relationships. Each item is rated on a 9-point scale ranging from 0 (not at all a problem) to 8 (very seriously impaired), with high scores indicating greater impairment (α=0 .90).

7.3.4 *Physical function and physical activity*

*Sit-to-Stand 60 Seconds (STS60):* We will also use the STS60 to provide a measure of muscle endurance, another aspect of physical performance (50,51). The STS60 is a commonly used and simple assessment of muscle endurance, an aspect of physical performance that directly influences ability to carry out daily tasks. This test requires the patient to stand up from a chair and sit back down again and counts the amount of repetitions completed in 60 seconds. Participants will be asked if they are meeting current physical activity guidelines with the following 3 questions: 1) Do you do strength training on 2 or more days of the week? 2) Do you do 150 mins or more of moderate intensity physical activity per week? 3) Do you do 75 mins or more of vigorous physical activity per week?

***7.3.5 Patient Activation Measure (PAM-13)***

The PAM-13 is a validated tool of 13 questions which assesses a patient’s knowledge, skills and confidence in managing their own health. The PAM-13 has demonstrated good internal consistency as well as adequate reliability and validity (52,53). Answers are weighted and combined to provide a score on a scale from 0 to 100. A score is generated where participants have answered ≥10 questions. The PAM allows respondents to be categorized into one of four levels with lower levels indicating low activation and higher levels indicating high activation): Level 1 (<47.0), disengagement and disbelief about one’s own role in self-management; Level 2 (47.1–55.1), increasing awareness, confidence, and knowledge in self-management tasks; Level 3 (55.2–67), readiness and taking action; and Level 4 (>67.1), sustainment.

***7.3.6 Clinical Frailty Score***

The Clinical Frailty Scale (CFS), a risk stratification tool which grades  frailty level from 1 (very fit) to 9 (terminally ill) using the standardised qualifiers, and has been validated for use in a range of CKD populations, will be used to identify the frailty status of participants at baseline (64).

***7.3.7 Physical Health Questionnaire-4 (PHQ-4)***

The Patient-Health Questionnaire-4 (PHQ-4) is an ultra-brief screener for depression and anxiety (Kroenke, et al 2009). The measure has 4 items, two each from the PHQ-2 and GAD-2. This brief screener has good internal reliability and construct validity. A PHQ-4 total score of ≥6 or symptom score ≥3 on the depression or anxiety items of PHQ-4 represent likely clinical levels of depression or anxiety (Löwe et al, 2010)

***7.3.8 Healthcare Utilisation***

A healthcare utilisation questionnaire will be administered in order to assess the extent of healthcare usage over the previous 3 months including but not limited to inpatient services, outpatient services, community and general practitioner services and the emergency services. This will provide a basis on which the costs associated with setting up and maintaining Kidney BEAM and its content can be compared to the potential benefits such as keeping patients fitter, more active, out of hospital for longer and in some cases, able to work.

**7.4 Kidney Beam Digital Health Intervention Platform**

Following the baseline assessment, those participants randomised to start immediately will be offered the Kidney Beam digital health intervention platform. Kidney Beam [https://beamfeelgood.com/home](about:blank) is a wellbeing digital health intervention platform that offers patients living with kidney disease a way to improve their physical activity and boost their mental health through live and on demand movement classes and expert educational videos, while remaining in their own home. Kidney Beam is led by specialist kidney professionals, including renal physiotherapists and renal counsellors, from a number of different NHS Trusts and backgrounds, as well as people living with kidney disease. The resource is aimed at anyone over 18, with any ability, any kidney condition, and at any stage of kidney disease.

Each participant will be encouraged to attend twice weekly virtual renal rehabilitation sessions led by a renal physiotherapist and a band 4 technical instructor in a group setting. This will be provided as a rolling 12-week programme and will include disease-specific education sessions. The sessions will be modelled on the King’s College Hospital Renal Rehabilitation programme.

Each participant will be encouraged to accumulate 150 mins/week of moderate intensity aerobic activity or 75 mins/week of vigorous activity, and to do some muscle resistance training on 2 days of the week. As well as partaking in renal rehabilitation classes, participants will be encouraged to engage with any other activities offered on Kidney Beam. This will be recorded on the platform and will be reported for each participant. At the end of the kick-start 12-week programme, participants will be encouraged to maintain and self-manage their activity via Kidney Beam.

**7.5 Week 12 Appointment**

The week 12 appointment must occur 12 weeks (+4 weeks) after the patient has had their baseline appointment.

The data as detailed below and in the Trial Flowchart must be collected and all required procedures performed during the 12-week appointment.

- Concomitant medications
- Vital signs (Blood pressure & heart rate) (Retrospective review of most recent reported measurements)
- Height, weight, BMI
- Patient Activation Measure (PAM)
- Clinical Frailty Score
- Physical Health Questionnaire-4 (PHQ-4)
- Fatigue severity score (Chalder Fatigue Questionnaire)
- Functional Impairment (WSAS Questionnaire)
- Functional capacity (sit-to-stand 60 to assess lower limb function)
- Global Physical Activity Questionnaire (GPAQ)
- Are you meeting current physical activity guidelines?
- Quality of life (KDQOL-36 and EQ5D-5L)

**7.6 6-month Follow-up**

The 6-month follow-up must occur 6 months (+4 weeks) after the participant has had their 12-week appointment. This will be conducted virtually via the Kidney Beam platform

- Quality of life (KDQOL-36 and EQ5D-5L)
- Kidney Beam platform metrics (physical activity mins and engagement)

**7.7** **Qualitative Exploration of Patient Experience and Acceptability**

Semi-structured interviews will be used to gather qualitative data as they offer an open and flexible method for exploring the participants’ individual experiences in-depth.

Interviews will occur at two time points:

- At the end of the internal pilot phase to further understand the acceptability of the platform and to contextualise and expand upon the feasibility outcomes (‘pilot interviews’).
- On completion or withdrawal from the trial to explore the impact of the intervention upon participants’ experiences of living with kidney disease, to contextualise the results of the trial and to understand the potential longer-term acceptability of using BEAM (‘final interviews’).

If time and resources permit, further exploration of maintenance and factor influencing the sustainability of the programme will be undertaken at the long-term follow-up time point.

For all interviews the inclusion and exclusion criteria for the qualitative component will mirror that of the main trial, although participants who are unable to speak English will be excluded due to challenges relating to interpretation and translation. To reduce potential problems with recall bias, participants will be interviewed as close to the end of the pilot phase, or the completion of the full trial.

**Sampling and sample size**

Maximum variation sampling will initially be used to ensure that participants are purposefully different from each other (54). For the pilot interviews primary importance will be given sampling participants who have had a diverse range of engagement with the BEAM platform to provide greater insight into its acceptability. For final interviews, primary importance will be given to ensuring good representation of participants living with different stages of CKD and/or receiving different forms of renal replacement therapy from both arms of the trial. For all interviews, other important characteristics (e.g. age, gender, ethnicity) will be monitored on an ongoing basis as recruitment and analysis progresses (55).

For the pilot interviews, up to 15 participants from the intervention arm will be recruited, although information power will be assessed throughout and the final sample size determined by the quality of the data and its ability to address the qualitaitve aims (56). For the final interviews, up to 30 participants will be recruited in total, from each arm of the trial. Again, data collection will cease at the point where information power is achieved.

Topic guides for each interview will be developed in advance of the study in partnership with the trial steering committee and PPI representatives, guided by the RE-AIM framework, which is designed to enhance the quality, speed, and public health impact of efforts to translate research into practice (57).The first three interviews at each phase will act as pilots (but will be included within the overall analyses). Interviews will be conducted by an experienced qualitative researcher at an appointment separate from the other trial assessments, via telephone or video link. Family members or other relatives may be present during the interview if the participant wishes.

During the interviews, the researcher will introduce the process, explaining the background, how the interview will proceed, and details of audio-recording and note-taking. Patients will be reassured about the preservation of their anonymity and confidentiality as well as being given the opportunity to ask questions before audio-recording commences. Interviews are expected to last approximately 60 minutes. At the end of each interview, participants will be given another opportunity to ask questions, or seek clarification and the voluntary nature of their participation will be re-iterated. Should any participant request further information or highlight any concerns during the interview then these will be discussed with the Principal Investigator at their recruiting site, for appropriate onward referral as required. All participants will be made fully aware of the contact details for the research team, should they have any concerns following the interview.

The recorded interviews will be professionally transcribed verbatim. Transcripts will not include any-identifying information; individual’s names and personal details will not be included in the completed transcripts but will be recorded and stored separately. Similarly, any written notes taken during the interviews will not include any identifiable personal data.

Thematic analysis of all sets of interviews will be conducted alongside data collection according to the methods outlined by Braun and Clarke (2006) (58). Briefly, researchers with qualitative research experience will independently familiarise themselves with the data and generate initial codes inductively from the data. These codes will then be collated into initial themes, which will be reviewed by the multidisciplinary research team and the PPI group, until they are refined. The researchers will move back and forth between stages as new themes are generated and relationships recognised. Participants' narratives across interviews will be compared and contrasted. NVivo (QSR International) software will be used to manage the qualitative data and to facilitate analysis.

**Mixed methods analysis, and implementation of findings at the end of the pilot phase**

Following separate qualitative and quantitative data analyses the results will be merged into two ‘joint displays’, which combine the findings in a tabulated form, will be used to assess the ways in which the qualitative and quantitative data sets agreed (confirmed), complemented (offered an expanded explanation) or contradicted each other (59).

The first joint display will address the feasibility and acceptability of the BEAM platform and the trial procedures (using data from the internal pilot stage). This will be used to inform adaptations to the intervention and trial procedures, ahead of progression to the full trial.

An implementation group will be set up to enable rapid application of the qualitative findings in the main trial. This group will comprise the chief investigator, co-investigators, PPI representatives and representatives from participating centres. A meeting via videoconferencing will take place at the end of the pilot phase to enable the qualitative researchers to feedback their findings to the group so that recommendations can be implemented into the main trial.

The second joint display will combine data from the final interviews (relating to participant perception and experience) with trial data to facilitate a deeper understanding of the effects (or lack of effect) of the BEAM platform (using) (60).

**7.8 Qualitative Sub-Study- Digital Inclusion**

A qualitative sub-study will be conducted to explore aspects of digital inclusion and digital health literacy in participants who have declined participation to the study, or who have not engaged with the digital platform. This will help to refine the platform and ensure that this is in line with service users, as well as addressing potential inequalities in access to a digital health platform.

It is understood from the literature that there are some sections of the population who are more likely to be excluded. This includes:

- Older people
- People in lower income groups
- People without a job
- People in social housing
- People with fewer educational qualifications
- People living in rural areas

Individual interviews will be conducted, with up to 20 participants. Data collection will cease at the point where data saturation is achieved. Purposive sampling will be used to ensure varied experiences and viewpoints are represented, for example, those at different stages of CKD, representative of different ethnic backgrounds, gender, age and study regions.

Appropriate topic guides will be developed in advance of the study. Questions will be discussed within the project team and trial steering group. Participant information sheets will be sent via post, email or provided face to face. Interviews will be conducted by the experienced qualitative team, and supported by an individual undertaking a funded digital inclusion fellowship. Interviews will be conducted over the telephone, online, or in person if able, at an appointment separate from other trial assessments or discussions. Informed consent will be taken digitally prior to the discussion, or if access to the internet is not possible consent forms will be sent via post with a stamped addressed envelope for the participant to return. Individuals participating in this digital inclusion sub-study will be offered £20 to re-imburse their time to meet with the qualitative interviewer.

A questionnaire to collect demographic information will be completed by each participant at the start of the individual semi-structured interview. This will provide further demographic information about the participants in order to further understand potential barriers to digital inclusion. This information will be used to guide the sampling and to help explore the data during analysis.

The recorded interviews will be professionally transcribed verbatim. Transcripts will not include any identifying information; individual’s names and personal details will not be included in the completed transcripts but will be recorded and stored separately. Similarly, any written notes taken during the interviews will not include any identifiable personal data. Sampling will be as per sampling framework in Section 7.7

**Analysis and implementation of findings**

Analysis will be completed according to the methods outlined by Braun and Clarke, as outlined in the main qualitative study in 7.7.

**7.9 Expected Duration of Trial**

18 months (6 weeks set-up, 9 months recruitment, 3 months follow-up after intervention, 6 months long-term follow-up, 3 months analysis and write-up for main study).

| The end of the trial will be defined as last patient assessed. |
| --- |

**8. Laboratory Tests**

Before starting the study, the principal investigator at each participating site will supply the Sponsor with a list of the normal ranges and units of measurements for the applicable parameters measured in the study. Any changes to these ranges and units must also be notified to the Sponsor.

All laboratory parameters performed and assessed in local laboratories at each site will be reviewed at each visit assessment.

**Laboratory Parameters:**

*Biochemistry*

- Creatinine, eGFR, C-reactive Protein (CRP), Hemoglobin (Hb), IL-6, Albumin

### 9. Assessment of Safety

### 9.1 Specification, Timing and Recording of Safety Parameters.

### Serious adverse events will be identified and reported to the sponsor. In addition, episodes of hospitalisation and other infection episodes will be documented.

###

### 10. Statistics

### 10.1 Sample Size

The choice of endpoint measures to be studied have been chosen to provide clinical relevance. Studies that have evaluated the clinically meaningful change in MCS of the KDQOL-36 HRQoL questionnaire in people living with kidney disease (refs) indicate that a clinically meaningful improvement in MCS is 3AUs. An estimated sample size of 106 participants in each group (total N = 212) based on an MCS with a mean of 45, SD 10 and correlation between repeated measures of 0.7, would allow a clinically meaningful difference of 3AUs to be detected at 80% power and 5% alpha. We will recruit 304 participants in total to allow for potential dropouts of 30%.

**10.2 Randomisation**

Participants will be randomised using an approach based on randomly varying block sizes. The randomisation will be stratified by a single binary variable, defined by whether or not patients have diabetes, or not.

**10.3 Analysis**

Baseline demographics of the patients will be described using summary statistics. Continuous variables will be summarised using the mean and SD if approximately normally distributed. Continuous variables that are not normally distributed will be summarised using the median and interquartile range. Categorical variables will be summarised using frequencies and percentages. Comparisons will be made between the demographic variables of the control and intervention groups using t-tests, median tests and chi-square tests as appropriate.

Repeated measures analysis of variance will be employed to investigate the change from baseline to 12 weeks follow-up between the control and intervention groups for the primary outcome variable (MCS) and other parametric outcome measures. If the assumptions for parametric testing are not met, the equivalent non-parametric equivalent tests will be performed.

As this is the first study of this novel intervention, exploratory data analysis will also be undertaken to investigate possible associations between outcomes in order to frame future research questions.

Adverse events will be collected and analysed descriptively.

Statistical significance will be set at 5% and tests will be two-sided. The analysis will be undertaken in SPSS version 26.

### 11. Trial Steering Committee

| The Trial Steering Committee (TSC) will meet prior to commencement of the study, after Ethics approval, via teleconference, and thereafter every 6 months to assess study conduct and recruitment. |
| --- |

### 12. Data Monitoring Committee

The IDMC will include at least 3 members to review data on safety, including SAEs, and the primary outcome measures, and will advise the steering committee on acceptable continuation of the study, or whether the study should be stopped. The IDMC will meet via teleconference at a minimum of every4 months (and more frequently if deemed necessary).

### 13. Ethics & Regulatory Approvals

| The trial will be conducted in compliance with the principles of the Declaration of Helsinki (1996), the principles of GCP and in accordance with all applicable regulatory requirements including but not limited to the UK Policy Framework for Health and Social care.  This protocol and related documents will be submitted for review to *** Research Ethics Committee (REC) and the Health Research Authority (HRA). The trial will also need confirmation of Capacity and Capability from recruiting sites before recruitment can commence at NHS Trust.  The Chief Investigator will submit a final report at conclusion of the trial to the REC and the HRA. Progress reports will be sent to the REC annually on the date of REC Favourable opinion followed by the Declaration of the end of study when the study ends and final report (either 12 months after the study ends or at the same time of the Declaration of the end of the study). |
| --- |

### 14. Data Handling

| The Chief Investigator will act as custodian for the trial data. The following guidelines will be strictly adhered to:  Patient data will be pseudonymised*.*   - All pseudonymised data will be stored on a password protected computer. |
| --- |

### 15. Database

| All data will be collected centrally via BEAM or KCH. An independent statistician will analyse the data. |
| --- |

### 16. Insurance / Indemnity

KCH will provide NHS indemnity cover for negligent harm, as appropriate and is not in the position to indemnify for non-negligent harm. NHS indemnity arrangements do not extend to non-negligent harm and NHS bodies cannot purchase commercial insurance for this purpose; it cannot give advance undertaking to pay compensation when there is no negligence attributable to their vicarious liability. The Trust will only extend NHS indemnity cover for negligent harm to its employees, both substantive and honorary, conducting research studies that have been approved by the R&I Office. The Trust cannot accept liability for any activity that has not been properly registered and Trust approved. Potential claims should be reported immediately to the KCH R & I Office.

### 17. Financial Aspects

| Funding to conduct the trial is provided by Kidney Research UK. The trial protocol was also peer reviewed by Kidney Research UK. |
| --- |

#

# 18. Publication policy

It is intended that the results of the study will be reported and disseminated at conferences and in peer-reviewed scientific journals. All proposed publications will be discussed with and reviewed by the Sponsor prior to publishing other than those presented at scientific forums/meetings.**19.** **Project Costs**

| **Item** | **Description** | **Cost** |
| --- | --- | --- |
|  |  |  |
| BEAM platform access for the UK | This covers a license to provide access to Kidney Beam to anyone living with kidney disease in the UK for a 12-month period.  Kidney Beam offers its members a growing library of on-demand exercise and educational classes as well as a timetable of weekly live classes hosted by exercise specialists either trained in or living with kidney disease.  Alongside these classes, Beam offers support through online community groups and a variety of science-backed, behavioural change features to help motivate people to move more for the good of their physical and mental health. | £96,000 |
| 1.0 WTE band 6 Research Assistant 12 months | Blinded research assistant who will collect baseline and 12-week data via virtual platform Attend Anywhere. | £56,150 |
| Independent project statistician | Sample size calculations, all statistical analyses for project (60 hours) | £3402 |
| Independent health economist | Analysis and interpretation of health utilisation data and EQ5D-5L QALY data (60 hours) | £3820 |
| Consumables | Web cams, microphones for teaching classes and Transcription software | £500 |
| **Total cost** |  | **£159,872** |

###

### 20. Signatures

______________________________________ _________________________

Chief Investigator Date

*Dr Sharlene Greenwood*

______________________________________ _________________________

Statistician Date

*Professor Jackie Campbell*

______________________________________ _________________________

### *Local Principal Investigator Date*

(PRINTED NAME & SIGNATURE)

### 21. References

1. Brown SA, Tyrer FC, Clarke AL, Lloyd-Davies LH, Stein AG, Tarrant C, et al. Symptom burden in patients with chronic kidney disease not requiring renal replacement therapy. Clinical Kidney Journal. 2017;10(6):788-96.
2. Brown SA, Tyrer FC, Clarke AL, Lloyd-Davies LH, Stein AG, Tarrant C, et al. Symptom burden in patients with chronic kidney disease not requiring renal replacement therapy. Clinical Kidney Journal. 2017;10(6):788-96.
3. Knight EL, Ofsthun N, Teng M, Lazarus JM, Curhan GC. The association between mental health, physical function, and hemodialysis mortality. Kidney Int. 2003;63(5):1843-51. doi: 10.046/j.523-755.2003.00931.x.
4. Lowrie EG, Curtin RB, LePain N, Schatell D. Medical outcomes study short form-36: a consistent and powerful predictor of morbidity and mortality in dialysis patients. Am J Kidney Dis. 2003;41(6):1286-92. doi: 10.016/s0272-6386(03)00361-5.
5. Heiwe S, Jacobson SH. Exercise training for adults with chronic kidney disease. Cochrane Database of Systematic Reviews. 2011;Oct 5(10):CD003236.
6. Heiwe S, Jacobson SH. Exercise training in adults with CKD: a systematic review and meta-analysis. American journal of kidney diseases : the official journal of the National Kidney Foundation. 2014;64(3):383-93.
7. Cheema BS, Chan D, Fahey P, Atlantis E. Effect of progressive resistance training on measures of skeletal muscle hypertrophy, muscular strength and health related quality of life in patients with chronic kidney disease: A systematic review and meta-analysis. Sports Med. 2014;44(8):1125-38.
8. Segura-Orti E. Exercise in hemodialysis patients: A literature systematic review. Nefrologia. 2010;30(2):236-24.
9. Smart N, Steele M. Exercise raining in haemodialysis patients: A systematic review and meta-analysis. Nephrology (Carlton). 2011;16(7):626-32.
10. Salhab N, Karavetian M, Kooman J, Fiaccadori E, El Khoury CF. Effects of intradialytic aerobic exercise on hemodialysis patients: a systematic review and meta-analysis. J Nephrol. 2019;32(4):549-66. doi: 10.1007/s40620-018-00565-z. Epub 2019 Jan 18.
11. Sheng K, Zhang P, Chen L, Cheng J, Wu C, Chen J. Intradialytic exercise in hemodialysis patients: a systematic review and meta-analysis. Am J Nephrol. 2014;40(5):478-90. doi: 10.1159/000368722. Epub 2014 Dec 9.
12. Chung YC, Yeh ML, Liu YM. Effects of intradialytic exercise on the physical function, depression and quality of life for haemodialysis patients: a systematic review and meta-analysis of randomised controlled trials. J Clin Nurs. 2017;26(13-14):1801-13. doi: 10.111/jocn.13514. Epub 2017 Mar 20.
13. Pu J, Jiang Z, Wu W, Li L, Zhang L, Li Y, et al. Efficacy and safety of intradialytic exercise in haemodialysis patients: a systematic review and meta-analysis. BMJ Open. 2019;9(1):e020633. doi: 10.1136/bmjopen-2017-.
14. Young HML, March DS, Graham-Brown MPM, Jones AW, Curtis F, Grantham CS, et al. Effects of intradialytic cycling exercise on exercise capacity, quality of life, physical function and cardiovascular measures in adult haemodialysis patients: a systematic review and meta-analysis. Nephrol Dial Transplant. 2018;33(8):1436-45.
15. Huang M, Lv A, Wang J, Xu N, Ma G, Zhai Z, et al. Exercise Training and Outcomes in Hemodialysis Patients: Systematic Review and Meta-Analysis. Am J Nephrol. 2019;50(4):240-54. doi: 10.1159/000502447. Epub 2019 Aug 27.
16. Zhao QG, Zhang HR, Wen X, Wang Y, Chen XM, Chen N, et al. Exercise interventions on patients with end-stage renal disease: a systematic review. Clin Rehabil. 2019;33(2):147-56. doi: 10.1177/0269215518817083.
17. Clarkson MJ, Bennett PN, Fraser SF, Warmington SA. Exercise interventions for improving objective physical function in patients with end-stage kidney disease on dialysis: a systematic review and meta-analysis. Am J Physiol Renal Physiol. 2019;316(5):F856-F72. doi: 10.1152/ajprenal.00317.2018. Epub 2019 Feb 13.
18. Gomes Neto M, de Lacerda FFR, Lopes AA, Martinez BP, Saquetto MB. Intradialytic exercise training modalities on physical functioning and health-related quality of life in patients undergoing maintenance hemodialysis: systematic review and meta-analysis. Clin Rehabil. 2018;32(9):1189-202.
19. Pawlikowska T, Chalder T, Hirsch S, et al.: Population based study of fatigue and psychological distress. Bmj. 1994, 308:763-766.
20. David A, Pelosi A, McDonald E, et al.: Tired, weak, or in need of rest: fatigue among general practice attenders. Bmj. 1990, 301:1199-1202.
21. Artom M, Moss-Morris R, Caskey F, Chilcot J: Fatigue in advanced kidney disease. Kidney international. 2014; 6(83), 497-505. DOI:<https://doi.org/10.1038/ki.2014.86>
22. Bossola M, Vulpio C, Tazza L: Fatigue in chronic dialysis patients. Seminars in Dialysis: 2011; 24(5),550-555.  <https://doi.org/10.1111/j.1525-139X.2011.00956.x>
23. Jhamb M, Pike F, Ramer S, et al.: Impact of fatigue on outcomes in the hemodialysis (HEMO) study. American journal of nephrology. 2011, 33:515-523.
24. Neto JFR, Ferraz MB, Cendoroglo M, et al.: Quality of life at the initiation of maintenance dialysis treatment - A comparison between the SF-36 and the KDQ questionnaires. Quality of Life Research. 2000, 9:101-107.
25. Koyama H, Fukuda S, Shoji T, et al.: Fatigue is a predictor for cardiovascular outcomes in patients undergoing hemodialysis. Clinical Journal of the American Society of Nephrology. 2010, 5:659-666.
26. Brown SA, Tyrer FC, Clarke AL, Lloyd-Davies LH, Stein AG, Tarrant C, et al. Symptom burden in patients with chronic kidney disease not requiring renal replacement therapy. Clinical Kidney Journal. 2017;10(6):788-96.
27. Knight EL, Ofsthun N, Teng M, Lazarus JM, Curhan GC. The association between mental health, physical function, and hemodialysis mortality. Kidney Int. 2003;63(5):1843-51. doi: 10.046/j.523-755.2003.00931.x.
28. Jhamb M, Argyropoulos C, Steel JL, et al.: Correlates and outcomes of fatigue among incident dialysis patients. Clinical Journal of the American Society of Nephrology. 2009, 4:1779-1786.
29. Greenwood SA, Koufaki P, Rush R, Macdougall IC, Mercer TH; British Renal Society Rehabilitation Network. Exercise counselling practices for patients with chronic kidney disease in the UK: a renal multidisciplinary team perspective. Nephron Clin Pract. 2014;128(1-2):67-72. doi: 10.1159/000363453. Epub 2014 Oct 29. PMID: 25358965.
30. Banks J, Bell A, Chilcot J, Currie S, Farrington K, Greenwood S et al. Kidney disease and mental health. Statement of intent from Centre for Mental Health and Kidney Research UK. 2020. Available at: <https://kidneyresearchuk.org/about-us/position-statements/statement-on-mental-health/>
31. Lippi, G., Sanchis-Gomar, F., Henry, B. Coronavirus disease 2019 (COVID-19): the portrait of a perfect storm. Annals of Translational Medicine. 2020, (8), 7. doi: 10.21037/atm.2020.03.157
32. British Renal Society. COVID-19: PPE and use of masks by dialysis patients. 2020. Available at: <https://britishrenal.org/ppe-and-use-of-masks-by-dialysis-patients/>
33. Jhamb, M., McNulty, M.L., Ingalsbe, G. *et al.* Knowledge, barriers and facilitators of exercise in dialysis patients: a qualitative study of patients, staff and nephrologists. *BMC Nephrol* 17, 2016; 192 <https://doi.org/10.1186/s12882-016-0399-z>
34. Clarke A, Young H, Hull K, Hudson N, Burton J, Smith A. Motivations and barriers to exercise in chronic kidney disease: a qualitative study, Nephrology Dialysis Transplantation. 205 (30), Issue 11, 1885–1892, <https://doi.org/10.1093/ndt/gfv208>
35. Maruthappu M, Sood HS, Keogh B. The NHS Five Year Forward View: transforming care. *Br J Gen Pract*. 2014;64(629):635. doi:10.3399/bjgp14X682897
36. Lord Carter. The Carter Report. Operational productivity and performance in English NHS acute hospitals: Unwarranted variations. Department of Health and Social Care. 2015. Available at: <https://www.gov.uk/government/publications/productivity-in-nhs-hospitals>
37. Eldridge, S.M., et al., CONSORT 2010 statement: extension to randomised pilot and feasibility trials. Pilot and feasibility studies, 2016. 2(1): p. 64.
38. Avery, K.N., et al., Informing efficient randomised controlled trials: exploration of challenges in developing progression criteria for internal pilot studies. BMJ open, 2017. 7(2): p. e013537.
39. Harper, L., et al., Treatment of fatigue with physical activity and behavioural change support in vasculitis: study protocol for an open-label randomised controlled feasibility study. BMJ open, 2018. 8(10): p. e023769.
40. Young HML, Goodliffe S, Madhani M, et al. Co-producing Progression Criteria for Feasibility Studies: A Partnership between Patient Contributors, Clinicians and Researchers. *Int J Environ Res Public Health*. 2019;16(19):3756. Published 2019 Oct 6. doi:10.3390/ijerph16193756
41. Williamson EJ, Walker AJ, Bhaskaran K, Bacon S, Bates C, Morton CE, Curtis HJ, Mehrkar A, et al. Factors associated with COVID-19-related death using OpenSAFELY. Nature. 2020 Aug;584(7821):430-436. doi: 10.1038/s41586-020-2521-4. Epub 2020 Jul 8. PMID: 32640463.
42. Stenvinkel P, Carrero JJ, von Walden F, Ikizler TA, Nader GA. Muscle wasting in end-stage renal disease promulgates premature death: established, emerging and potential novel treatment strategies. Nephrol Dial Transplant. 2016 Jul;31(7):1070-7. doi: 10.1093/ndt/gfv122. Epub 2015 Apr 24. PMID: 25910496.
43. Gabarre P, Dumas G, Dupont T, Darmon M, Azoulay E, Zafrani L. Acute kidney injury in critically ill patients with COVID-19. Intensive Care Med. 2020 Jul;46(7):1339-1348. doi: 10.1007/s00134-020-06153-9. Epub 2020 Jun 12. PMID: 32533197; PMCID: PMC7290076.
44. Frederikus A. Klok, Gudula, J.A.M. Boon, Stefano Barco, Matthias Endres, J.J. Miranda Geelhoed, Samuel Knauss, Spencer A. Rezek, Martijn A. Spruit, Jörg Vehreschild, Bob Siegerink. The Post-COVID-19 Functional Status scale: a tool to measure functional status over time after COVID-19 European Respiratory Journal 2020 56: 2001494; DOI: 10.1183/13993003.01494-2020
45. Hamilton AJ, Caskey FJ, Casula A, Inward CD, Ben-Shlomo Y. Associations with Wellbeing and Medication Adherence in Young Adults Receiving Kidney Replacement Therapy. Clinical Journal of the American Society of Nephrology : CJASN. 2018 Nov;13(11):1669-1679. DOI: 10.2215/cjn.024502.
46. Nixon, A.C., Bampouras, T.M., Pendleton, N., Mitra, S. and Dhaygude, A.P. (2019) 'Diagnostic accuracy of frailty screening methods in advanced chronic kidney disease', *Nephron,*141(3), pp. 147-155.
47. Chalder T, Berelowitz G, Pawlikowska T, *et al.*Development of a fatigue scale. J Psychosom. 1993; Res, 37, pp. 147-153
48. Mundt JC, Marks IM, Shear MK, Greist JH. The Work and Social Adjustment Scale: a simple measure of impairment in functioning. Br J Psychiatry. 2002;180:461–4.
49. Mataix-Cols D, Cowley AJ, Hankins M, Schneider A, Bachofen M, Kenwright M, et al. Reliability and validity of the Work and Social Adjustment Scale in phobic disorders. Comprehensive Psychiatry. 46:223–8.
50. Wilkinson T.J., Xenophontos S, Gould D, Vogt B, Viana J, Smith A, Watson E.L. Test–retest reliability, validation, and “minimal detectable change” scores for frequently reported tests of objective physical function in patients with non-dialysis chronic kidney disease, Physiotherapy Theory and Practice. 2019; 35:6, 565-576, DOI: [10.1080/09593985.2018.1455249](https://doi.org/10.1080/09593985.2018.1455249)
51. Wilkinson TJ, Watson E, Xenophontos S, Gould D, Smith A. The “Minimum Clinically Important Difference” in Frequently Reported Objective Physical Function Tests After a 12-Week Renal Rehabilitation Exercise Intervention in Nondialysis Chronic Kidney Disease, American Journal of Physical Medicine & Rehabilitation: 2019 ; (98) 6 - p 431-437 doi: 10.1097/PHM.000000000000108.
52. Hibbard JH, Mahoney ER, Stockard J, Tusler M. Development and testing of a short form of the patient activation measure. Health Serv Res 2005; 40:1918-1930
53. Hibbard JH, Stockard J, Mahoney ER, Tusler M. Development of the Patient Activation Measure (PAM): conceptualizing and measuring activation in patients and consumers. Health Serv Res 2004; 39:1005-102.
54. Robinson, O.C. 'Sampling in interview-based qualitative research: A theoretical and practical guide', *Qualitative Research in Psychology.* 2014;11(1), pp. 25-41. <https://doi.org/10.1080/14780887.2013.801543>
55. Ritchie, J., Lewis, J., Nicholls, C. M., & Ormston, R. (Eds.). Qualitative research practice: A guide for social science students and researchers. Sage. 2013; volume 2
56. Malterud K, Siersma VD, Guassora AD. Sample Size in Qualitative Interview Studies: Guided by Information Power. Qual Health Res. 2016;26(13):1753-60.  doi: 10.1177/1049732315617444.
57. RE-AIM.RE-AIM Improving public health relevance and population health impact [online]. Cited 24^th^ Sept 2020. Available from: [www.re-aim.org/](http://www.re-aim.org/)
58. Braun, V. and Clarke, V. Using thematic analysis in psychology. *Qualitative Research in Psychology.* 2006 3 (2). 77-101. doi: 10.1191/1478088706qp063oa
59. O'Cathain, A., Murphy, E. and Nicholl, J. (2010) 'Three techniques for integrating data in mixed methods studies', *British Medical Journal.* 2010; 341, pp. c4587. doi: <https://doi.org/10.1136/bmj.c4587>
60. Richards DA, Bazeley P, Borglin G, et al. Integrating quantitative and qualitative data and findings when undertaking randomised controlled trials. *BMJ Open* 2019;9:e032081. doi:10.1136/ bmjopen-2019-032081
