# Supplementary material for: A Digital Inclusion Intervention to Improve Access to a Digital Health Intervention Among Digitally Excluded Adults: Mixed Methods Pilot Randomized Controlled Trial
Source: JMIR Form Res. 2026 Apr 16;10:e91438. doi: 10.2196/91438 (PMC13085982; doi:10.2196/91438)
Supplement: Multimedia Appendix 3 [file formative-v10-e91438-s003.docx]

Supplementary material

Table S1. Inclusion and exclusion criteria for the study.

| Inclusion criteria | Exclusion criteria |
| --- | --- |
| - Patients with established CKD - Aged 18 years+ - Patients who are naïve to Kidney Beam intervention and who have not participated in a structured exercise programme in the prior 3 months - Patients that do not have an electronic device. - Patients that have self-reported low confidence using electronic devices. - Patients that do not have Wi-Fi access or data. | - Weight < 50kg - Self-reported participation in a structured exercise programme or kidney beam digital health intervention platform within previous 3 months - Active infection - Uncontrolled arrhythmias - Unstable angina or heart attack within the 3 months - Persistent uncontrolled hypertension (systolic blood pressure >180 mm Hg or diastolic blood pressure >110 mm Hg) - Recent (within the last 3 months) stroke or transient ischaemic attack - Receiving palliative care for advanced terminal cancer - Patients with peripheral vascular or musculoskeletal disease, who the investigator deems unable to carry out a physical activity intervention. - Any other health condition considered by the local Principal Investigator in which exercise therapy will be contraindicated. - Insufficient understanding of the trial |

Table S2. Baseline demographic and clinical characteristics of the trial participants

|  | Total (n=40) | EX-TAB (n=21) | Control (n=19) |
| --- | --- | --- | --- |
| Age, years | 66.5 (59-73.75) | 68 (58-74.5) | 66 (59-73) |
| **Sex** | | | |
| Male | 20 (50%) | 9 (43%) | 11 (58%) |
| Female | 20 (50%) | 12 (57%) | 8 (42%) |
| **Ethnicity** | | | |
| Black | 19 (47.5%) | 10 (48%) | 9 (47%) |
| White | 15 (37.5%) | 8 (38%) | 7 (37%) |
| Asian | 6 (15%) | 3 (14%) | 3 (16%) |
| BMI, kg/m^2^ | N=38 | N=20 | N=18 |
| Median (IQR) | 30.2 (6.7) | 28.9 (5.3) | 31.7 (7.9) |
| Digital Health Literacy Screening Tool | 3 (1-5) | 4 (2-5) | 3 (1-5) |
| **Smoking** | | | |
| Current | 3 (7.5%) | 2 (9.5%) | 1 (5.3%) |
| Former | 11 (27.5%) | 9 (42.9%) | 2 (10.5%) |
| Never | 26 (65%) | 10 (47.6%) | 16 (84.2%) |
| **Alcohol consumption** | | | |
| More than recommended | 1 (2.5%) | 0 (0%) | 1 (5.3%) |
| Less than recommended | 14 (35%) | 7 (33.3%) | 7 (36.8%) |
| Non-drinker | 25 (62.5%) | 14 (66.7%) | 11 (57.9%) |
| Blood pressure, mm Hg | N=36 | N=20 | N=16 |
| Systolic | 129 (22) | 124 (23) | 135 (19) |
| Diastolic | 74 (12) | 70 (13) | 78 (10) |
| Resting heart rate, beats per minute | N=32 | N=18 | N=14 |
| Mean (SD) | 73 (12) | 73 (12) | 72 (12) |
| **Comorbidities** | | | |
| Cerebrovascular accident | 4 (10%) | 2 (9.5%) | 2 (10.5%) |
| Myocardial infarction | 1 (2.5%) | 0 (0%) | 1 (5.3%) |
| Diabetes | 24 (60%) | 12 (57.1%) | 12 (63.2%) |
| Hypertension | 37 (92.5%) | 18 (85.7%) | 19 (100%) |
| **Cause of kidney disease** | | | |
| Diabetic nephropathy | 13 (32.5%) | 7 (33.3%) | 6 (31.6%) |
| Hypertension | 12 (30%) | 6 (28.6%) | 6 (31.6%) |
| IgA nephropathy | 1 (2.5%) | 0 (0%) | 1 (5.3%) |
| Polycystic kidney disease | 2 (5%) | 2 (9.5%) | 0 (0%) |
| Obstructive nephropathy | 1 (2.5%) | 0 (0%) | 1 (5.3%) |
| Unknown | 4 (10%) | 3 (14.3%) | 1 (5.3%) |
| Other | 7 (17.5%) | 3 (14.3%) | 4 (21.1%) |
| **Chronic kidney disease stage** | | | |
| 2 | 1 (2.5%) | 0 (0%) | 1 (5.3%) |
| 3a | 8 (20%) | 5 (23.8%) | 3 (15.8%) |
| 3b | 6 (15%) | 4 (19%) | 2 (10.5%) |
| 4 | 7 (17.5%) | 3 (14.3%) | 4 (21.1%) |
| 5 | 18 (45%) | 9 (42.9%) | 9 (47.4%) |
| **Treatment modality** | | | |
| Non-dialysis dependent kidney disease | 16 (40%) | 5 (23.8%) | 11 (57.9%) |
| Kidney transplant recipient | 14 (35%) | 10 (47.6%) | 4 (21.1%) |
| Dialysis therapy | 10 (25%) | 6 (28.6%) | 4 (21.1%) |
| **HbA_1c_, mmol/mol** | | | |
| N | 6 | 3 | 3 |
| Median (IQR) | 47.0 (43.5-79.5) | 47.0 (45.0-) | 47.0 (39.0-) |
| **Creatinine, μmol/L** | | | |
| N | 40 | 21 | 19 |
| Median (IQR) | 206 (130-637) | 164 (123-650) | 208 (135-640) |
| **C-reactive protein, mg/L** | | | |
| N | 15 | 6 | 9 |
| Median (IQR) | 8 (3.0-15.0) | 10.5 (6.25-26.0) | 7.0 (2.5-19.0) |

Table S3. Secondary outcomes included within the study.

| Measure | Description |
| --- | --- |
| Kidney disease quality of life (KDQoL SF1.3) | Health-related quality of life |
| The Chalder fatigue questionnaire | Physical and mental fatigue |
| Patient activation measure (PAM-13) | Patient activation (the knowledge, skills and confidence a person has in managing their own health and health care) |
| Patient-health questionnaire-4 (PHQ-4) | Depression and anxiety |
| Sit to stand 60 | Lower body strength test |

Table S4. Illustrative quote table for intervention acceptability.

| Theme - Access to devices and data | Quote: |
| --- | --- |
| Access to an iPad | “Yeah, it’s a good idea because when you need that and you can't afford it... if you see where you can borrow, you use it, it’s good”  Female, dialysis dependent, 57 years, Black African |
|  | “Not everybody can afford to buy one... And it gets you active again. Helps you get active again to get on that programme”  Female, kidney transplant recipient, 68 years, White British |
|  | *“Well how else would I be able to do, like what to do, unless I was able to borrow it and be shown how to use it and achieve anything you know, from the iPad.”*  Male, dialysis dependent, 73 years, White British |
| Fear of damaging or losing a loaned device | “If the gadget is broken accidentally, maybe I’m going to pay for it. I’d feel bad... I don’t want to put that on myself”  Male, dialysis dependent, 60 years, Black African |
|  | “I was frightened in case I blew it up... I was worried at first”  Male, kidney transplant recipient, 75 years, White British |
|  | “I was very careful with it... No one touched it besides me. They weren’t allowed to”  Female, dialysis dependent, 58 years, Black Caribbean |
|  | “I hate it [losing the device]. If it goes missing, that’s terrible. You care for it and put it away so nobody can get it”  Female, non-dialysis dependent, 78 years, White British |
| Positives of having access to an iPad | “I was able to practise alongside what they are doing... with the iPad I was able then to do some of the exercises... I can now practise on my own” Female, dialysis dependent, 57 years, Black African |
|  | “I think I would try [to use the internet]”  Female, dialysis dependent, 64 years, Black African |

| Theme - Skills and capability | Quote: |
| --- | --- |
| Requirements for learning new digital skills | “I need someone to teach me how to do it”  Male, dialysis dependent, 60 years, Black African |
|  | “Once you taught me, I’d be alright... I wouldn’t need to keep bothering you”  Female, non-dialysis dependent, 68 years, Black Caribbean |
|  | “I don’t know how I could move on unless I was shown”  Male, dialysis dependent, 73 years, White British |
| Learning to use the iPad by repetition | “If I can’t do something, I keep on and on until I can” Female, kidney transplant recipient, 68 years, White British |
|  | “Just using it over and over again”  Male, kidney transplant recipient, 75 years, White British |
| Teaching how to use the iPad during the study | “You explained it properly... eventually I got it” Female, non-dialysis dependent, 68 years, Black Caribbean |
|  | “The session was helpful… it’s helpful if someone is with you to guide you”  Male, dialysis dependent, 77 years, White British |
| Improvements from the study | “I can now access it easily… I just get in the login details and it opens” Female, Female, dialysis dependent, 57 years, Black African |
|  | “My confidence is more… I’ve got confident now” Female, dialysis dependent, 64 years, Black African |
|  | “I could actually go online and order something… bringing technology into my life”  Female, non-dialysis dependent, 58 years, Black Caribbean |
| Springboard for future learning | “I will [improve my skills]”  Female, dialysis dependent, 57 years, Black African |
|  | “I want to keep the iPad more, to train a little bit more”  Male, dialysis dependent, 57 years, Asian |

| Theme – Beliefs and trust | Quotes: |
| --- | --- |
| Potential future use of iPad | *“Maybe for my business”*  Male, dialysis dependent, 55 years, Black African |
|  | “I can use it now to play games, do other things” Female, dialysis dependent, 57 years, Black Caribbean |
| Future Kidney Beam use | *“If I was shown how to do it, then I could most probably carry on”*  Male, kidney transplant recipient, 75 years, White British |
|  | *“If I didn’t feel like coming out, I’d stick with the app”* Female, non-dialysis dependent, 65 years, Black Caribbean |
|  | “I want to continue... I like it” Female, dialysis dependent, 64 years, Black African |
| Conditional future Kidney Beam use | “Depends on what they got to offer me”  Male, dialysis dependent, 73 years, White British |
|  | *“Yes, but not at the moment... it depends on you people”*  Male, non-dialysis dependent, 70 years, Black African |
| Trusting the NHS | *“I think it’s a good idea by the NHS, yeah.”*  Male, non-dialysis dependent, 67 years, Black Caribbean |
|  | *“it’s OK to borrow things on the NHS… So yes, I think it’s a good idea to be that people are able to reach out and get things…”*  Female, non-dialysis dependent, 69 years, Black Caribbean |

| Theme – Leadership and partnerships | Quotes |
| --- | --- |
| Plea to organisations and funders to promote widely | “Try and fund it so that every other person can enjoy it”  Female, dialysis dependent, 57 years, Black African |
|  | “It could help a lot of people in lots of different ways” Male, kidney transplant recipient, 75 years, White British |
| Flexibility of borrowing an iPad and using Kidney Beam | “You do it when you want… at night, day, or when you are free”  Male, dialysis dependent, 57 years, Asian. |
|  | “You get information and you can do things and don’t rely on people”  Female, non-dialysis dependent, 80 years, Asian |
| Support from the research team | “Make sure you’re doing the right ones… you do forget”  Female, dialysis dependent, 58 years, Black Caribbean |
|  | “It’s helpful by reminding people that… you can help yourself”  Male, non-dialysis dependent, 70 years, Black African |
|  | “If they teach that person then they have good knowledge”  Male, dialysis dependent, 60 years, Black African |
| Peer encouragement | “I would say to other patients go for it. It’s worthwhile”  Female, kidney transplant recipient, 68 years, White British |

Table S5. Qualitative topic guide:

Topic Guide for Ex-Tab interviews V1.3

| **Introduction and welcome** |
| --- |
| Thank you for agreeing to speak with me again.  I’m interested in hearing about your experiences, thoughts and comments both positive and negative of using an iPad to exercise.  Before we begin, I would like to remind you that whatever you say here will be confidential. Your name/s and personal details will not be mentioned in any report. If you want to stop the interview at any time, that’s fine. The interview should last around an hour, is that ok?  I am using a digital recorder to record our conversation/ going to record the virtual meeting because it is difficult for me to write down everything you say. This will also enable me to give you my full attention and listen to what you say.  Any questions before we begin? |

| **Opening questions**  **Includes beliefs and trust**  **Definition:** so that people understand and feel confident using digital health approaches: |
| --- |
| **Both groups:** What was your experience both positive and negative of using technology prior to taking part in this study? e.g., shopping online, internet banking, social media? Tell me above how confident you are doing these things online? Has anything or any one helped you get more confident?  ***Prompts:***  *-What access to devices do you have? Wifi at home? Data? What do you use these devices for e.g. shopping, social media, facetime*  *-How were your skills? And confidence? What was lacking?*  *-Experience of NHS app or my chart? Why not?*  *-Any concerns / worries technology? Any fears? Any bad experiences?* |
| **Accessibility and ease of using technology**  Definition: so that user-centred digital content and products are co-designed and deliver excellent patient outcomes |
| **Intervention group**: You were given an iPad to use for exercise and then taught how to use the iPad to access the Kidney Beam website… tell me about this experience? What went well and not so well?  **Non-intervention group:** You were not given an iPad to borrow but were given the sign up instructions for Kidney Beam… tell me about your experience of using it? What went well and not so well?  ***Prompts:***  *-Could this be improved? What did you find most useful / helpful?*  *-What did you like and not like?*  *-Did we cover everything to enable you to Kidney BEAM?*  *-Did any family or friends help you to use it? (Explore support at home)*  **Follow up INTERVENTION:** Reflecting on your experience, what advice would you give us, to help us roll this out? What worked well? What could be improve? Any suggestions ?  **Prompts:**  -Thoughts around short courses? Lessons? Other ideas?  **Follow up**: Did you come to TJHC for tech support? What were your thoughts on coming to TJHC? Would you have liked a different setting?  **Non-intervention group**: what support would you have needed to help you use Kidney Beam? Coming into TJHC for tech support? Anything else or other ideas?  ***Prompts:***  *-Was this helpful? Why/not?*  *-If not, would you have liked to come? Was it offered?*  *-How would you like to receive tech support in the future?* |

| **Skills and capability**  Definition: so that everyone has the skills to use digital approaches and health services respond to the capabilities of all. |
| --- |
| **INTERVENTION:** You have had access to an iPad for over 12 weeks, reflecting on your experiences tell we about your confidence levels? Have the improved? Have you learned any new skills? Are you using the internet more? If so, what are you using it for?  **NON-INTERVENTION**: What skills do you think you need to improve for your confidence with online technology? How can these be improved?  **INTERVENTION**: Being able to access Kidney Beam via the I-PAD has it helped you? If so, how? Why? can you give some examples..  **NON-INTERVENTION:** How did you feel not getting the iPad? Do you think it would have helped you to get on? Why?  ***Prompts:***  *-Any changes? Better/worse? Why do you think that is?*  *-Is there anything more that we could have done to help?*  *-What was the biggest challenge about using it?*  **INTERVENTION Follow up:** You were only able to access the Kidney Beam website during this trial, how would you feel about using an iPad to do other things E.g. shopping, browsing, video calls etc. in the future?  **NON-INTERVENTION:** How will you use technology in the future?  ***Prompts:***  *-Why…*  *-What further skills do you need?* |

| **Access to devices and data**  Definition: so that everyone can access digital healthcare if they choose to and experience the benefits |
| --- |
| **INTERVENTION:** You have now returned the iPad that you borrowed, do you plan to use a digital device in the future?  ***Prompts:***  *-If yes, how?*  *-If no, why not?*  *-Would you purchase a device? If not, why?*  **BOTH GROUPS**: Going forwards, (how) do you plan to use Kidney Beam?  ***Prompts:***  *-Will you continue? How does this look in your lifestyle? Why not?*  *-Are there any barriers to you continuing?*  **BOTH GROUPS**: What are your thoughts on borrowing equipment from the NHS?  ***Prompts:***  *-Good / bad idea? Why?*  *-Any suggestions?* |

| **Leadership and partnerships**  Definition: so that digital inclusion efforts are co-ordinated and help to reduce health inequalities. |
| --- |
| **BOTH GROUPS**: Do you think loaning iPads and teaching people how to use them is a good idea? Why?  ***Prompts:***  *-Is there a better way? How can this be improved?*  *-What else can be done to help patients get online? We do not want people falling behind digitally and ideas how to reduce this…?*  **BOTH GROUPS Follow up:** In an ideal world, how would you like to access healthcare?  ***Prompt:*** *F2F, apps, telephone? Why is this? Has this changed since taking part?*  Is there anything else that we have not spoken about that you would like to share?  End: I have come the end of the questions, thank you so much for your time. |
