# Supplementary material for: A Digital Inclusion Intervention to Improve Access to a Digital Health Intervention Among Digitally Excluded Adults: Mixed Methods Pilot Randomized Controlled Trial
Source: JMIR Form Res. 2026 Apr 16;10:e91438. doi: 10.2196/91438 (PMC13085982; doi:10.2196/91438)

Kidney Beam website

Live classes:


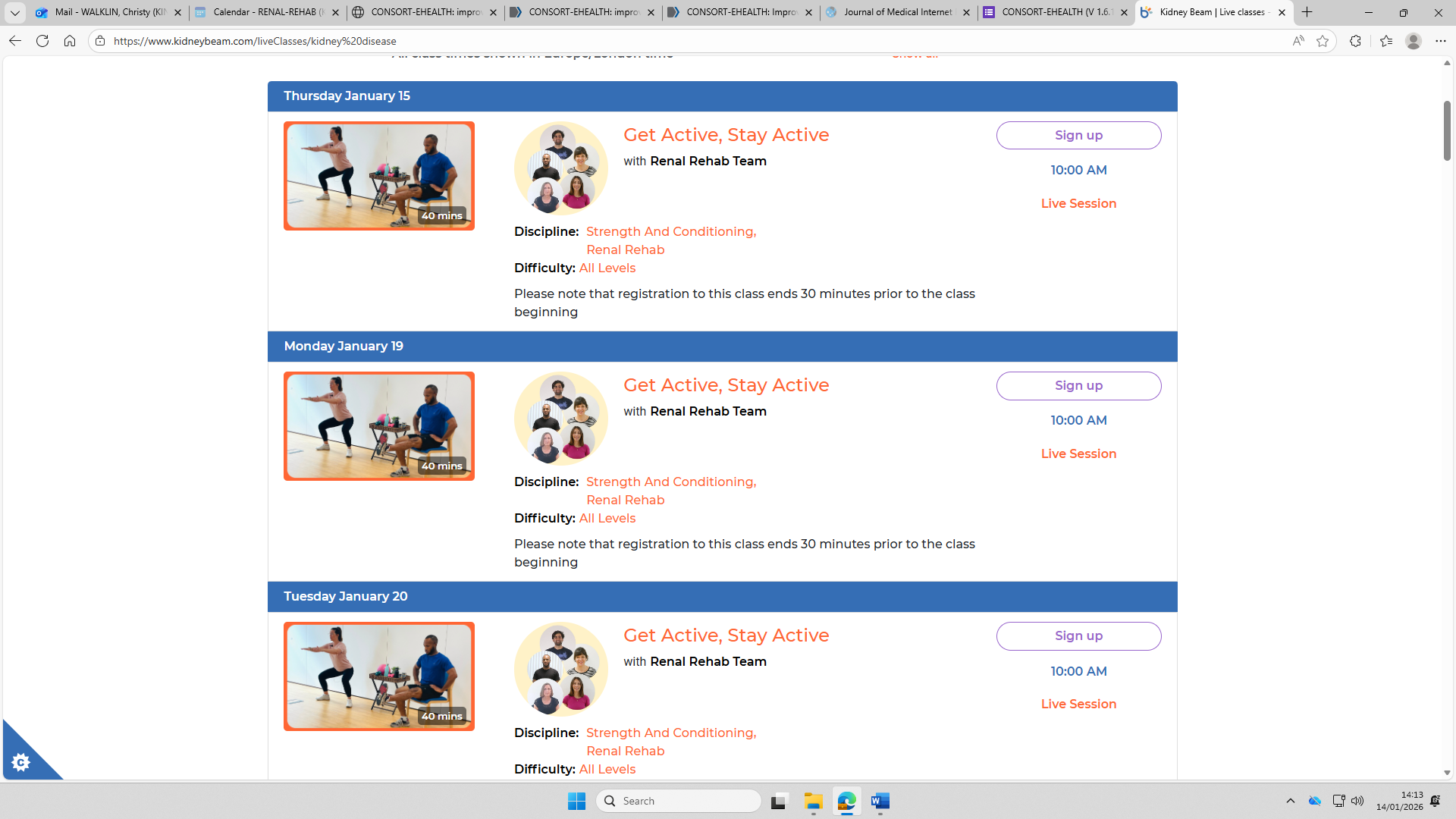


On demand programme: Seated Sessions
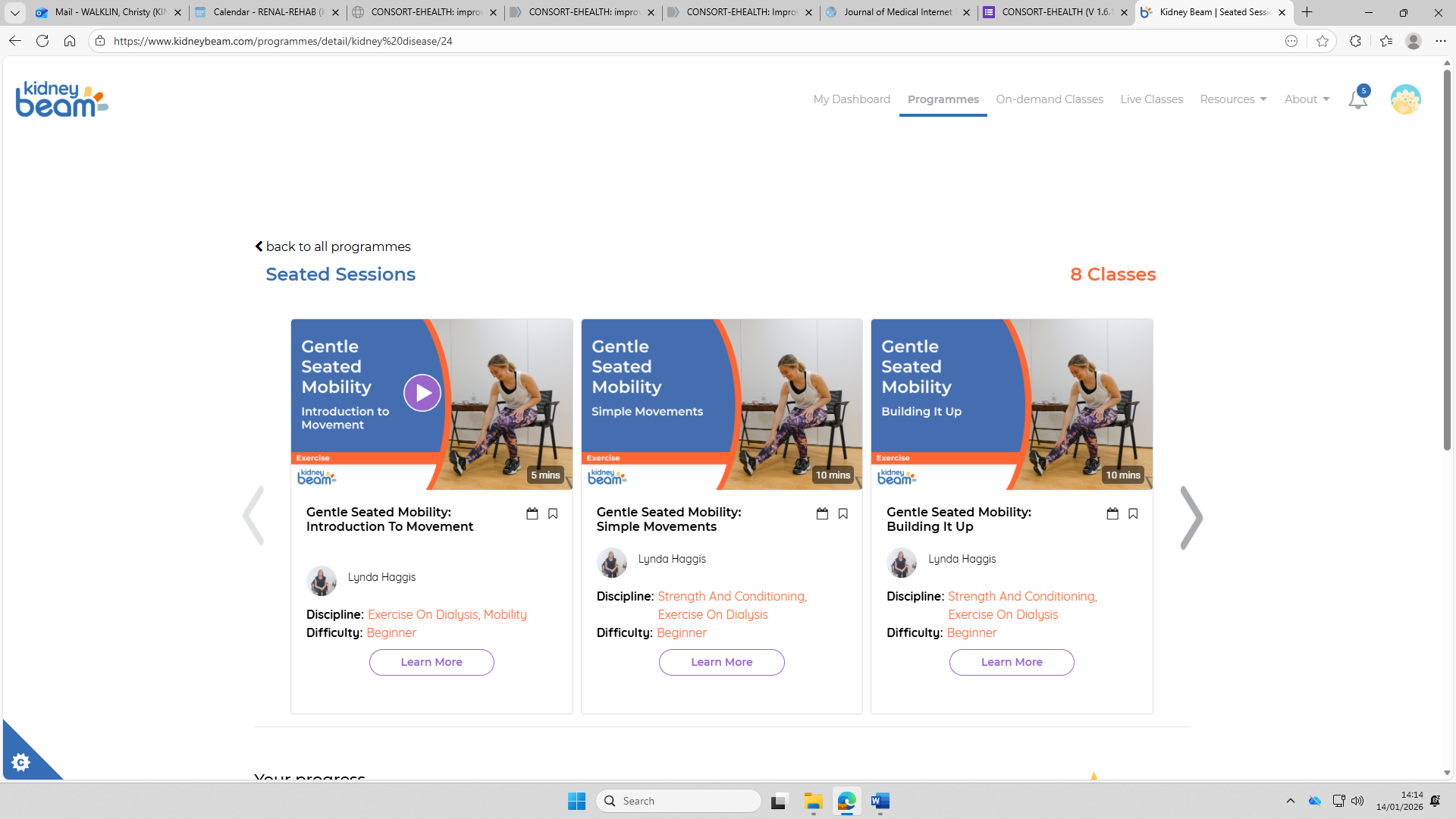


On demand programme: 12 Week Kidney Rehab Programme


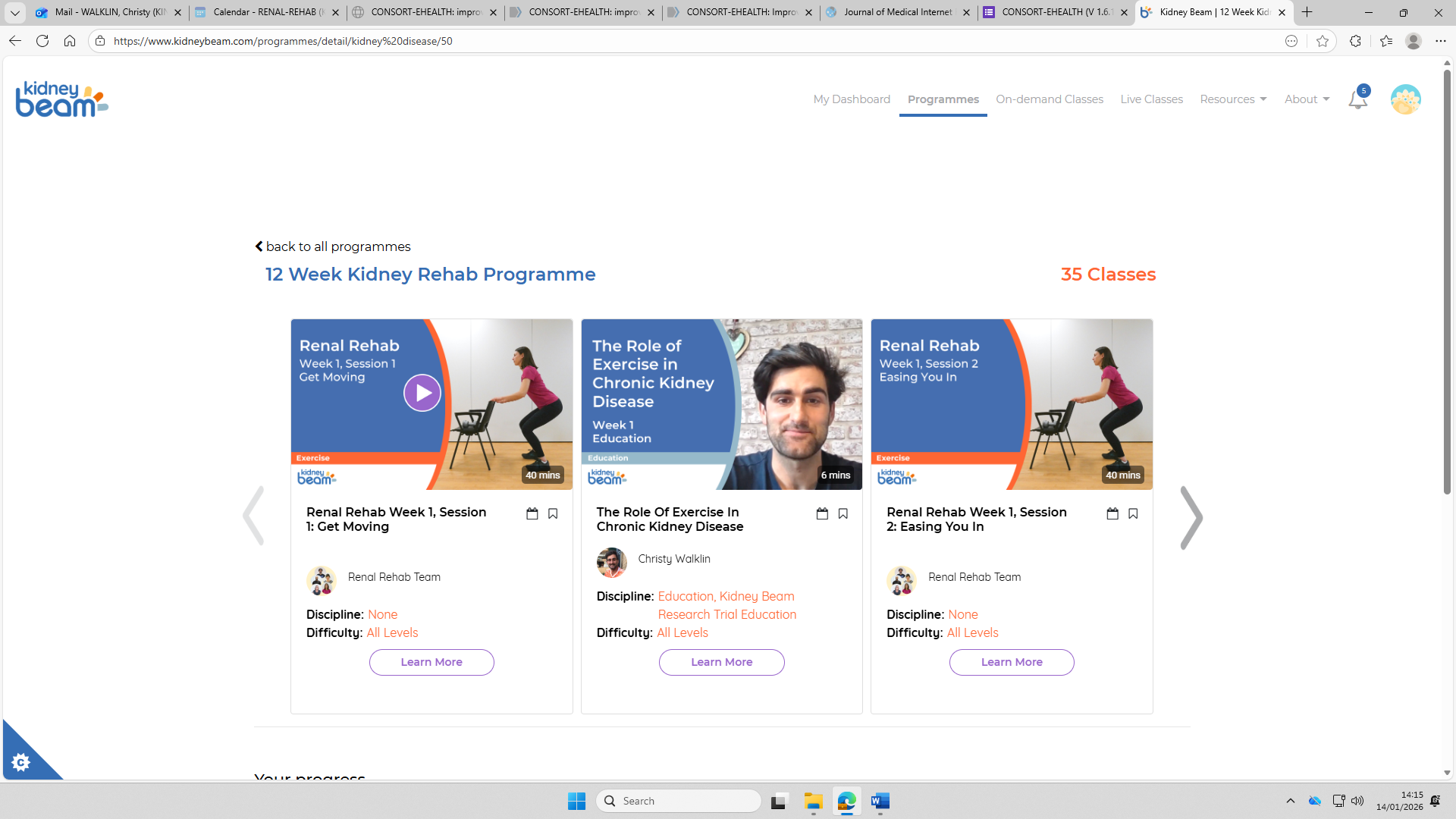

Supplement: Multimedia Appendix 4 [file formative-v10-e91438-s004.docx]
