# Supplementary material for: A Digital Inclusion Intervention to Improve Access to a Digital Health Intervention Among Digitally Excluded Adults: Mixed Methods Pilot Randomized Controlled Trial
Source: JMIR Form Res. 2026 Apr 16;10:e91438. doi: 10.2196/91438 (PMC13085982; doi:10.2196/91438)
Supplement: Multimedia Appendix 5 [file formative-v10-e91438-s005.docx]

Kidney Beam iPad user guide (EX-TAB)

STEP 1) Start by turning the iPad on. Hold down the top right button. You will see an Apple come on the screen. If the iPad is already turned on, press the top right button once.


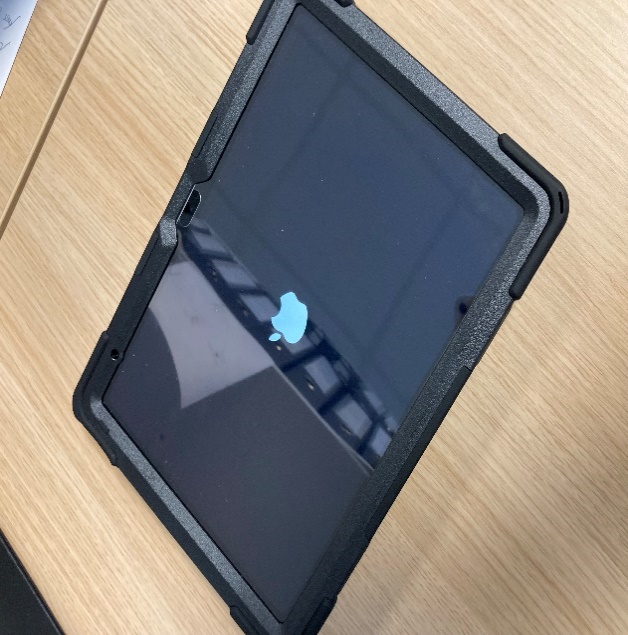

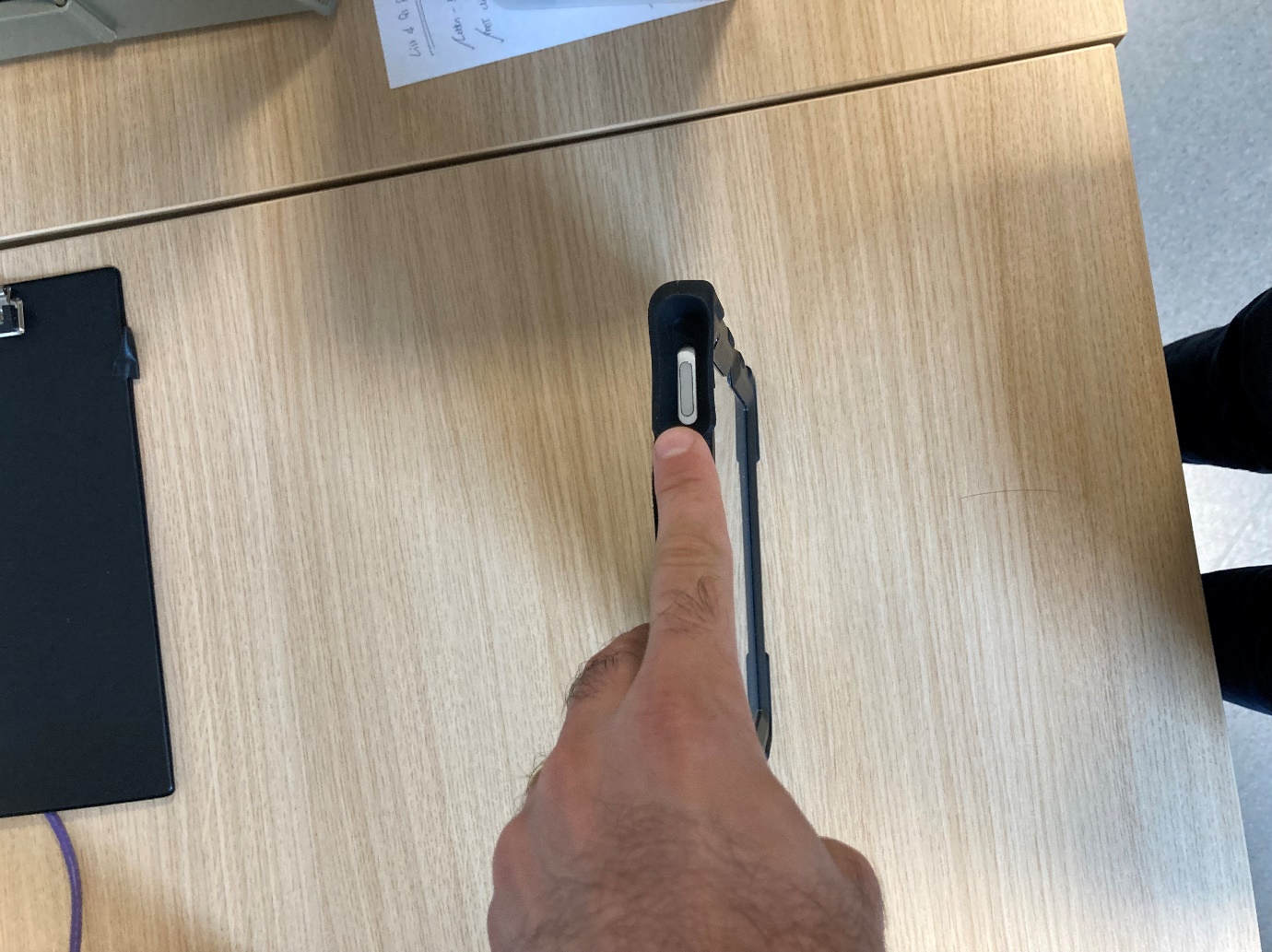


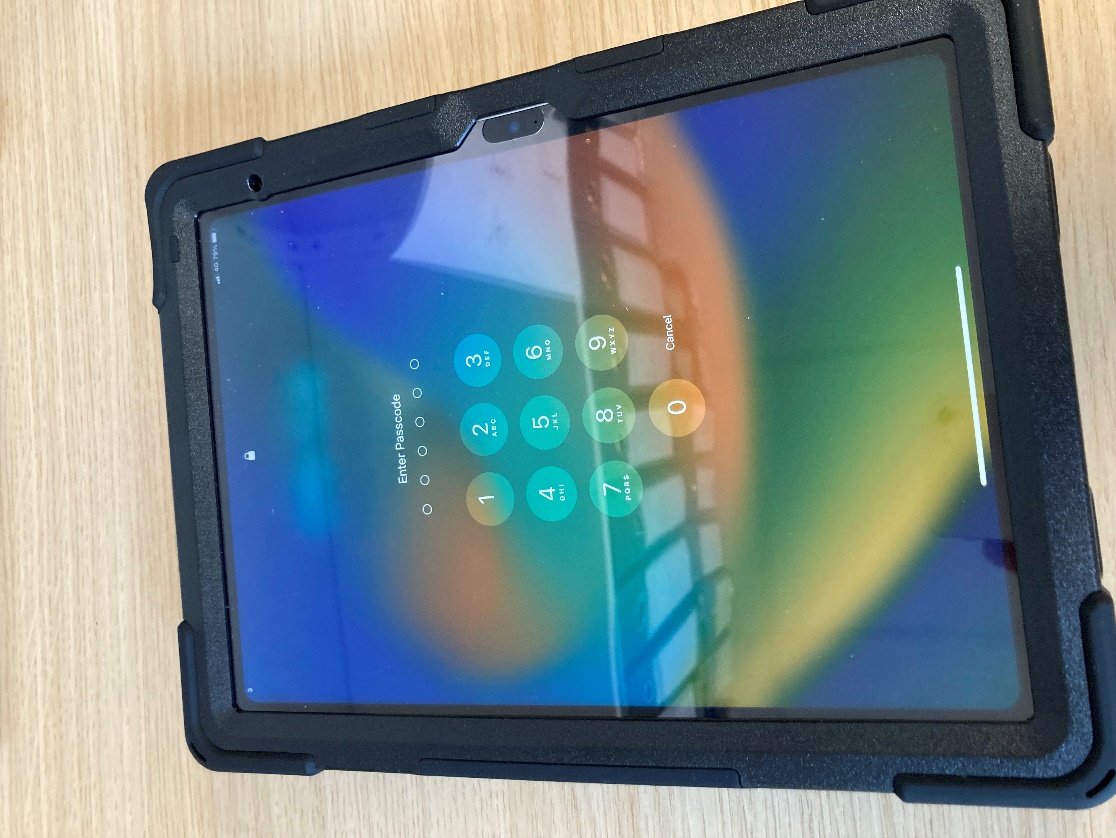
STEP 2) You will enter the lock screen. Swipe from the bottom of the screen to the top. Enter **123456** as the passcode.


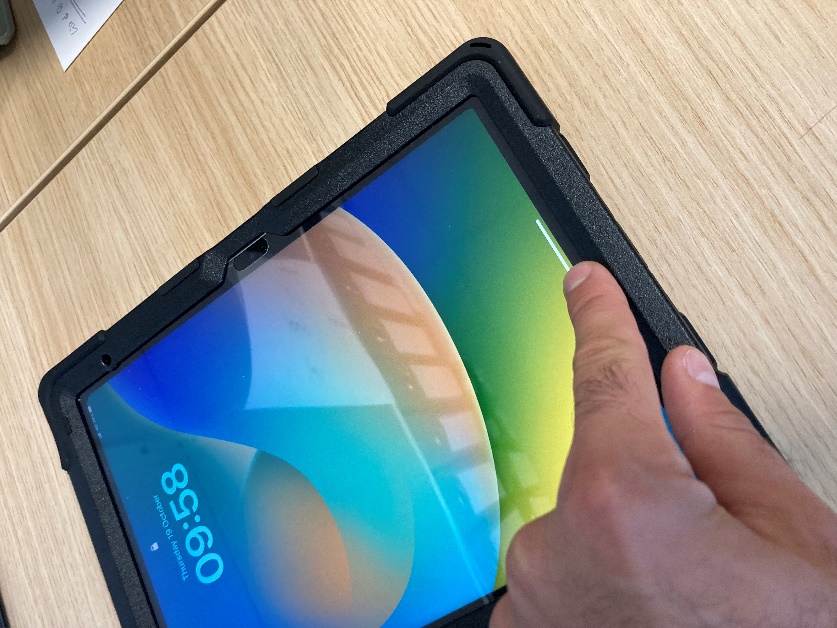

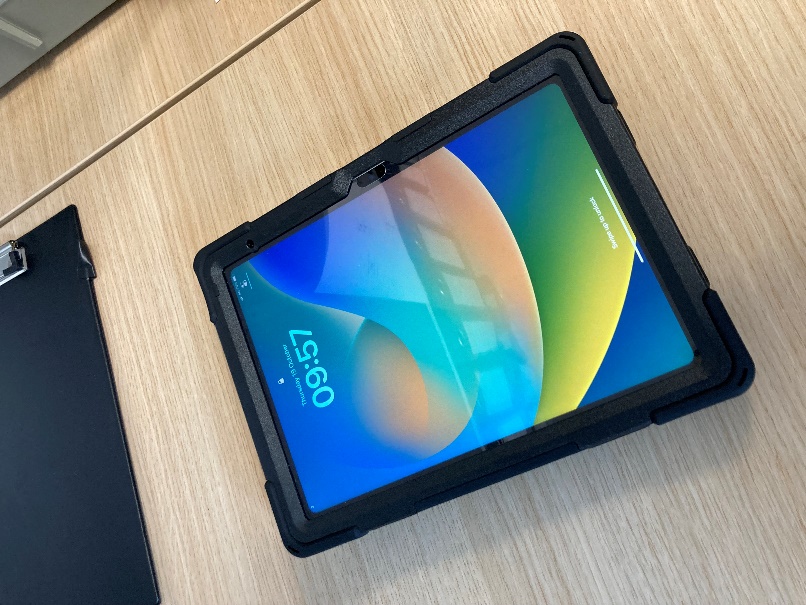


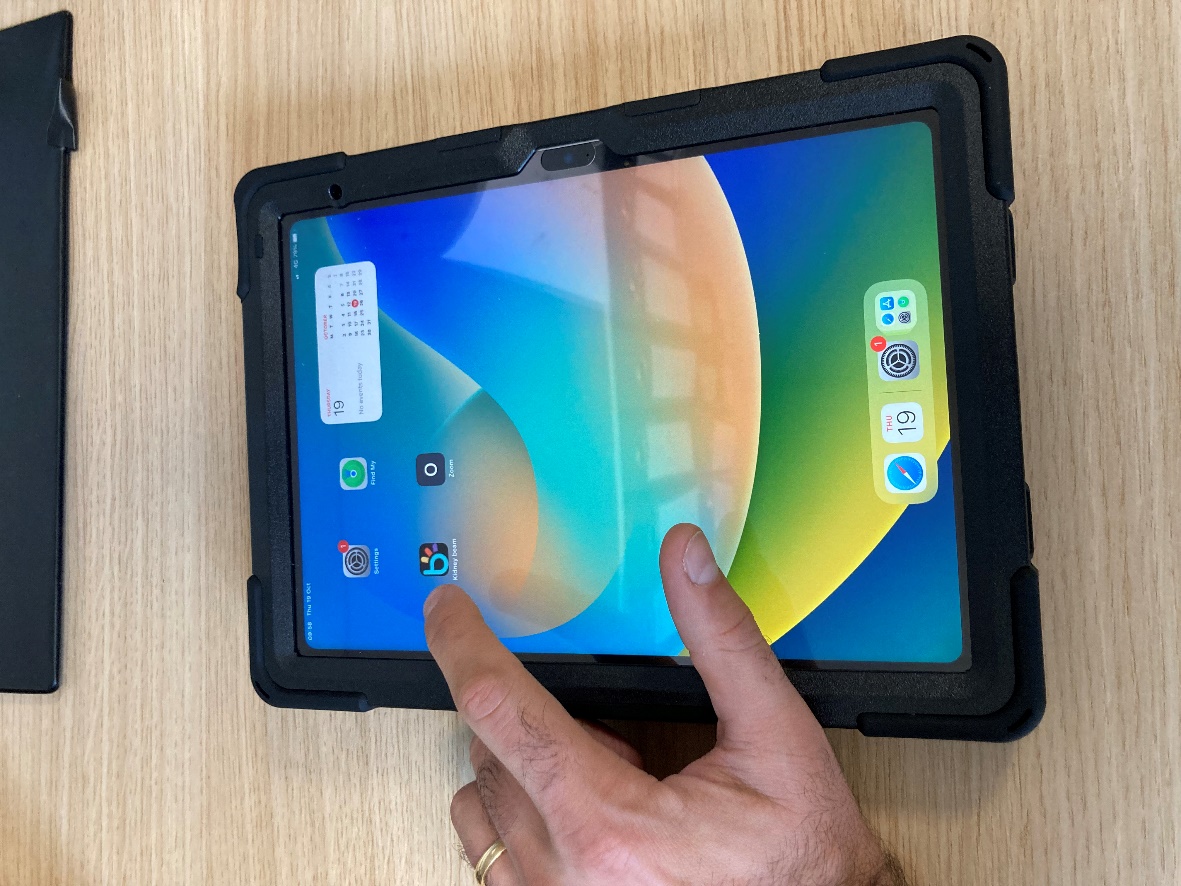
STEP 3)

Click on the Kidney Beam app.

When on the Kidney Beam app have the iPad in a **LANDSCAPE** position.

STEP 4) You should already be signed into the iPad, if not click sign in and enter your email and password. When you are ready to exercise, click on ‘On-demand classes’ on the top bar.


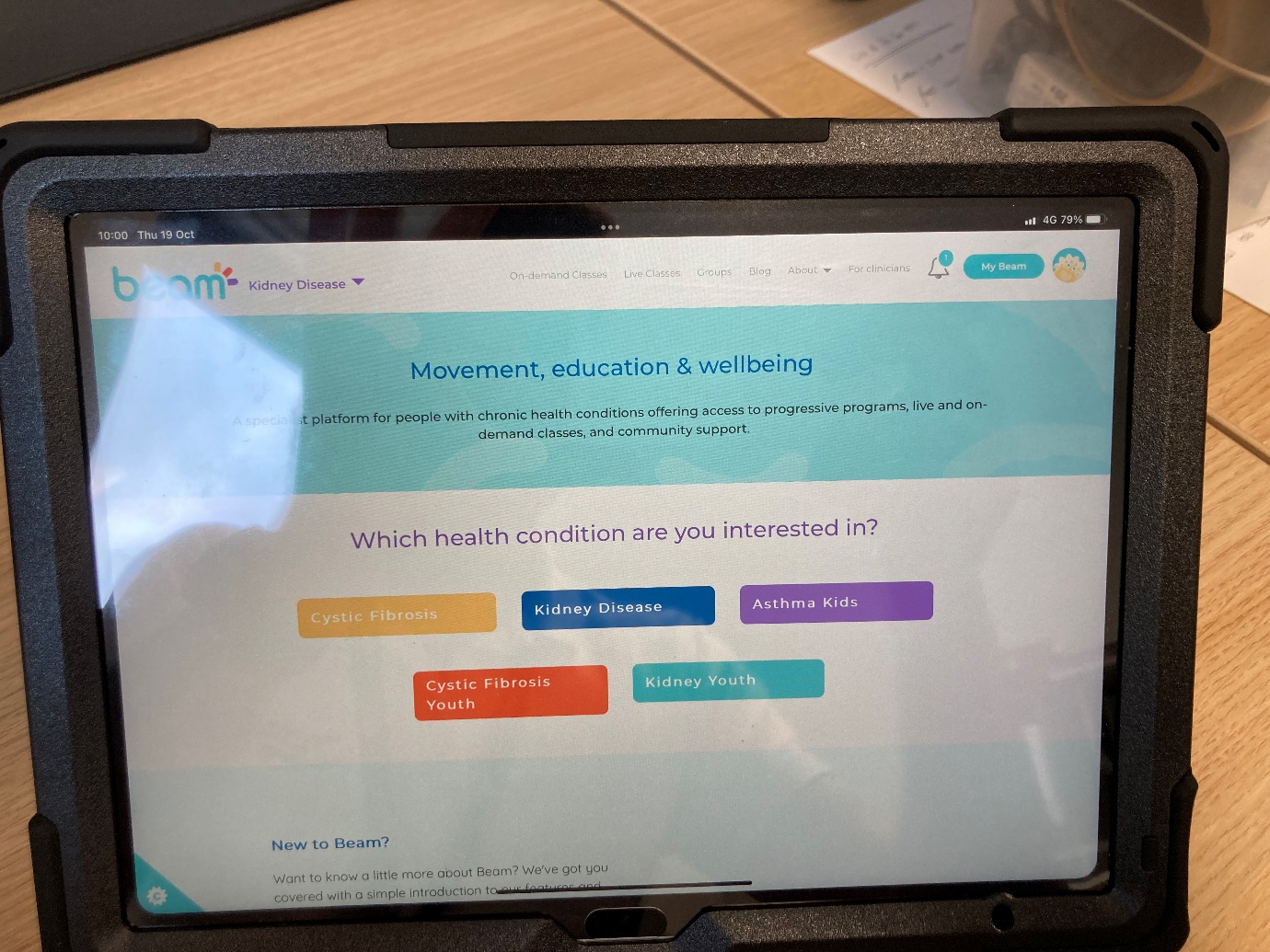


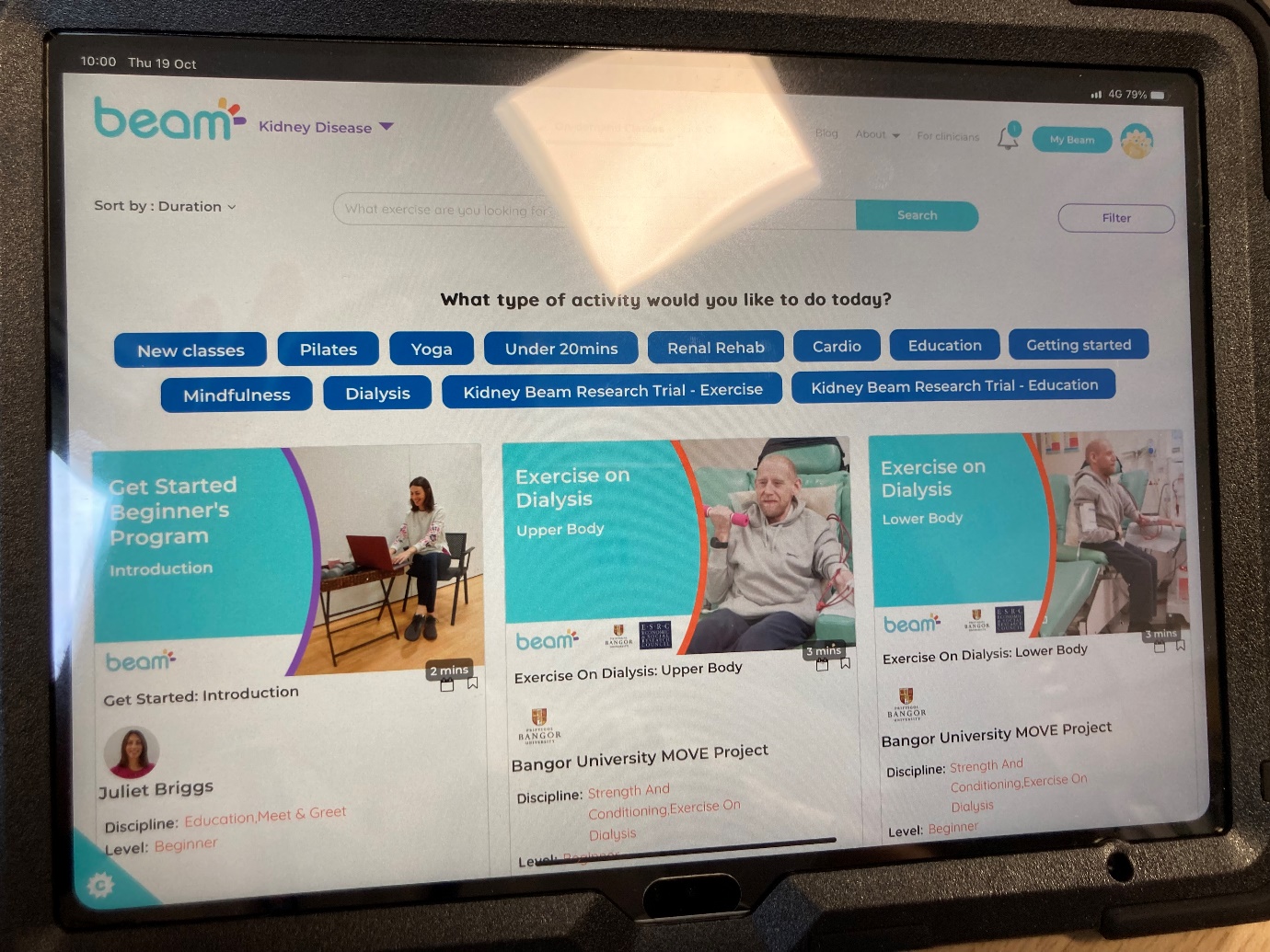


STEP 5) Click ‘Kidney Beam Research Trial – Exercise’ – For exercise sessions.

Click ‘Kidney Beam Research Trial – Education’ – for education sessions.


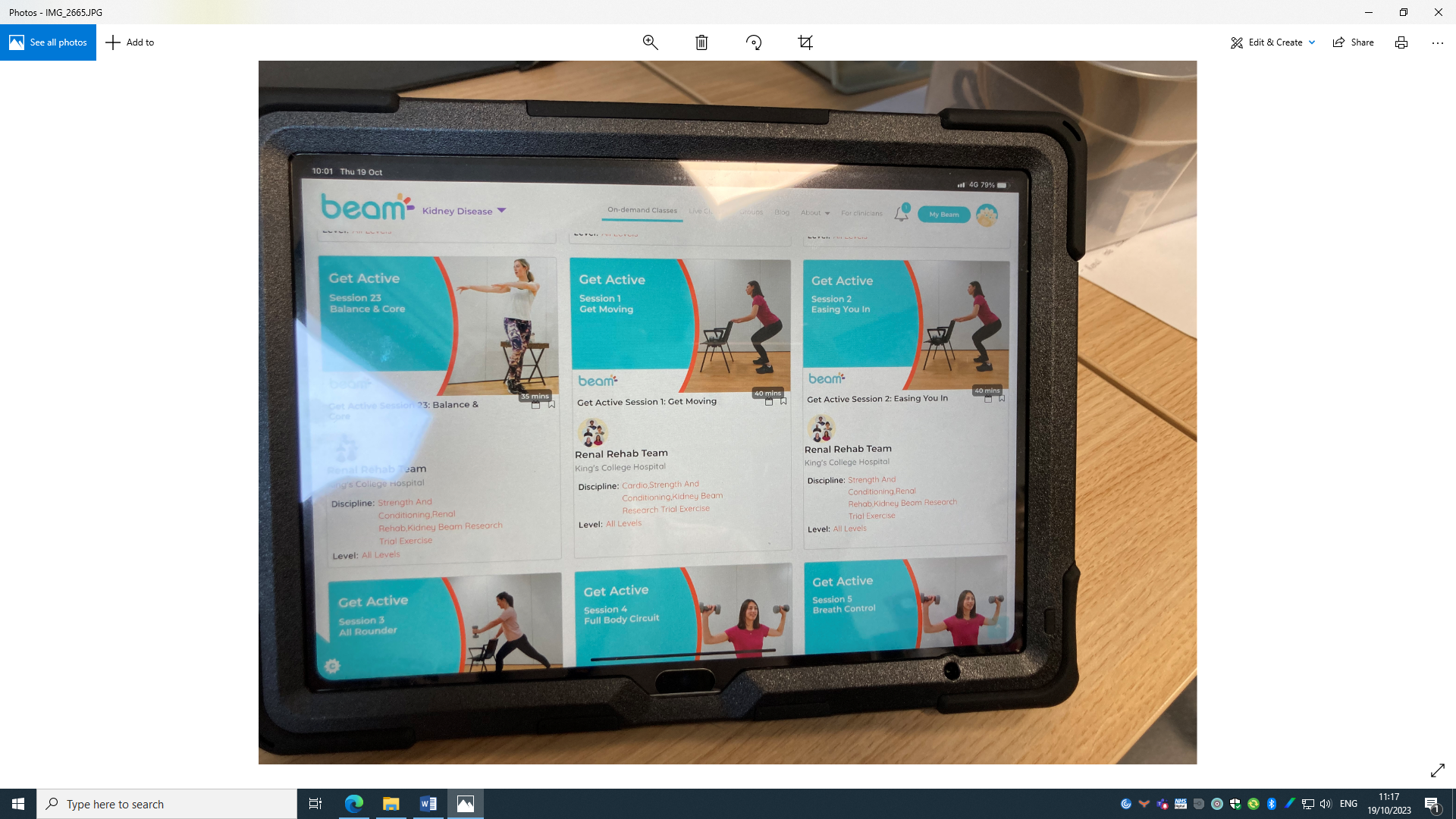
STEP 6) Scroll down to ‘Get Active Session 1: Get Moving’ and work your way through all the sessions available. Aiming for two per week. At the bottom of the page you can click ‘Show more on-demand videos’ to see more videos.

STEP 7) Click once on the play button to start exercising. Before you start enter a number between 1-10 for How good do you feel right now?


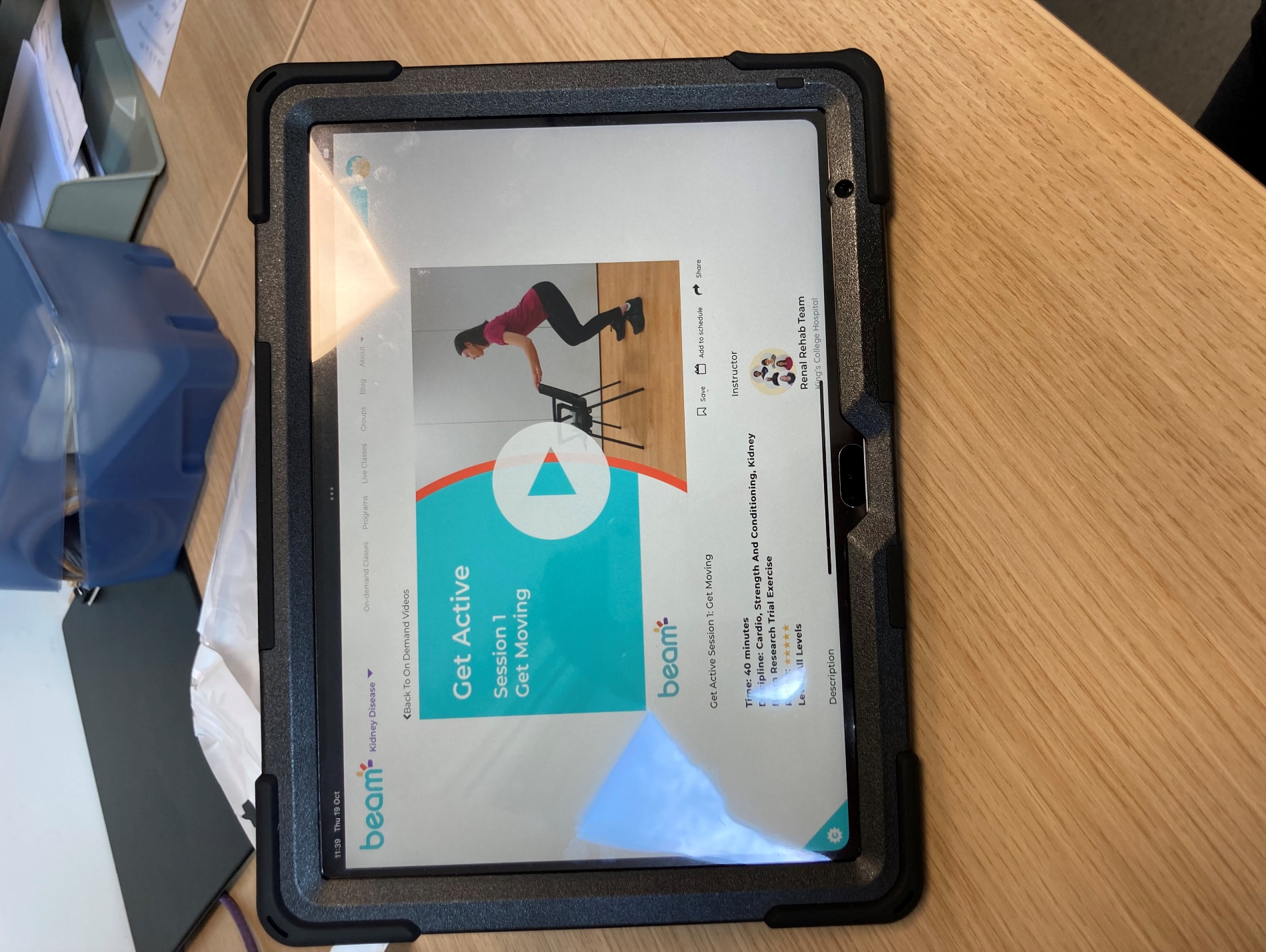


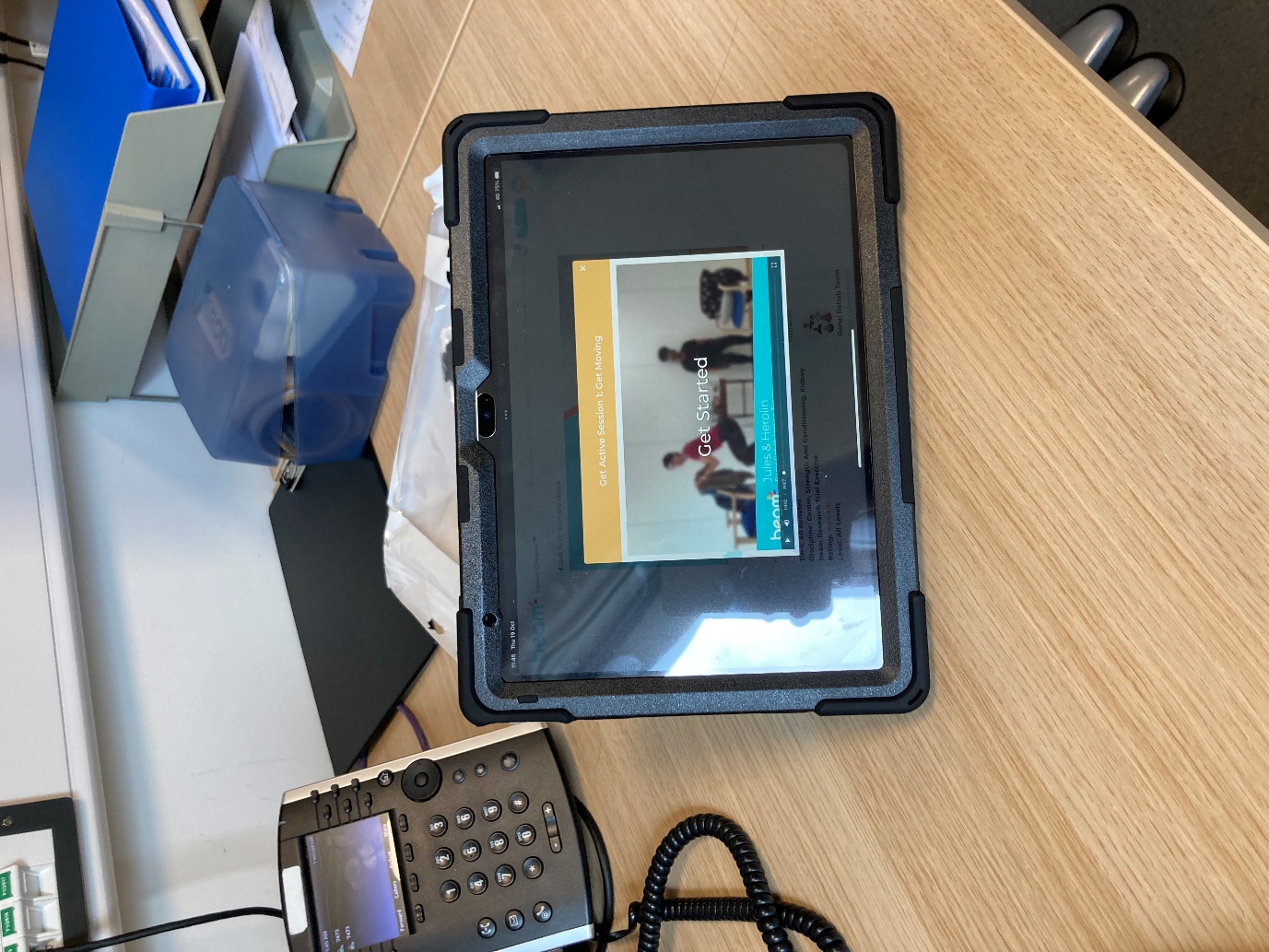
Volume controls on iPad To close the video and return click the X here

To enlarge the video, click here.

To play and pause the video, click here


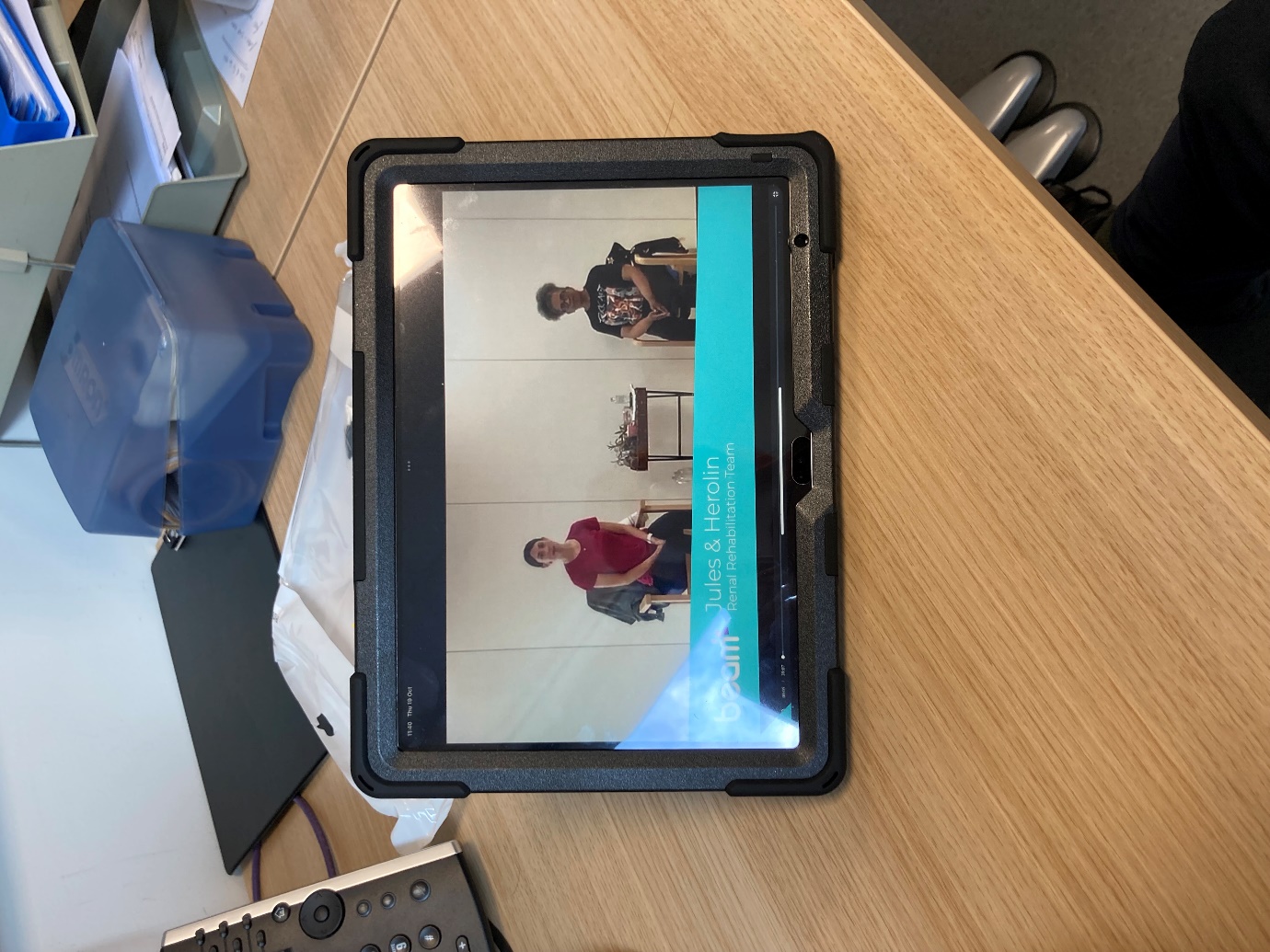


Above is the enlarged video view. To minimise click the bottom right button. Play and pause is in the bottom left corner.

Connecting to home wifi

Click on the settings button on the home page. Click Wi-Fi then choose your home wifi and add password. You will then be connected to your home wifi.


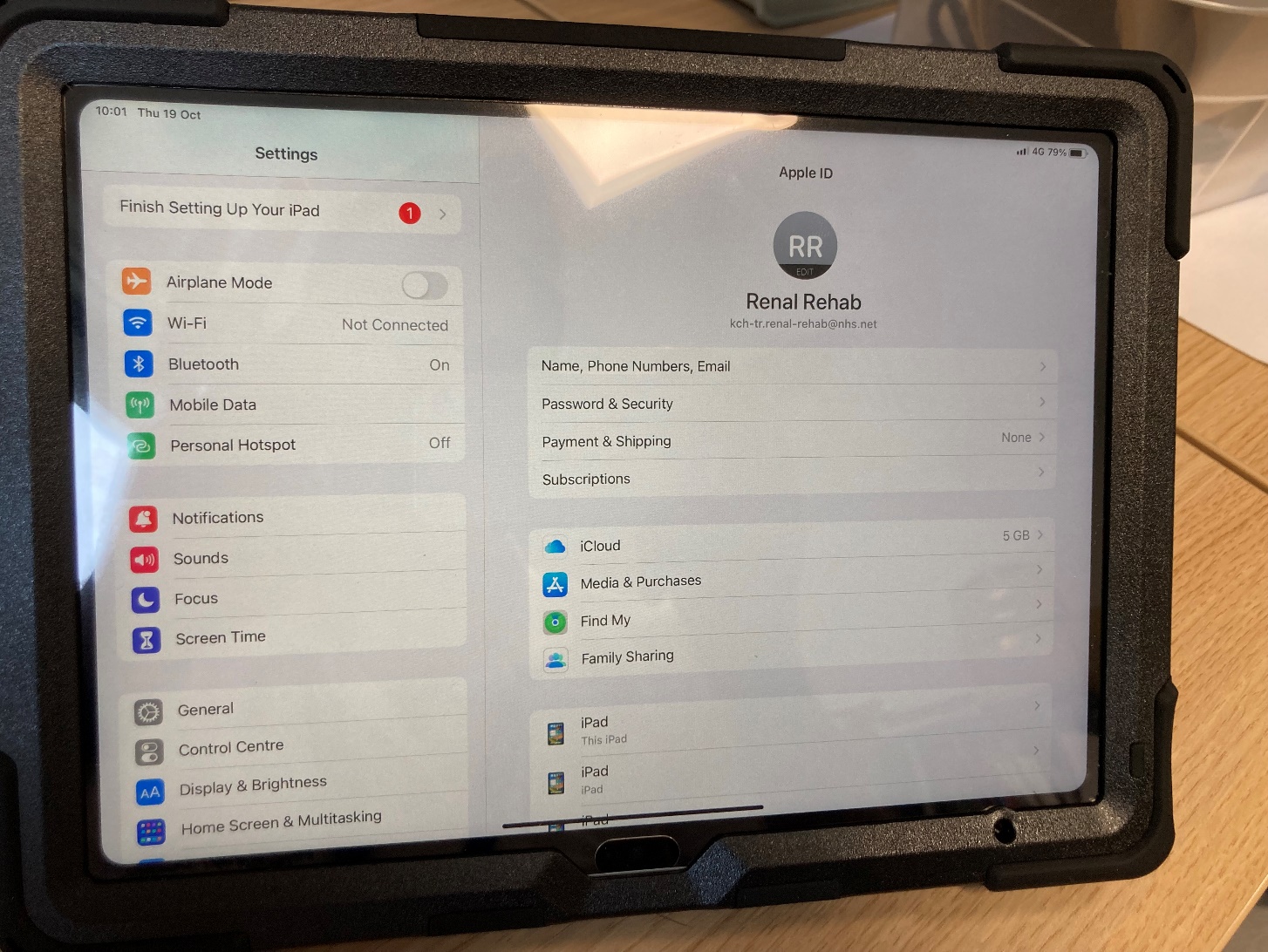

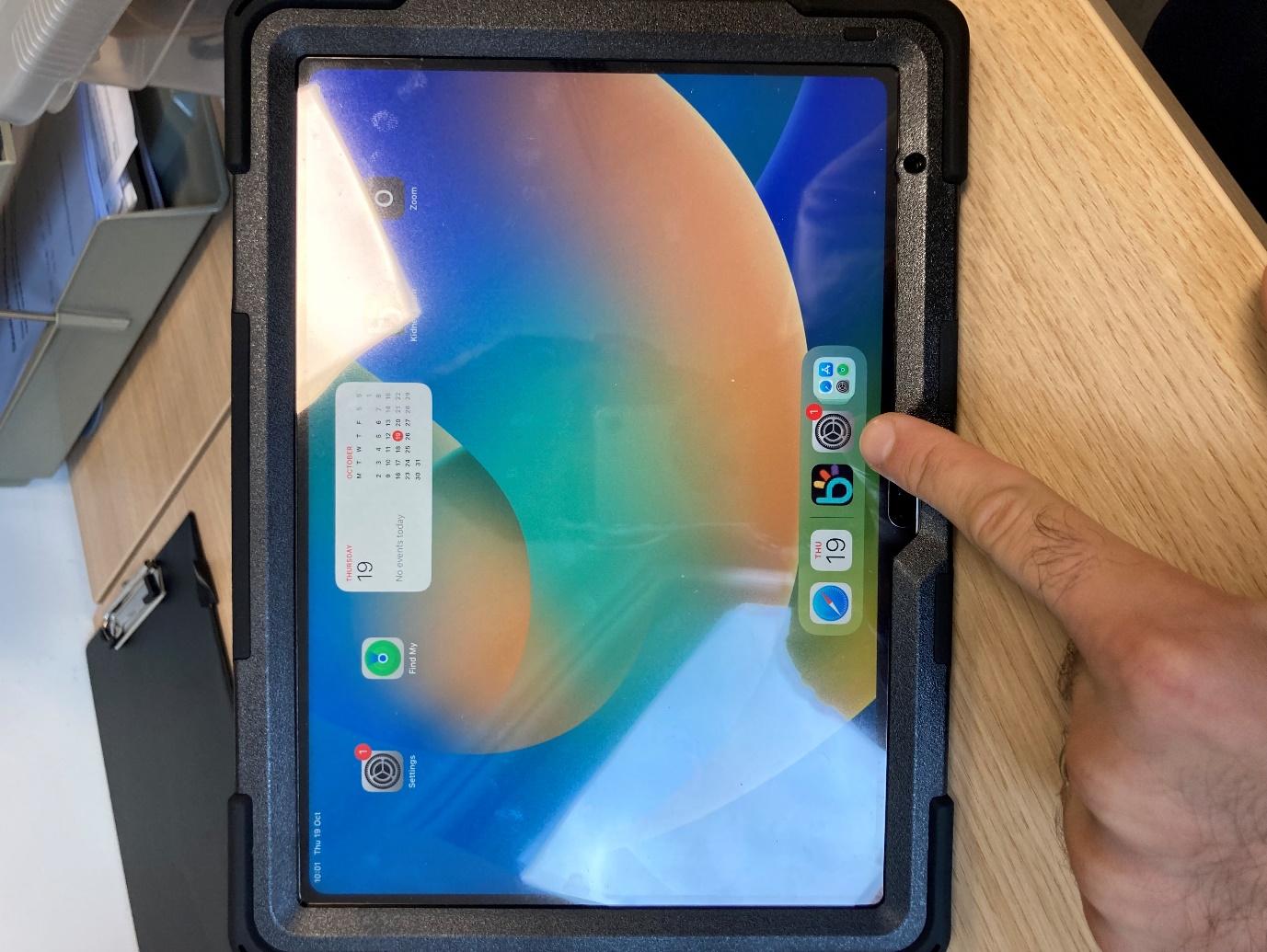


Turning off the iPad:

In settings click general and on the right side of the page scroll down to the bottom and you will see ‘Shut Down’.


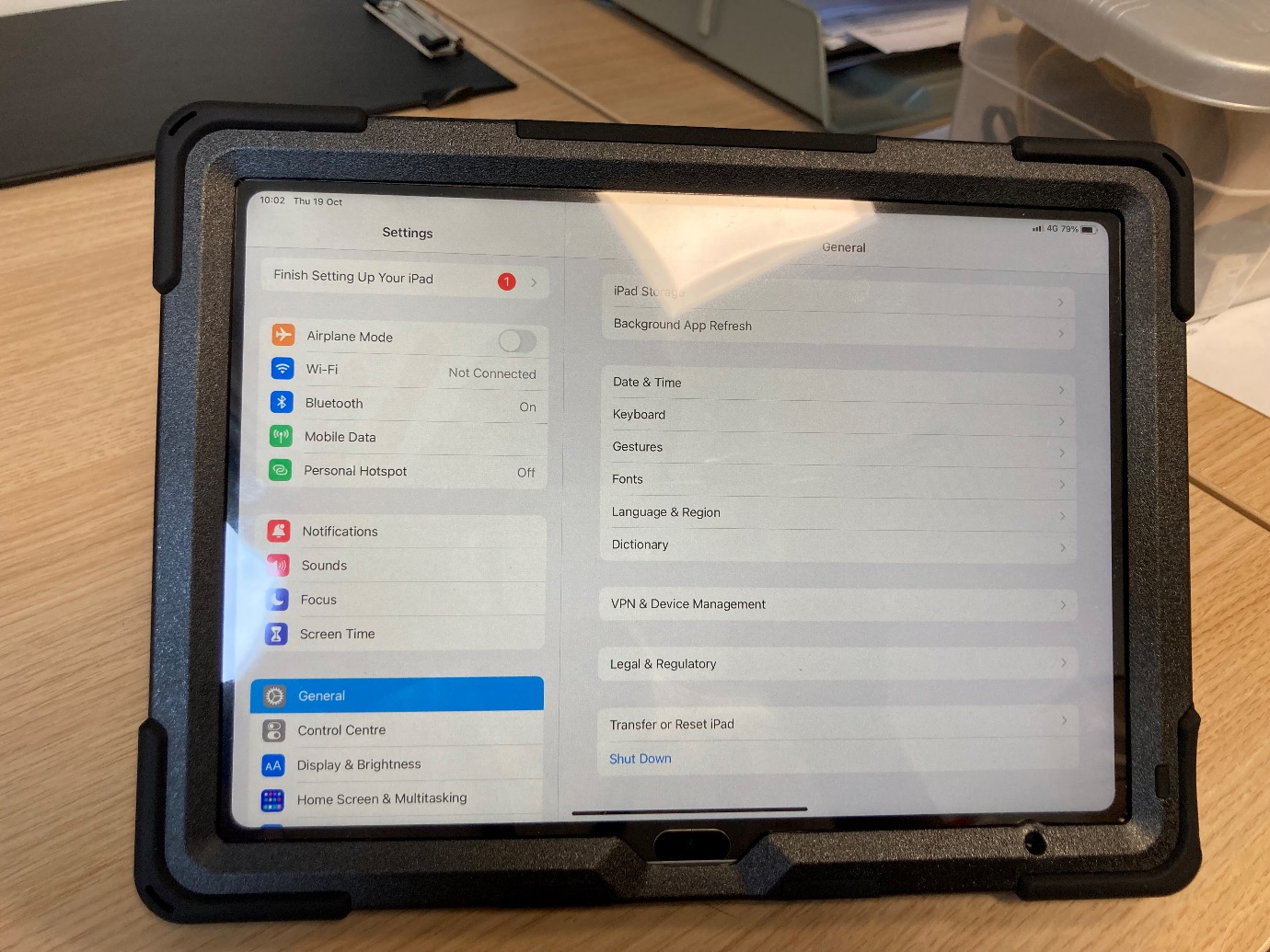


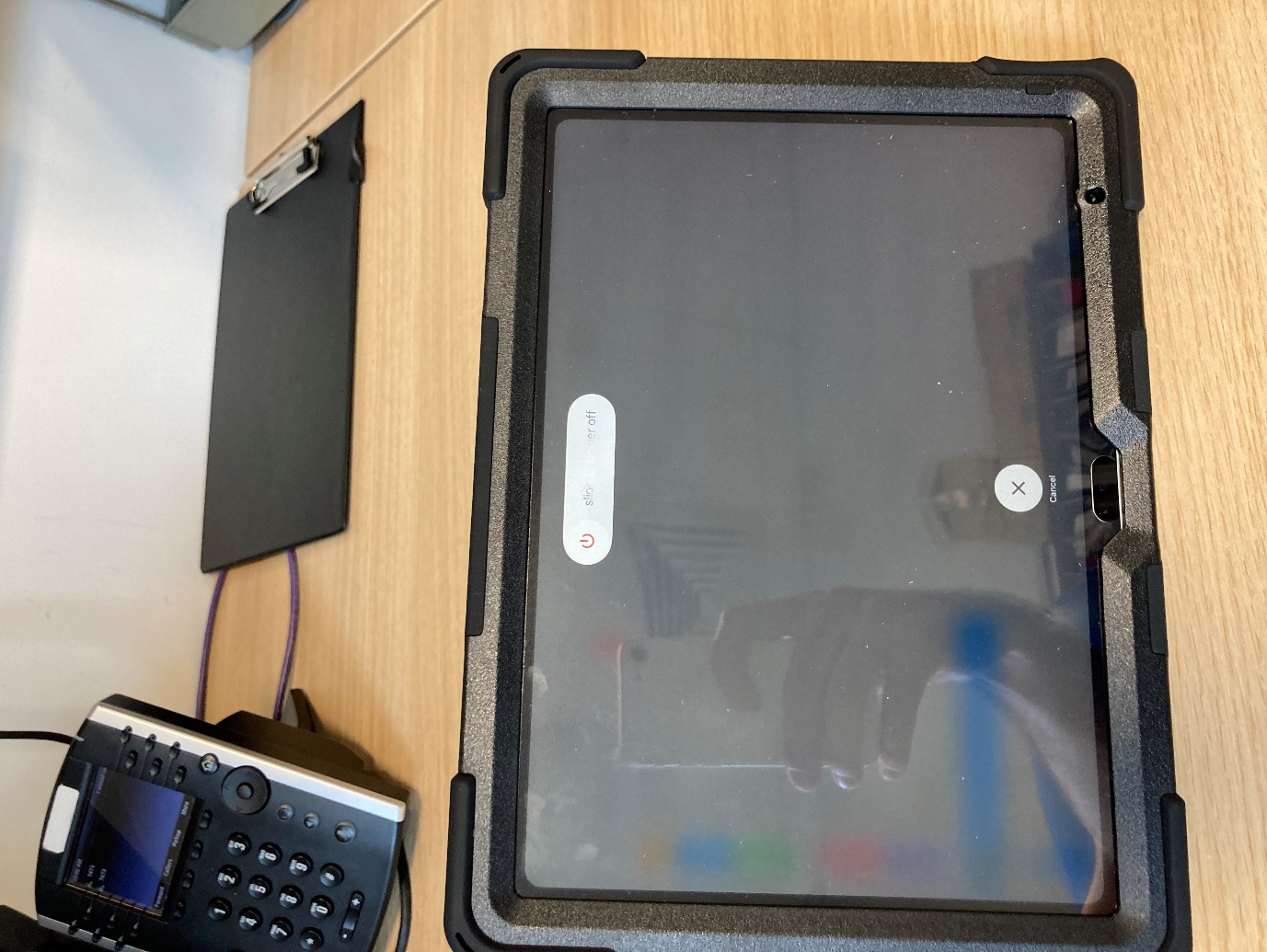


Scroll from left to right to turn off the iPad.

If you have any questions please call 020 8194 7470 and speak to a member of the team.
