# Supplementary material for: A Digital Inclusion Intervention to Improve Access to a Digital Health Intervention Among Digitally Excluded Adults: Mixed Methods Pilot Randomized Controlled Trial
Source: JMIR Form Res. 2026 Apr 16;10:e91438. doi: 10.2196/91438 (PMC13085982; doi:10.2196/91438)
Supplement: Multimedia Appendix 6 [file formative-v10-e91438-s006.doc]

**Patient name:**

**Hospital no:**

**Ex-Tab ID:**

**Re: Exercise iPad on loan**

Thank you for agreeing to take part in **The Kidney Beam – Ex-Tab Sub study**.

Research is an important way of helping us to improve the medical treatment offered to renal patients and therefore we value your participation in the study, and hope that you will enjoy your involvement.

For the purposes of the Ex-Tab study you are being provided with an Exercise iPad. This is on loan and should be used solely to access the Kidney Beam online exercise and education platform (see below)

| What the device **can** be used for… | What the device **cannot** be used for… |
| --- | --- |
| Live and on demand Kidney Beam classes **only** | Any personal use e.g. banking, shopping, gaming, email communication… (this list is not exhaustive) |

Please note that each Exercise iPad has a remote software management system installed to enable the Research Team to solve any problems with the device remotely if you need help.

The Team will also be able to see the location and track the use of the Exercise iPad to help in the event of loss or theft.

The remote management software installed will add restrictions to the device to keep you safe online.

**The Exercise iPad you are being loaned for the Ex-Tab study is:**

**Number: Serial no:**

**The Exercise iPad (as listed above) will remain the property of King’s College Hospital and**

**I agree that when I have completed the Ex-Tab study I will return the listed Exercise iPad on the agreed date to a named staff member.**

Patient Name: ……………………………………………………. Date:

Signature: ………………………………………………..

Name of issuing person: ………………………………………… Date:

Signature: ………………………………………………..

**Useful information**

You will have been shown how to access and use your Exercise iPad by the Physiotherapist at your baseline assessment, however, should you experience any technical difficulties, require any further advice or guidance about using the Exercise iPad, please call **020 8194 7470** between 08:00 – 4:30 Mon – Fri

for assistance.

At assessment the Physiotherapist will have discussed the live and on demand exercise sessions and education options with you. They will also have shown you how to log any other activity you do off the platform into the online Activity Diary

If you have any questions about any of these or anything else, such as the appropriate level of exercise intensity you should aim to achieve when exercising, then don’t hesitate to give the Team a call on the telephone number above and we will be happy to help.

**The Renal Exercise and Research Team**
